# Probiotic–Plant Bioactive Synergy in Gut Health: Mechanisms, Antimicrobial Activity, and Translational Challenges

**DOI:** 10.3390/nu18132112

**Published:** 2026-06-28

**Authors:** Monika Elżbieta Jach, Ewa Sajnaga, Ewa Ozimek, Anna Serefko, Marcello Locatelli

**Affiliations:** 1 Department of Molecular Biology, The John Paul II Catholic University of Lublin, Konstantynów Street 1I, 20-708 Lublin, Poland; 2 Department of Biomedicine and Environmental Research, The John Paul II Catholic University of Lublin, Konstantynów Street 1J, 20-708 Lublin, Poland; ewa.sajnaga@kul.pl; 3 Department of Industrial and Environmental Microbiology, Institute of Biological Sciences, Faculty of Biology and Biotechnology, Maria Curie-Skłodowska University, Akademicka Street 19, 20-033 Lublin, Poland; ewa.ozimek@mail.umcs.pl; 4 Department of Clinical Pharmacy and Pharmaceutical Care, Medical University of Lublin, Chodźki Street 1, 20-093 Lublin, Poland; anna.serefko@umlub.pl; 5 Department of Science, University “G. d’Annunzio” of Chieti-Pescara, Via dei Vestini 31, 66100 Chieti, Italy; m.locatelli@unich.it

**Keywords:** probiotics, phytochemicals, phytoprobiotic, antimicrobial resistance, antimicrobial synergy, gut microbiota, synbiotics, plant bioactive compounds, microbiome modulation, phage therapy

## Abstract

**Background/Objectives:** Antimicrobial resistance (AMR), microbiota disruption, and chronic inflammation have intensified the search for alternative and complementary antimicrobial strategies. Probiotics and plant-derived bioactive compounds (phytochemicals) are increasingly being investigated as microbiota-supporting, immunomodulatory, and antimicrobial agents. This review synthesizes the current evidence on probiotic–phytochemical interactions, with particular emphasis on mechanisms relevant to antimicrobial synergy, gut barrier reinforcement, microbiota modulation, and translational development. **Methods:** A narrative literature review with a structured search strategy was conducted using major scientific databases, including PubMed, Scopus, EBSCO, Google Scholar, SpringerLink, Wiley Online Library, and Taylor & Francis, and open repositories. Publications from January 2016 to April 2026 were considered, with an emphasis on experimental, preclinical, clinical, and mechanistic studies addressing the combined use of probiotics, postbiotics, plant extracts, or defined phytochemicals. **Results:** Available evidence indicates that selected probiotic–phytochemical combinations may enhance antimicrobial activity through complementary mechanisms, including pathogen membrane destabilization, inhibition of adhesion and biofilm formation, quorum-sensing interference, stimulation of probiotic viability and metabolite production, and biotransformation of phytochemicals into more active derivatives. These interactions may also support epithelial barrier integrity and immune regulation. However, the evidence remains heterogeneous and is strongly influenced by probiotic strain identity, phytochemical composition, dose, formulation, and the experimental model. Most studies are still limited to in vitro or animal models, and clinical validation remains scarce. **Conclusions:** Probiotic–phytochemical combinations represent a promising but insufficiently standardized strategy for antimicrobial and microbiota-targeted interventions. Future progress requires chemically characterized plant preparations, strain-level probiotic selection, harmonized synergy assays, advanced delivery systems, and well-designed clinical trials.

## 1. Introduction

Antimicrobial resistance (AMR), primarily driven by the overuse and misuse of antibiotics, is a significant global health threat. It is closely linked to increased morbidity, mortality, economic burden, disruption of the gut microbiota, and weakened host immunity. As frontline antibiotics lose their effectiveness, innovative and ecologically sustainable antimicrobial strategies are urgently needed [1,2,3].

In recent years, probiotics have gained considerable attention as potential adjuncts and alternatives to conventional antimicrobial therapies. Probiotics are live microorganisms that, when consumed in adequate amounts, confer health benefits to the host [4]. Classical lactic acid bacteria (LAB), including *Lactobacillus sensu lato* strains, exert broad antagonistic effects through competitive exclusion, immune modulation, biofilm interference, and production of antimicrobial metabolites such as organic acids, bacteriocins, and hydrogen peroxide. These antagonistic effects include the occupation of adhesion sites on mucus and epithelial surfaces, competition for fermentable carbohydrates and amino acids, and strain-dependent modulation of epithelial and dendritic cell (DC) responses [5,6,7]. These microbial products are relevant to the concept of postbiotics, which are defined as preparations of inanimate microorganisms and/or their components that confer health benefits to the host. In this context, probiotic-derived metabolites (e.g., organic acids and bacteriocins) released by live bacteria or after bacterial lysis contribute to postbiotic-mediated antimicrobial and immunomodulatory effects [8]. Simultaneously, non-LAB probiotics, such as *Bifidobacterium* spp., contribute to carbohydrate fermentation and acetate production [9,10], *Bacillus* spp. offer enhanced technological and gastrointestinal stability owing to spore formation [11,12], and *Escherichia coli* Nissle 1917 provides a well-characterized non-LAB probiotic model with distinct colonization and immunomodulatory properties [13]. These properties make them attractive candidates for clinical and biotechnological applications.

Plant-derived bioactive compounds have also gained attention because their antimicrobial, antioxidant, and immunomodulatory properties have been documented for centuries in traditional medicines. Importantly, compounds that act as antioxidants in mammalian tissues may exert pro-oxidant or redox-disruptive effects in bacterial cells, depending on their concentration, redox state, and microbial physiology [14,15]. Phytochemicals such as polyphenols, flavonoids, terpenoids, and alkaloids may inhibit pathogens through membrane disruption, virulence and quorum-sensing (QS) interference, efflux pump inhibition, and prevention or disruption of biofilm formation, among other mechanisms [16,17,18]. Owing to these antimicrobial properties, many phytochemicals have been investigated as antimicrobial-supportive agents and as natural preservatives [18]. Their potential is particularly relevant in addressing the growing challenges of antimicrobial resistance and drug-resistant pathogens [19]. Importantly, many plant extracts exhibit prebiotic properties, selectively promoting the growth and metabolic activity of beneficial probiotic strains [20,21,22]. Prebiotics are substrates selectively utilized by host microorganisms, conferring a health benefit [4]. Synbiotics are mixtures comprising live microorganisms and substrate(s) selectively utilized by host microorganisms that confer a health benefit [4,23].

Despite these advances, most studies have examined probiotics and plant extracts in isolation. Emerging evidence suggests that selected probiotic–phytochemical combinations may exert complementary or synergistic multi-target effects, including enhanced antimicrobial activity, biofilm disruption, modulation of redox and inflammatory pathways, and support for intestinal barrier integrity. In some experimental settings, such combinations may also contribute to antibiotic-sparing strategies by lowering the concentration of conventional antimicrobials required for pathogen inhibition; however, this effect requires confirmation using appropriate quantitative combination assays in future studies. This integrated strategy aligns with modern ecological and systems biology perspectives, in which the gut microbiota and its metabolic networks are viewed as dynamic ecosystems that can be modulated using complementary, bioactive agents [24,25]. In 2023, Wishna-Kadawarage et al. proposed the term “prophybiotic” to describe a formulation that combines a probiotic microorganism with a plant extract in one preparation [26]. Related terminology has also appeared in the literature; for example, Savelyeva et al. used the term “phyto probiotic complex” for a natural preparation combining probiotic and plant-derived components [27]. In this review, we use the term “phytoprobiotic” as an operational descriptor for combined formulations or intervention strategies that integrate probiotic microorganisms with plant-derived bioactives/extracts to achieve complementary effects on the gut microbiota, antimicrobial activity, and host-related outcomes. Because no standardized definition of “phytoprobiotic” currently exists, the term is used here descriptively and should not be interpreted as a formally established regulatory or consensus category.

Although several reviews have addressed probiotics, plant bioactives, or synbiotic-like approaches, fewer studies have specifically integrated evidence on probiotic–plant bioactive combinations while clearly distinguishing quantitatively confirmed synergy from additive or complementary interactions. Therefore, this review provides a focused mechanistic synthesis of the current knowledge on probiotic–phytochemical interactions, with particular attention to documented synergy, methodological limitations, and translational relevance for gut health, and their supportive role in gut-health-oriented and antimicrobial-supportive strategies. We discuss the mechanistic foundations of probiotic action, chemical diversity, and antimicrobial activity of plant bioactives and the emerging evidence for their combined effects. By integrating mechanistic, ecological, and translational perspectives, this review proposes a conceptual framework for evaluating probiotic–plant bioactive combinations as microbiota-targeted strategies in the post-antibiotic era.

## 2. Methodology of the Review—Narrative Literature Review with a Structured Search Strategy

The literature used in this narrative review with structured search was collected over a period of three months using databases and platforms available through the library system of the John Paul II Catholic University of Lublin (KUL), including Scopus, PubMed, EBSCO, Google Scholar, De Gruyter, Springer Nature, Oxford University Press—Medicine Collection, SAGE Premier, Science, SpringerLink, Taylor & Francis, Wiley Online Library, and open repositories. The search covered publications from January 2016 to April 2026. Keywords were used individually and in Boolean combinations, including: “probiotics”, “postbiotics”, “metabolites”, “synbiotic”, “randomized controlled trials”, “meta-analyses”, “phytochemicals”, “plant extract”, “in vitro”, and “antimicrobial action”, for example: (probiotic* OR *Lactobacillus* OR *Bifidobacterium* OR *Bacillus*) AND (phytochemical* OR plant extract* OR polyphenol* OR flavonoid*) AND (synerg* OR FICI OR checkerboard OR time-kill OR co-culture) AND (gut OR microbiota OR inflammation OR chronic disease). The inclusion criteria primarily focused on clinical trials (RCTs), in vitro studies, animal studies, meta-analyses, and mechanistic reviews of the concurrent use of probiotics/postbiotics and plant-derived substances for gut health and chronic disease prevention. Non-peer-reviewed articles, case reports, and publications not available in English were excluded.

Additionally, operational definitions were used in this review:

- Synergy: FICI ≤ 0.5, or ≥2 log10 CFU/mL reduction versus the best single agent in time-kill assays.
- Additive effect: 0.5 < FICI ≤ 1.0;
- Indifference: 1.0 < FICI ≤ 4.0;
- Antagonism: FICI > 4.0.
- Complementary interaction: mechanistically consistent interaction without a formal synergy test.

## 3. Mechanisms of the Antibacterial Action of Probiotic Microorganisms

**Figure 1:**
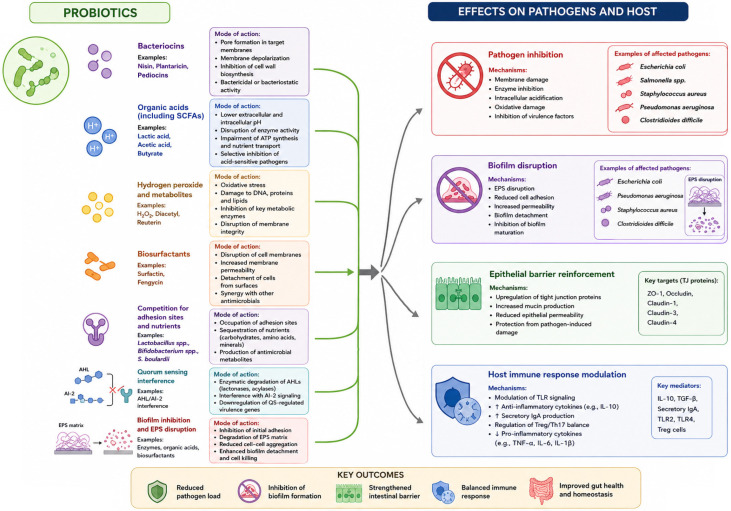
Overview of the principal mechanisms underlying the antimicrobial and host-modulatory activity of probiotic microorganisms. These mechanisms include the production of organic acids and short-chain fatty acids (SCFAs), secretion of bacteriocins and antimicrobial metabolites, biosurfactant-mediated effects, competition for nutrients and adhesion sites, quorum-sensing interference, biofilm inhibition, extracellular polymeric substance (EPS) disruption, epithelial barrier reinforcement, and modulation of host immune responses. Together, these activities contribute to pathogen inhibition, biofilm disruption, intestinal barrier protection, immune homeostasis, and improved overall gut health. Abbreviations: AHL, acyl-homoserine lactone; AI-2, autoinducer-2; EPS, extracellular polymeric substances; IgA, immunoglobulin A; IL, interleukin; SCFA, short-chain fatty acid; TJ, tight junction; TLR, Toll-like receptor; Treg, regulatory T cell; Th17, T-helper 17 cell.

Probiotic microorganisms employ a wide range of antibacterial strategies that work together to suppress harmful bacteria, influence the balance of microbial communities, and enhance the host immune system. These mechanisms are multifaceted, often overlapping, and involve both direct antagonism toward pathogens and indirect modulation of host–microbe interactions [28]. Understanding these mechanistic layers is essential for elucidating the antimicrobial and host-related functions of probiotics and provides a mechanistic basis for interpreting their potential interactions (Figure 1).

### 3.1. Production of Organic Acids and Environmental Acidification

LAB and *Bifidobacterium* species produce lactic, acetic, and other short-chain fatty acids (SCFAs) that lower gut pH, creating unfavorable conditions for acid-sensitive pathogens [28]. Quantitative analyses demonstrated strain-dependent differences in secretion of organic acids, including SCFAs. For example, the *Lacticaseibacillus rhamnosus* strain 484 isolated from human breast milk produced 14.11 mg/mL (≈14.1 g/L) of L-lactic acid after 24 h of cultivation, exceeding the production observed for the reference *L. rhamnosus* GG strain (12.89 mg/mL), highlighting the inter-strain variability in acidification capacity [29]. Interestingly, *L. rhamnosus* GG predominantly produces L-lactate and no D-lactate was detected after 48 h of cultivation, with L-lactate concentrations exceeding 100 mmol/L, supporting its favorable metabolic profile for probiotic applications [29,30]. Furthermore, *Lactiplantibacillus plantarum* AC11S produced approximately 18 g/L of lactic acid during lactose fermentation at pH 6.5 and 30 °C, illustrating the high acidification potential characteristic of many lactic acid bacteria [31]. In vitro HPLC-MS/MS measurements showed that *Limosilactobacillus reuteri* DSM 17938 and *Bacillus clausii* T produced the highest amounts of acetate (644.3 ± 7.2 and 602.0 ± 54.2 ng/mL, respectively), whereas *B. clausii* strains also secreted detectable levels of propionate (up to 1.21 ± 0.38 ng/mL) and butyrate (2.70–3.04 ng/mL), highlighting the metabolic diversity among probiotic microorganisms [32]. Not all probiotic-mediated acidification relies predominantly on lactate production. For example, the probiotic strain *Clostridium butyricum* MIYAIRI 588, which belongs to lactate-utilizing bacteria (LUB), contributes to intestinal acidification mainly through butyrate production, thereby supporting colonization resistance and illustrating metabolic complementarity among probiotic species [33]. Such diversity may be beneficial, as excessive D-lactate accumulation by certain lactic acid-producing bacteria has been associated with D-lactic acidosis in susceptible individuals, particularly in patients with short bowel syndrome [33,34]. Similarly, another LUB strain, *C. butyricum* TO-A, efficiently converted lactate and acetate into butyrate, reaching concentrations of 5.05 ± 0.82 mM in the presence of both substrates and 7.64 ± 0.67 mM in the presence of D-lactate and acetate, demonstrating the strain’s capacity to act as an efficient butyrate supplier and emphasizing the metabolic diversity among probiotic species [35].

Beyond simple acidification, undissociated organic acids diffuse across bacterial membranes, dissociate in the neutral cytoplasm, and disrupt intracellular pH homeostasis, thereby impairing key metabolic pathways [36]. This acidification is mainly local and depends on fermentation intensity, buffering capacity, and intestinal region [28,36,37]. Acid-sensitive pathogens include *Clostridioides difficile*, pathogenic *E. coli*, *Listeria monocytogenes*, and *Salmonella* [28,36]. Intracellular acidification disrupts enzyme activity, ATP generation, amino acid transport, and proton-gradient-dependent metabolism [36]. SCFAs, particularly butyrate, support tight junction (TJ) proteins, including occludin, claudin-1, and ZO-1, inhibit NF-κB partly through histone deacetylase (HDAC)-dependent mechanisms, and modulate cytokines such as IL-6, IL-8, TNF-α, and IL-10 [37,38].

### 3.2. Bacteriocin Production and Antimicrobial Peptides

Many probiotic and food-associated bacteria produce antimicrobial peptides (AMPs), known as bacteriocins [39]. Representative examples include nisin, produced mainly by *Lactococcus lactis* [40], plantaricin, produced by *L. plantarum* strains [41], and pediocins, produced by *Pediococcus* spp. (e.g., pediocin PA-1 produced by *P. acidilactici* strains) [42]. These small, cationic, amphiphilic peptides primarily disrupt target cell membranes, induce pore formation, inhibit cell wall or envelope synthesis; in some cases, indirect effects on QS-regulated phenotypes have also been reported [43,44,45,46]. For example, bacteriocin Abp118 produced by *Ligilactobacillus salivarius* UCC118 has been reported to exhibit anti-listerial activity in animal models, whereas enterocin CRL35 produced by *Enterococcus mundtii* RL35 showed activity against *Listeria* spp. and selected viral pathogens, highlighting the broad antimicrobial spectrum of bacteriocins [47]. Similarly, pediocin PA-1 displayed potent anti-listerial activity, with a minimum inhibitory concentration of 45 nM against *L. monocytogenes*, and effectively suppressed pathogen growth in a model of the human gut environment [42]. Furthermore, the two-peptide bacteriocin plantaricin NC8 αβ permeabilized bacterial membranes and exhibited potent activity against *Staphylococcus aureus* and *Staphylococcus epidermidis*, illustrating the membrane-targeting mode of action characteristic of many bacteriocins [48]. Given the structural diversity of bacteriocins, only representative mechanisms and antimicrobial targets are discussed here and summarized in Figure 1.

In addition to bacteriocins, some *Bacillus* strains also produce non-ribosomal lipopeptides, including surfactin and fengycin, which have biosurfactant and biofilm-disrupting properties. These lipopeptides are synthesized by non-ribosomal peptide synthetases rather than ribosomal translation. Surfactin and fengycin have been reported to affect pathogens such as *Campylobacter jejuni*, *S. aureus*, and selected Gram-negative biofilm-forming bacteria [49,50,51]. These lipopeptides destabilize microbial membranes through pore formation and surface activity, contributing to activity against both planktonic pathogens and biofilm-associated cells [49].

### 3.3. Competition for Nutrients and Adhesion Sites

Probiotics inhibit pathogen colonization through competitive exclusion, which involves both the occupation of ecological niches and competition for host-associated binding sites and nutrients. Many probiotic strains adhere to intestinal mucus and mucins, thereby limiting pathogen access to the mucosal surface and supporting colonization resistance [52,53]. Adhesion may involve strain-specific surface molecules, including S-layer proteins, mucus-binding proteins, exopolysaccharides, and other cell envelope structures that interact with mucin and epithelial receptors. In some models, interactions with epithelial adhesion-related molecules, such as E-cadherin- or integrin-associated pathways, have been proposed [52]. For example, the specific adhesion factors of *L. plantarum* strains contribute to host–microbe interactions and pathogen exclusion. The collagen-binding protein of *L. plantarum* Lp91 exhibited anti-adhesive activity against enteric pathogens, thereby promoting competitive exclusion and colonization resistance, whereas adhesion-related proteins identified in *L. plantarum* HEAL9 and 299v have been implicated in persistence within the intestinal mucus layer [54]. In addition, probiotic surface-associated microbial patterns may be recognized by host pattern recognition receptors, including Toll-like receptors (TLRs), although these interactions are highly strain- and context-dependent and should not be generalized across all probiotic taxa [52,53].

Probiotics also suppress pathogen proliferation by utilizing key nutrients, including carbohydrates and amino acids, thereby reducing the resources available for competing microorganisms [53]. For example, the probiotic strain *E. coli* Nissle 1917 contributed to colonization resistance by competing with enterohemorrhagic *E. coli* (EHEC) O157 for mucus-derived monosaccharides, including arabinose, galactose, N-acetylglucosamine, mannose, and ribose, thereby occupying the same nutrient niche and limiting pathogen colonization [55].

### 3.4. Modulation of Host Immune Responses

The effects of probiotics on host immunity are highly strain-specific and depend on microbial surface structures, secreted metabolites, host cell type, and inflammatory context. Selected probiotic strains interact with intestinal epithelial cells, DCs, macrophages, and other mucosal immune cells through microbe-associated molecular patterns (MAMPs) recognized by pattern recognition receptors, including TLRs [56,57]. However, these interactions should not be generalized across all probiotic taxa because different strains may induce distinct cytokine profiles and immune outcomes [58,59].

Some probiotic strains promote mucosal immune readiness by increasing secretory IgA production, modulating DC maturation, and supporting balanced innate immune responses [60,61,62]. For example, *Lactobacillus delbrueckii* subsp. *lactis* PTCC 174 and *L. rhamnosus* ATCC 9595 may enhance anti-inflammatory mediators, such as IL-10, while modulating pro-inflammatory cytokines, including IL-12 and TNF-α, although the direction and magnitude of these effects depend strongly on the strain, dose, and experimental model [59]. Specific probiotic strains have been shown to support epithelial barrier integrity by modulating TJ-associated protein expression and reducing epithelial barrier dysfunction [63,64,65,66]. For example, *L. rhamnosus* GG and *Lacticaseibacillus paracasei* IMPC2.1 mitigate lipopolysaccharide (LPS)-induced epithelial barrier dysfunction in Caco-2 cells by regulating TJ protein expression and autophagy-related pathways [63]. A multi-strain formulation containing *L. rhamnosus* LR32, *Bifidobacterium animalis* subsp. *lactis* BL04, and *Bifidobacterium longum* BB536 also improved epithelial barrier function by modulating tight and adherens junction proteins [64]. In addition, Wang et al. demonstrated that even closely related strains of the same species, such as *L. plantarum* P1 (CCFM200) and P2 (CCFM8610), differed in their ability to restore TJ integrity in dextran sulfate sodium (DSS)-induced colitis. Similar variability was observed for *Lacticaseibacillus casei* C1 (CCFM9) and *Limosilactobacillus fermentum* F1 (CCFM437), highlighting that barrier-protective effects cannot be inferred solely from species identity [65].

### 3.5. Quorum Sensing Interference (Quorum Quenching)

Several probiotic strains have been shown to disrupt QS, a critical communication system that controls bacterial virulence, toxin synthesis, and biofilm development. By breaking down or capturing autoinducers (AI), such as acylated homoserine lactones (AHLs) and AI-2, probiotics can diminish pathogen virulence without applying the selective pressure typically linked to antibiotic use [67]. Mechanistically, quorum-quenching may involve enzymatic degradation of AHLs by lactonases or acylases, interference with AI-2 signaling, adsorption or sequestration of signaling molecules, and suppression of QS-regulated virulence gene expression [67,68]. For example, *L. reuteri* LR21-derived reuterin suppressed *Clostridium perfringens* QS- and toxin-related genes (*agrB*, *luxS*, *cpa*, and *pfo*), whereas *L. reuteri* RC-14-derived cyclo-dipeptides inhibited *Staphylococcus aureus* virulence regulation by targeting the *agr*, *tst*, and SaeRS systems. In Gram-negative models, *Levilactobacillus brevis* 3M004 and *Bacillus paralicheniformis* ZP1 interfered with *Pseudomonas aeruginosa* QS through AHL degradation or lactonase-mediated AHL hydrolysis, leading to reduced biofilm formation and repression of QS-controlled genes, including *lasA*, *lasB*, and *phzAB* [69].

These observations indicate that quorum-quenching is not restricted to a single mechanism but may involve the enzymatic degradation of signaling molecules and indirect modulation of virulence-associated pathways. Importantly, interference with QS does not necessarily inhibit bacterial growth directly but rather attenuates pathogen fitness and pathogenicity, potentially reducing the selective pressure for resistance development. Such antivirulence effects have been reported against clinically relevant pathogens, including *P. aeruginosa*, *S. aureus*, and *C. perfringens*, highlighting quorum-quenching as a complementary mechanism of probiotic-mediated pathogen control [67,68,69].

### 3.6. Inhibition and Disruption of Biofilms

Biofilms confer antibiotic tolerance and persistence to bacteria [70]. Probiotics counteract biofilm formation through multiple mechanisms, including the secretion of biosurfactants, competitive adherence, production of SCFAs that weaken extracellular polymeric substances (EPS), and enzymatic degradation of biofilm matrices [71,72,73]. For example, Zhang et al. showed that cell-free supernatants from *Amylolactobacillus animalis* LMEM6, *L. plantarum* LMEM7, *Lactobacillus acidophilus* LMEM8, *L. rhamnosus* LMEM9, and *L. fermentum* MTCC 9748 inhibited biofilm formation by MDR *Klebsiella pneumoniae* in a chemostat system, partly by reducing intracellular c-di-GMP levels and modulating biofilm-associated genes, including *yfiN* and *mrkJ* [72]. *Bacillus* spp. produce potent biosurfactants, such as surfactin, which decrease surface tension, interfere with biofilm stability, and promote biofilm dispersion. Surfactin has been reported to affect biofilms formed by pathogens, including *P. aeruginosa* and *S. aureus* [74]. Recent studies further support the anti-biofilm potential of probiotics. For example, *L. reuteri* DSM 17938 and ATCC PTA 5289 reduced the biomass of dual-species biofilms formed by clinical isolates of *Streptococcus sobrinus* and *Candida albicans* by approximately 35%. This effect was accompanied by decreased microbial counts and downregulation of virulence-associated genes, such as *gtfI* and *HWP1*, indicating that probiotic-mediated biofilm inhibition may involve both structural disruption and attenuation of virulence-associated pathways [75].

### 3.7. Coaggregation and Pathogen Entrapment

Coaggregation refers to the ability of probiotic cells to physically bind to pathogens. This mechanism entraps pathogens, limiting their motility, aggregation, and adherence to host tissues. Coaggregation facilitates pathogen removal via peristalsis or mucosal turnover [76,77,78]. For example, *L. rhamnosus* 1B06 and *L. paracasei* 8A12 coaggregated with oral pathogens such as *Fusobacterium nucleatum*, *Porphyromonas gingivalis*, and *Prevotella*, aiding in pathogen displacement [79]. Coaggregation is highly strain-dependent and may involve surface-associated proteins, lipoteichoic acids, S-layer proteins, and exopolysaccharides that facilitate cell-to-cell interactions. In addition to limiting pathogen adhesion, coaggregates may create physical barriers that restrict nutrient access and enhance pathogen clearance from mucosal surfaces [76,77,78,79]. Further evidence highlights the strain-specific nature of coaggregation. For example, *L. acidophilus* Lb2 and *L. fermentum* Lb8 exhibited strong coaggregation with enteric pathogens, including *E. coli*, *Shigella sonnei*, and *Providencia alcalifaciens*, reaching coaggregation rates of up to 100% after 24 h of incubation. These interactions facilitate pathogen entrapment and contribute to colonization resistance [80]. A multi-strain probiotic formulation containing *L. plantarum* PBS067, *L. rhamnosus* LRH020, and *B. animalis* subsp. *lactis* BL050 showed rapid coaggregation with *Gardnerella vaginalis*, *E. coli*, and *C. albicans*, with visible aggregates forming within 15 min. Scanning electron microscopy further demonstrated close physical interactions between probiotics and pathogens, including the partial surrounding of *C. albicans* cells by *L. rhamnosus* LRH020 and the embedding of fungal cells within the probiotic biofilm, supporting the role of coaggregation in pathogen sequestration and colonization resistance [81].

### 3.8. Metabolic Crosstalk and Production of Antimicrobial Metabolites

In addition to classical AMPs and acids, probiotics produce a spectrum of metabolites such as acetaldehyde, diacetyl, hydrogen peroxide, and reuterin that inhibit a wide array of pathogens [82,83,84]. Acetaldehyde and diacetyl, volatile antimicrobial metabolites, contribute to the preservation of fermented foods [85]. In some cases, acetaldehyde can be further metabolized into ethanol by certain strains such as *L. reuteri* DSM 20016 [86]. Hydrogen peroxide-producing lactobacilli contribute to pathogen control by generating reactive oxygen species (ROS), which disrupt bacterial cell envelopes and interfere with cellular metabolism [87,88]. For example, *L. reuteri* DSM 17938 produces reuterin through glycerol metabolism, and this compound exhibits broad-spectrum activity against Gram-positive and Gram-negative bacteria, fungi, and protozoa. Reuterin acts primarily by inducing oxidative stress and disrupting thiol-dependent metabolic pathways, thereby inhibiting microbial growth [84].

Recent studies have further illustrated the metabolic diversity underlying probiotic antimicrobial activity. For example, the *pdu-cbi-cob-hem* gene cluster responsible for reuterin biosynthesis was identified in *L. reuteri* E81, and the production of reuterin and related 3-hydroxypropionaldehyde (3-HPA)-derived metabolites was confirmed by NMR analyses. Additionally, reuterin-producing *L. reuteri* E81 reduced *E. coli* counts from 6.4 log CFU/g to undetectable levels after 30 days, supporting reuterin-mediated bioprotection through membrane disruption and oxidative stress [89]. The antimicrobial potency of reuterin varies depending on the producing strain and target microorganism. Reuterin produced by *L. reuteri* 12002 exhibited MIC values of 4 AU/mL against *E. coli* and 8 AU/mL against *L. monocytogenes* [90], whereas inhibition of *E. coli* DH5α and *S. aureus* has been reported at approximately 0.9 mM and 1.5 mM, respectively [91]. These observations indicate a concentration-dependent activity against both Gram-negative and Gram-positive bacteria. These findings highlight that probiotic-mediated pathogen inhibition extends beyond organic acid production and involves structurally diverse antimicrobial metabolites [89]. Although reuterin exhibits concentration- and cell type-dependent cytotoxicity, studies on mammalian cell models indicate that it does not exert significant toxicity at concentrations associated with antimicrobial activity, supporting its potential for food and therapeutic applications [92,93].

## 4. Antibacterial and Immunomodulatory Activity of Plant Extracts

Plant-derived extracts inhibit bacterial pathogens through several molecular mechanisms. These mechanisms are largely influenced by bioactive compounds, including alkaloids, essential oils, flavonoids, polyphenols, and tannins, which target bacterial structures and functions such as membranes, enzymes, energy metabolism, and communication systems [94]. Many plant-derived compounds described as antioxidants in host tissues may exert context-dependent pro-oxidant or redox-disruptive effects in bacterial cells. Therefore, their antioxidant activity in mammalian systems does not conflict with the generation of antibacterial ROS; instead, the ultimate effect is determined by factors such as the type of target cell, concentration of the compound, availability of metals, and microbial redox state [94,95].

In addition to their antibacterial properties, plant-derived extracts exhibit antiviral, antioxidant, and immunomodulatory properties [96,97,98,99]. Their extensive biochemical versatility allows them to simultaneously target multiple cellular pathways, presenting promising strategies for combating multidrug-resistant (MDR) pathogens [100,101], including *E. coli*, *K. pneumoniae*, and *S. aureus* [102,103]. In contrast to conventional antibiotics, many phytochemicals operate through multisite mechanisms, reducing the risk of resistance development and complementing the actions of probiotics [104,105,106,107,108].

### 4.1. Direct Antibacterial Mechanisms of Plant Phytochemicals

Plant-derived bioactive compounds can affect bacterial survival, virulence, and adaptation through multiple complementary mechanisms. Rather than acting on a single cellular target, many phytochemicals simultaneously influence several bacterial processes, including disruption of membrane integrity, interference with metabolic activity, inhibition of QS, and prevention of biofilm formation [94,109]. The examples discussed below include cinnamon-derived cinnamaldehyde [110,111,112], green tea epigallocatechin gallate (EGCG) [113,114], oregano-derived carvacrol and thymol [115,116], turmeric-derived curcumin [117,118], grape seed proanthocyanidins [119,120], and pomegranate ellagitannins [121,122], which have been investigated for their antibacterial, anti-biofilm, and antivirulence properties [94]. The following sections summarize the principal mechanisms through which phytochemicals may contribute to pathogen control and potentially complement probiotic-derived antimicrobial activity.

#### 4.1.1. Disruption of Cell Membrane Integrity

Many polyphenols, including catechins and gallic acid derivatives, as well as essential oil components, such as thymol and carvacrol, interact with bacterial lipid bilayers and membrane proteins, increasing membrane permeability, altering fluidity, and impairing nutrient transport [94,123,124]. These effects may lead to intracellular ion leakage, depolarization, metabolic collapse, and bacterial cell lysis. For example, terpene mixtures containing β-myrcene and α-humulene have been shown to increase bilayer fluidity and disrupt membrane structure [125]. Moreover, selected polyphenols may exert context-dependent pro-oxidant effects in bacterial cells by promoting ROS accumulation, ATP leakage, and membrane rupture, despite their antioxidant properties in host tissues [95].

The antibacterial effect of membrane disruption is not limited to structural damage. Increased membrane permeability may result in the loss of the proton motive force, disruption of ATP synthesis, impaired nutrient uptake, and altered activity of membrane-associated enzymes and transport systems [94,126]. These effects compromise bacterial energy homeostasis and reduce the ability of pathogens to maintain their cellular integrity under environmental stress [127]. Importantly, susceptibility to membrane-active phytochemicals is often species dependent. Gram-positive bacteria are generally more vulnerable because their cytoplasmic membrane is protected only by a thick peptidoglycan layer, whereas Gram-negative bacteria possess an additional outer membrane that can partially restrict the penetration of hydrophobic compounds [94]. Consequently, the antibacterial activity of membrane-disrupting phytochemicals depends not only on the compound structure and concentration but also on the bacterial cell envelope architecture and physiological state [126,127].

Membrane perturbation may also enhance the activity of probiotic-derived antimicrobial metabolites, including organic acids, bacteriocins, and biosurfactants [126,128]. Increased permeability facilitates the diffusion of these compounds into bacterial cells and may lower the concentration required to achieve growth inhibition [127,128]. This mechanism provides a plausible explanation for the many reported additive or synergistic interactions between phytochemicals and probiotic-derived antimicrobials, particularly in studies involving biofilm-forming and MDR pathogens [117,129,130].

#### 4.1.2. Inhibition of Bacterial Enzymes and Metabolic Pathways

Plant-derived polyphenols and other bioactive compounds can inhibit essential bacterial metabolic pathways, including protein biosynthesis, ATP production, and DNA replication and cell division [94,109]. Phenolic acids, flavonoids, alkaloids, and terpenoids may act as enzyme inhibitors by interacting with catalytic sites, altering protein conformation, and interfering with cofactor-dependent reactions [126]. Several phytochemicals have been reported to interfere with DNA gyrase, topoisomerases, and ATP-binding enzymes, thereby impairing bacterial replication, transcription, and metabolism [109,126].

A well-described example is curcumin, which inhibits FtsZ, a bacterial cytoskeletal protein essential for Z-ring formation and cytokinesis [131,132]. Interference with FtsZ polymerization disrupts septum formation, leading to defective cell division, filamentation, and growth arrest in susceptible bacteria [131]. However, curcumin activity is strongly strain- and model-dependent, and its limited solubility and bioavailability remain important constraints for its translational applications [118,133].

Phytochemicals may also disrupt bacterial energy metabolism by reducing ATP generation, inhibiting membrane-associated respiratory enzymes, and altering proton-dependent transport systems [94,126]. Essential oil constituents, such as thymol and carvacrol, can affect ATP synthesis and membrane-associated metabolic processes, contributing to growth inhibition and reduced stress tolerance in pathogens [134,135]. In addition, phenolic compounds may inhibit bacterial efflux pumps, increasing the intracellular accumulation of antimicrobial agents and reducing one of the major mechanisms of multidrug resistance [109,126].

These enzyme- and metabolism-targeting effects are particularly relevant for probiotic–phytochemical combinations because metabolic weakening of pathogens may increase their susceptibility to organic acids, bacteriocins, hydrogen peroxide, and biosurfactants produced by probiotics [128]. Probiotic-derived metabolites are increasingly recognized as important contributors to pathogen inhibition and may complement phytochemical-induced metabolic disruption [136]. Inhibition of bacterial enzymes and metabolic pathways represents not only a direct antibacterial action of plant-derived bioactives but also a potential mechanism for enhancing the effectiveness of probiotic-derived antimicrobial metabolites. This hypothesis can be evaluated using appropriate combination assays to determine whether additive or synergistic interactions occur between the two.

#### 4.1.3. Suppression of Virulence Factors and Quorum Sensing

Many plant-derived bioactive compounds suppress bacterial virulence gene expression by interfering with QS, a cell density-dependent communication system that coordinates toxin production, motility, adhesion, and biofilm maturation [112,126]. QS networks are mediated by signaling molecules such as AHLs, AI-2, and species-specific peptide signals, which regulate collective pathogenic behavior rather than bacterial growth [68]. Therefore, plant-derived QS inhibitors may attenuate virulence without necessarily exerting direct bactericidal effects [110,137].

Several phytochemicals have been shown to interfere with QS-regulatory processes. For example, naringenin can inhibit virulence-associated signaling and biofilm-related behaviors in bacterial models, including *Pectobacterium brasiliense* and *P. aeruginosa* [138,139]. Phenolic volatiles and other plant-derived compounds may reduce bacterial virulence by interfering with LuxI/LuxR-type regulatory systems and related QS proteins [140]. Flavonoids such as quercetin may also interact with QS-related proteins, supporting their potential role in disrupting bacterial signaling pathways [141]. In addition, cinnamaldehyde, eugenol, quercetin, and related phenolics have been reported to reduce QS-dependent phenotypes, including swarming motility, toxin production, adhesion, and biofilm formation [110,111,112]. Cannabigerol (CBG) and cannabigerolic acid (CBGA) have also been investigated mainly as plant-derived anti-biofilm and antivirulence compounds; however, their direct QS-related effects require further confirmation across bacterial species [112,126].

This virulence-attenuation mechanism is particularly relevant because QS inhibition does not necessarily eliminate bacteria directly but weakens the coordinated pathogenic behaviors that facilitate infection persistence and biofilm tolerance [110,137]. By reducing toxin secretion, motility, adhesion, and biofilm maturation, phytochemicals may render pathogens more susceptible to host defenses and probiotic-derived antimicrobials, including organic acids, bacteriocins, hydrogen peroxide, and biosurfactants [110,128]. In this context, QS suppression may provide a mechanistic bridge between plant bioactives and probiotic antimicrobial activity, as probiotics may also interfere with microbial communication through AI degradation, metabolic competition, and biofilm displacement [67,68].

#### 4.1.4. Inhibition of Biofilm Formation and Degradation of Exopolysaccharides

Plant secondary metabolites, particularly tannins, stilbenes, and flavonoids, can interfere with multiple stages of biofilm development, including initial bacterial adhesion, microcolony formation, maturation, and EPS matrix maintenance [117,129]. Biofilms represent one of the most important bacterial survival strategies because they provide protection against environmental stressors, host immune defense, and antimicrobial agents [130]. Consequently, biofilm-associated bacteria frequently exhibit substantially greater antibiotic tolerance than their planktonic counterparts [129,130].

Several plant-derived compounds have been shown to disrupt biofilm architecture through complementary mechanisms. Tannins may interact with extracellular proteins and polysaccharides, thereby reducing matrix stability and bacterial adhesion [117,126]. Hydrolyzable tannins can denature surface-associated proteins involved in biofilm formation and maintenance, thereby weakening the structural integrity of mature biofilms [118,126]. Stilbenes and flavonoids may further impair biofilm development by altering bacterial signaling pathways and reducing the expression of adhesion-related genes [112,129]. In addition, several essential oil constituents, including thymol, carvacrol, and eugenol, have been shown to penetrate biofilm matrices, increase membrane permeability, and promote partial biofilm disruption in selected bacterial species [126].

The anti-biofilm activity of phytochemicals is closely linked to their ability to interfere with bacterial communication. Many plant-derived compounds inhibit the QS pathways that regulate EPS production, motility, surface attachment, and biofilm maturation [110,112]. By suppressing these regulatory networks, phytochemicals may prevent the establishment of mature biofilms and reduce the persistence of chronic infection [110,137]. Importantly, the inhibition of biofilm formation and disruption of established biofilms are distinct biological phenomena and should not be interpreted as interchangeable. Compounds that are effective against early-stage biofilm development may show limited activity against mature biofilm structures [129,130].

From the perspective of probiotic–phytochemical interactions, disrupting biofilms may enhance the activity of probiotic-derived antimicrobial metabolites, including organic acids, bacteriocins, hydrogen peroxide, and biosurfactants [128]. Reduced EPS density and increased matrix permeability may improve the diffusion of these compounds into deeper biofilm layers and enhance their antimicrobial effectiveness [117,130]. Furthermore, selected probiotic strains may compete with pathogens for adhesion sites and contribute to biofilm displacement, providing an additional mechanism through which phytochemicals and probiotics may act in a complementary manner [67,128].

However, the anti-biofilm activity of plant extracts remains highly dependent on the extract composition, concentration, bacterial species, and experimental conditions [126,129]. Therefore, claims regarding probiotic–phytochemical synergy in biofilm control should ideally be supported by quantitative biofilm assays, microscopic analyses, EPS measurements, and combination studies that demonstrate enhanced activity relative to the individual components [117,130].

### 4.2. Prebiotic and Microbiota-Modulating Properties of Plant Extracts

In addition to their antibacterial effects, many plant extracts act as selective prebiotic substrates that stimulate the growth and metabolic activity of probiotic bacteria. Complex carbohydrates, polyphenols, and glycosides escape upper gastrointestinal (GI) digestion and are metabolized by probiotic species into smaller bioactive molecules, including SCFAs (acetate, propionate, and butyrate), phenolic metabolites with higher bioactivity than native compounds, and anti-inflammatory catabolites that improve the host immune function [142,143,144,145]. For example, fermentation of guava leaf extract by *Lactobacillus s.l.* strains increased antioxidant and antiglycation activities owing to the synergistic effects between microbial metabolites and plant-derived compounds [146].

This metabolic interplay enhances probiotic colonization, promotes microbial eubiosis, and strengthens the host barrier function [147,148,149]. In synbiotic formulations, plant extracts and probiotic bacteria display reciprocal enhancement: probiotics biotransform phytochemicals into more active metabolites, whereas phytochemicals stimulate probiotic growth and metabolite production [150,151,152,153]. Interestingly, plant-derived prebiotic substrates can be metabolized by intestinal bacteria into SCFAs, including butyrate, thereby linking plant bioactives to gut barrier reinforcement, anti-inflammatory effects, and improved gut health [154,155]. Examples include inulin, fructooligosaccharides (FOS), galacto-oligosaccharides (GOS), resistant starch, pectins, arabinoxylans, and β-glucans, which can be metabolized by intestinal bacteria, including members of the genera *Bifidobacterium*, *Faecalibacterium*, *Lactobacillus*, and *Roseburia*, into SCFAs such as acetate, propionate, and butyrate [156,157]. This transformation may also enhance probiotic adhesion by reinforcing the gut barrier and mitigating inflammation [154,155]. For example, B-type lotus seedpod oligomeric procyanidins enhanced the adhesion of *Lacticaseibacillus rhamnosus* GG to IPEC-J2 intestinal epithelial cells while simultaneously reducing the adhesion of enterotoxigenic *E. coli* (ETEC), supporting competitive exclusion and intestinal colonization resistance [155].

### 4.3. Immunomodulatory Effects of Plant Compounds

Plant extracts influence both innate and adaptive immunity through several pathways, including the modulation of inflammatory signaling in immune cells, as shown for ginger-derived bioactive compounds [158]. The potential of plant extracts as natural immunomodulators has been demonstrated, with applications in managing inflammation, enhancing resistance to infections, and supporting overall immune health [159,160,161,162].

#### 4.3.1. Antioxidant and Anti-Inflammatory Modulation

Polyphenols scavenge ROS, inhibit NF-κB activation, and downregulate pro-inflammatory cytokines, including IL-1β, IL-8, and TNF-α, thereby mitigating pathogen-induced inflammation and supporting mucosal recovery [158,163,164,165,166]. Enhanced antioxidant activity may also contribute to the stabilization of epithelial TJ and maintenance of intestinal barrier integrity [119,167,168,169]. In addition to direct radical scavenging, several polyphenols regulate intracellular inflammatory signaling pathways. For example, catechins, quercetin, and resveratrol have been reported to modulate NF-κB- and the mitogen-activated protein kinase (MAPK)-related signaling and reduce the expression of pro-inflammatory mediators, including TNF-α, IL-1β, IL-6, and IL-8, depending on the compound, cell type, and type of inflammatory stimulus [158,170]. These effects may contribute to reduced inflammation-associated epithelial damage and improved mucosal recovery.

In addition to their direct antioxidant activity, polyphenols may influence cellular redox homeostasis by activating endogenous defense mechanisms. Several flavonoids and phenolic acids have been shown to stimulate the nuclear factor erythroid 2-related factor 2 (Nrf2) pathway, leading to increased expression of antioxidant enzymes, such as superoxide dismutase, catalase, glutathione peroxidase, and heme oxygenase-1 [126,158]. Activation of these cytoprotective pathways may reduce oxidative stress–induced epithelial injury and improve resistance to inflammation-associated barrier dysfunction [119]. Importantly, the biological effects of polyphenols are often dose- and context-dependent, and their antioxidant activity in host tissues does not necessarily translate into identical effects within microbial cells [126].

Maintenance of epithelial barrier integrity is another important consequence of antioxidant and anti-inflammatory modulation. Oxidative stress and chronic inflammation may disrupt TJ proteins, including occludin, claudins, and zonula occludens-1 (ZO-1), resulting in increased intestinal permeability and translocation of microbial products [38,119]. By attenuating inflammatory signaling and oxidative damage, plant-derived polyphenols may help preserve the TJ architecture and support epithelial regeneration [158]. These effects are particularly relevant in GI disorders characterized by impaired barrier function and low-grade chronic inflammation. However, the magnitude of these interactions remains strain-, compound-, dose-, and host-dependent, and requires validation in well-controlled mechanistic and clinical studies [171,172].

#### 4.3.2. Enhancement of Epithelial Barrier Integrity

Flavonoids, such as quercetin and kaempferol, upregulate proteins involved in TJ assembly, including occludin, claudins, and ZO-1, thereby strengthening epithelial barrier integrity and reducing intestinal permeability [38,119]. An improved barrier function may reduce pathogen translocation, limit the passage of microbial toxins and LPS, and support overall mucosal homeostasis [119,173,174,175,176]. These effects are particularly relevant because the disruption of epithelial TJ is associated with chronic inflammation, increased intestinal permeability, and dysregulated host–microbiota interactions in several GI disorders [119,158]. In addition to directly regulating TJ, selected plant-derived flavonoids may enhance epithelial barrier function by reducing oxidative injury and modulating epithelial signaling pathways associated with barrier repair and regeneration [126]. For example, puerarin prevents ethanol-induced TJ dysfunction in a Caco-2 cell model by preserving junctional protein expression and attenuating oxidative stress responses [66,177]. Similarly, apigenin alleviates intestinal ischemia/reperfusion injury by activating Nrf2-dependent cytoprotective pathways and promoting TJ integrity [178]. Other flavonoids, including quercetin and luteolin, have been reported to regulate myosin light-chain kinase (MLCK), NF-κB, and MAPK-associated pathways, which are closely linked to epithelial permeability and inflammation-induced barrier dysfunction [126,158].

The maintenance of epithelial integrity is also closely associated with immune regulation. By preserving the mucosal barrier, plant-derived bioactives may reduce the exposure of underlying immune cells to luminal antigens and microbial products, thereby limiting excessive inflammatory activation [119]. This effect may contribute to improved epithelial recovery following infection, inflammatory injury, or microbiota imbalance [38]. These mechanisms may complement probiotic-mediated reinforcement of the intestinal barrier and contribute to improved gut homeostasis in inflammatory conditions.

#### 4.3.3. Activation of Antimicrobial Immune Pathways

Certain phytochemicals may modulate antimicrobial immune pathways by influencing macrophage and DC activity, enhancing phagocytosis, and promoting AMP production, including defensins and cathelicidins [126,158]. For example, β-defensins play an important role in controlling microbial colonization at mucosal surfaces [158]. Increased AMP production may enhance protection against opportunistic pathogens while contributing to immune homeostasis and balanced inflammatory responses [126]. These innate immune mechanisms constitute an important component of the first line of defense against enteric pathogens and contribute to maintaining mucosal homeostasis [67]. By strengthening antimicrobial surveillance while simultaneously limiting excessive inflammatory responses, plant-derived bioactives may help improve host resistance to infection without directly targeting bacterial viability [126]. Such effects are particularly relevant in the GI tract, where the maintenance of an effective but controlled antimicrobial response is essential for preserving microbiota balance and epithelial integrity.

Several phytochemicals have been reported to influence macrophage polarization, cytokine production and phagocytic activity. For instance, a hydroalcoholic extract derived from oregano triggered a combined anti-mycobacterial and anti-inflammatory response in innate immune cells, suggesting that selected plant extracts may simultaneously enhance pathogen control while reducing inflammatory tissue damage [165,179]. Similar immunomodulatory effects have been described for various polyphenols and terpenoids, which regulate macrophage activation and contribute to a more balanced innate immune response [126,158].

Selected plant-derived compounds may also regulate TLR2- and TLR4-associated signaling cascades or LPS-related signaling, thereby influencing the downstream activation of NF-κB, MAPK, and cytokine-mediated inflammatory responses [126,158,180,181,182,183,184]. For example, dehydrocostus lactone attenuates excessive inflammatory responses associated with TLR signaling [181], whereas 1′-acetoxychavicol acetate inhibited LPS-induced inflammatory activation [184]. By modulating these pathways, phytochemicals may support immune surveillance while preventing excessive inflammation that could otherwise compromise epithelial barrier integrity and tissue repair.

#### 4.3.4. Modulation of Gut Immune-Microbiota Crosstalk

Phytochemicals and their microbiota-derived metabolites may influence immune cell differentiation and gut immune homeostasis, including the expansion of regulatory T cells (Tregs) and attenuation of Th17-associated inflammatory responses [185,186]. These effects are particularly important at the intestinal mucosal interface, where continuous communication between microbial communities, epithelial cells, and immune cells is required to maintain immune tolerance while preserving effective antimicrobial defense [67]. Through interactions with microbiota-produced metabolites, especially SCFAs, plant-derived bioactives may contribute to establishing a balanced immunological environment and improving mucosal resilience [185,186,187,188,189].

A substantial proportion of dietary polyphenols reach the colon largely unmetabolized and undergo extensive biotransformation by gut microorganisms [186,190]. This microbial metabolism generates various smaller bioactive compounds, including phenolic acids, valerolactones, and urolithins, which often exhibit biological activities distinct from those of their parent compounds [185,190]. Several metabolites are associated with anti-inflammatory effects, improved epithelial barrier integrity, and modulation of immune cell signaling pathways involved in intestinal homeostasis [185,186]. Consequently, the biological effects of many phytochemicals depend not only on their original chemical structure but also on the metabolic capacity of the host’s microbiota.

Plant-derived compounds may also indirectly shape immune responses by altering the composition and metabolic activity of the microbial community. Polyphenol-rich diets have been associated with an increased abundance of beneficial microorganisms and enhanced production of anti-inflammatory metabolites, while simultaneously reducing dysbiosis-associated inflammatory signaling [119,186,188,189,191]. These microbiota-mediated effects may contribute to reduced intestinal inflammation, improved epithelial barrier function, and restoration of microbial homeostasis in conditions characterized by chronic, low-grade inflammation [119,185]. These mechanisms are particularly relevant because chronic inflammatory and metabolic disorders are frequently associated with impaired barrier integrity, altered microbial metabolism, and persistent immune activation [186,187,189]. In this context, plant-derived bioactives may complement probiotics by supporting metabolite-mediated communication between microbiota, epithelial cells, and mucosal immune cells [188,189].

From the perspective of probiotic–phytochemical interactions, microbiota-derived metabolites may serve as important mediators linking dietary bioactive compounds with host immune responses. Probiotic microorganisms may enhance the biotransformation of selected phytochemicals, increase the production of SCFAs and other bioactive metabolites, and influence the signaling pathways involved in immune regulation and epithelial homeostasis [67,192]. Consequently, probiotic–phytochemical combinations may influence gut immune–microbiota communication through multiple interconnected mechanisms involving microbial metabolism, epithelial signaling, and immune cell regulation.

However, these effects remain highly dependent on host microbiota composition, phytochemical bioavailability, dietary background, and individual metabotypes [185,186,190]. Considerable inter-individual variability exists in the capacity to generate microbiota-derived metabolites, such as urolithins, which may partly explain the heterogeneous responses observed in human intervention studies [185,190]. Progress in this field depends on integrating microbiome, metabolomic, and immunological analyses to clarify how probiotic–phytochemical combinations modulate host–microbiota interactions across diverse physiological and pathological contexts.

### 4.4. Potential Synergy of Plant Extracts with Probiotics

The antibacterial and immunomodulatory activities of plant extracts provide a mechanistic basis for potential complementary or synergistic interactions with probiotic strains (Table 1). Many phytochemicals weaken pathogen membranes or interfere with QS, making pathogens more susceptible to probiotic-derived bacteriocins, organic acids, and biosurfactants [140,193,194,195,196]. Phytochemicals also enhance probiotic growth and metabolic performance.

These multi-target interactions suggest that plant extracts may act as complementary co-adjuvants, capable of supporting the antimicrobial potential of probiotic strains against antibiotic-resistant pathogens.

## 5. Interactions Between Probiotics and Phytochemicals: From Complementarity to Synergy

Interactions between probiotic microorganisms and plant-derived bioactive compounds range from complementary or additive effects to quantitatively confirmed synergy, representing a promising area for developing microbiota-targeted antimicrobial strategies (Table 2). These biological systems, rather than functioning independently, can enhance each other’s roles in antimicrobial activity, immune regulation, and ecological functions. This interaction results in complex effects that surpass the capabilities of each individual component [286,287,288]. Such interactions may be particularly relevant in the context of antibiotic resistance, as multi-target antimicrobial strategies can reduce selective pressure on single bacterial targets and support antibiotic-sparing approaches when confirmed by appropriate combination assays [136].

### 5.1. Complementary Mechanisms Enhancing Antibacterial Activity

**Figure 2:**
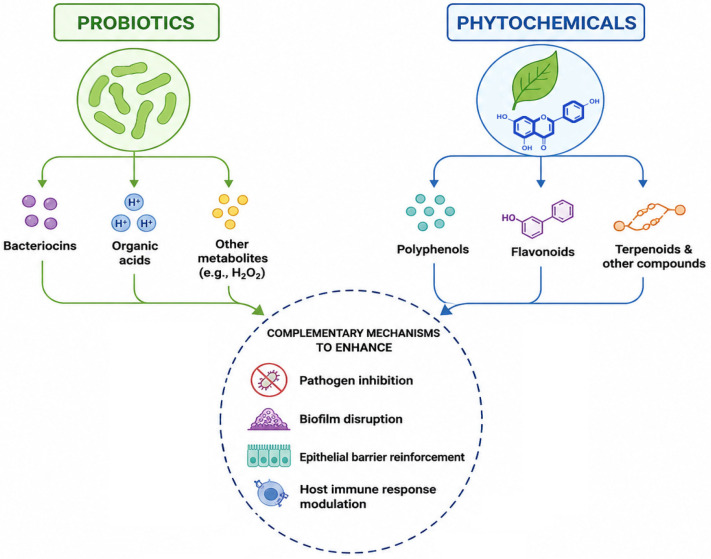
A conceptual interaction network illustrating how probiotic-derived metabolites and phytochemicals may act through complementary mechanisms to support pathogen inhibition, biofilm disruption, epithelial barrier reinforcement, immune modulation, and microbiota-related effects.

Probiotics and phytochemicals frequently target different cellular pathways in pathogens. When applied together, their effects converge to produce additive or synergistic outcomes, depending on the experimental model, antimicrobial endpoint, and quantitative confirmation method [309] (Figure 2). For example, essential oils from *Murraya koenigii* and *Allium sativum* showed enhanced antibacterial activity against *S. aureus* when combined with selected probiotics, such as *L. casei* ATCC 12116, *L. plantarum* NRRL/ATCC 8014, and *Bifidobacterium bifidum* NRRL/ATCC 29521 [310].

#### 5.1.1. Membrane Destabilization and Increased Pathogen Susceptibility

Membrane perturbation and virulence attenuation induced by phytochemicals may increase pathogen susceptibility to probiotic-derived bacteriocins, thereby promoting complementary or potentially synergistic interaction [127,128,311,312,313,314,315,316]. However, such interactions should be interpreted as true synergy only when supported by quantitative assays such as checkerboard, FICI, or time-kill analyses. For example, plantaricin JLA-9 produced by *L. plantarum* JLA-9 disrupts membrane integrity and inhibits oxidative metabolism in germinating *Bacillus cereus* spores, illustrating how probiotic-derived bacteriocins can enhance pathogen inactivation [317].

#### 5.1.2. Amplification of Probiotic Metabolite Production, Adhesion, and Functional Traits

Certain phytochemicals may enhance probiotic growth and metabolic activity, thereby increasing the production of antimicrobial metabolites and strengthening pathogen inhibition [318,319]. For example, tea polyphenols have been shown to promote the growth and metabolic activity of *L. plantarum* CICC 6253 while simultaneously inhibiting *S. aureus* and *E. coli*, illustrating how selected phytochemicals may enhance probiotic fitness and antimicrobial performance [320]. Similarly, plant-derived prebiotics may enhance adhesion-related colonization resistance by modifying probiotic surface properties or stimulating bacterial growth and metabolism [321]. For example, short-chain FOS enhanced the adhesion-related properties of *L. rhamnosus* NCDC 298 and, in combination with this strain, reduced the virulence of ETEC in HT-29 intestinal epithelial cells. FOS alone also reduced ETEC adhesion, indicating that plant-derived prebiotic substrates may support colonization resistance by promoting probiotic functional traits and limiting pathogen attachment [322].

### 5.2. Biofilm Inhibition Through Multi-Target Synergy

Both probiotics and plant extracts can independently inhibit biofilm formation, but their combination has been shown to exert enhanced anti-biofilm effects. For example, the synergistic mixture of *L. acidophilus* and pomegranate peel extract demonstrated stronger anti-biofilm effects against *P. aeruginosa* than did the individual components [323]. Moreover, the combination of *L. plantarum* and compound plant extracts promoted biofilm formation, which was beneficial for probiotic activity while inhibiting pathogenic biofilms, highlighting a dual regulatory effect [324].

Phytochemicals such as carvacrol, cinnamaldehyde, eugenol, tannins, thymol, and selected flavonoids have been shown to disrupt EPS architecture, reduce bacterial adhesion, and inhibit biofilm formation by affecting cell-surface interactions and QS pathways [325,326]. When combined with probiotics or conventional antimicrobials, these mechanisms may contribute to enhanced biofilm control. Plant extracts may also enhance the efficacy of antibiotics against biofilm-forming pathogens. Documented examples include pomegranate and rosemary extracts combined with ciprofloxacin, levofloxacin, gentamicin, or ceftazidime against *P. aeruginosa* biofilms, where complementary anti-biofilm effects were associated with the disruption of biofilm architecture and enhanced antibiotic susceptibility [327]. Additional examples include tea polyphenols combined with *L. plantarum* CICC 6253 and eugenol combined with *L. plantarum* Zs2058, both of which were associated with enhanced antimicrobial and anti-biofilm activities [320,328]. Nevertheless, the efficacy of such combinations is influenced by multiple factors, including phytochemical composition, formulation, and pathogen characteristics [327,329].

#### 5.2.1. Probiotic Biosurfactants + Plant Polyphenols

*Bacillus* biosurfactants (surfactin and fengycin) disrupt biofilm architecture. Polyphenols inhibit QS, thereby reducing EPS production. Upon their combined use, pathogens experience both EPS destabilization and weakened intercellular signaling, which may contribute to biofilm destabilization [330,331,332]. Recent evidence suggests that combinations of probiotic microorganisms with polyphenols, such as proanthocyanidin-rich plant compounds, may enhance anti-biofilm or antifungal activity, although the effects appear to be matrix- and compound-specific and require further validation across different phytochemical sources [169].

#### 5.2.2. Enhanced Coaggregation and Entrapment

Plant polysaccharides can enhance the coaggregation of probiotics and pathogens by strengthening cell–cell and cell–matrix interactions, thereby promoting the formation of mixed microbial aggregates and facilitating pathogen removal via mucosal turnover. This creates a cooperative “entrap and eliminate” mechanism that may reduce pathogen access to epithelial surfaces and limit their colonization. In addition, selected plant-derived polysaccharides and dietary fibers (e.g., inulin, pectins, arabinoxylans, β-glucans, and resistant starch) can stimulate probiotic growth, metabolic activity, and EPS production, further supporting aggregate stability and competitive exclusion [154,333]. Specific probiotic cell wall proteins (e.g., postbiotics from *L. acidophilus* LA-5 and *L. rhamnosus* GG) and exopolysaccharides mediate this interaction and may be enhanced by plant-derived polysaccharides. Polyphenols, such as quercetin and p-coumaric acid, can enhance coaggregation and other probiotic properties, suggesting their potential for synbiotic applications [334,335].

### 5.3. Quorum-Sensing Disruption Reinforced by Mixed Biological Signals

Both probiotics and phytochemicals possess quorum-quenching properties; however, they operate via different mechanisms. While probiotics mainly contribute through enzymatic degradation of signaling molecules and microbial homeostasis, phytochemicals may interfere with QS by acting as signal mimics and inhibitors [336,337]. For example, quercetin combined with *L. acidophilus* LA5 enhanced probiotic autoaggregation and coaggregation with *E. coli*, reduced pathogen adhesion to differentiated Caco-2 cells, and improved epithelial barrier function by increasing TEER values and claudin-1 expression, while reducing pro-inflammatory COX-2 expression [334]. Similarly, the combination of *L. fermentum* ASBT-2 and oxyresveratrol suppressed the motility and virulence-associated traits of *Salmonella enterica* more effectively than either component alone, suggesting complementary anti-virulence activity [338].

These observations support the concept that probiotic–phytochemical combinations may reinforce pathogen colonization control and virulence attenuation, although direct QS interactions require further investigation [111,339,340,341,342,343].

### 5.4. Co-Modulation of the Host Immune System

The interaction between probiotics and phytochemicals extends beyond direct antibacterial action and may influence the host immune responses. In selected models, combined probiotic–phytochemical formulations, including those evaluated in atopic dermatitis, have shown stronger anti-inflammatory or microbiota-modulating effects than individual components by promoting beneficial bacteria (e.g., *Bifidobacterium* and *Akkermansia*), suppressing pathogens, and reducing inflammatory cytokines [292,344]. Together, these mechanisms may support a more resilient mucosal environment that limits pathogen invasion [345,346].

### 5.5. Biotransformation of Plant Compounds by Probiotic Strains

Probiotic microorganisms can biotransform complex phytochemicals into more bioavailable and biologically active metabolites. Probiotic bacteria, including strains such as *L. brevis* DSM 6235, *L. plantarum* DSM 20205, *L. paracasei* DSM 20312, and *L. rhamnosus* NCTC 10302, possess glycosyl hydrolases, such as β-glucosidases, that cleave sugar moieties from phytochemical glycosides, thereby releasing more bioavailable and biologically active aglycones. For example, citrus flavonoids, such as hesperidin and naringin, can be converted into hesperetin and naringenin, respectively, which often display enhanced antioxidant and anti-inflammatory activities compared with their parent compounds [347]. Microbial fermentation of *Syzygium cumini* kernels using curd and yogurt starter cultures also generates metabolites with antibacterial, antioxidant, and anti-inflammatory properties [348]. Such biotransformation processes may enhance the bioavailability, bioactivity, and pharmacological potential of plant-derived compounds, thereby increasing the functional value of phytochemicals in probiotic–phytochemical formulations.

Metabolic crosstalk with phytochemicals can potentiate these effects on the gut microbiota. Certain polyphenols such as catechins, anthocyanins, and proanthocyanidins upregulate probiotic metabolic pathways, boosting SCFA and bacteriocin production [349,350,351,352,353]. Selected probiotic strains of LAB, including *L. plantarum*, *L. fermentum*, and other *Lactobacillus s.l.* representatives, can biotransform plant polyphenols into bioactive derivatives, including aglycones and phenolic acids. For example, Wang et al. [354] investigated the probiotic transformation of fruit polyphenols, whereas LAB were shown to participate in the bidirectional metabolism of finger millet polyphenols [355]. In addition, bioconversion by probiotic bacteria such as *L. reuteri* DSM 20016, *Enterococcus faecalis* M74 and *Bifidobacterium breve* ATCC 15701 increased the antiradical activity of lotus seed epicarp polyphenols [356]. However, the conversion of ellagitannins into urolithins is mainly associated with specialized gut microbiota members (e.g., *Gordonibacter urolithinfaciens*, *Gordonibacter pamelaeae*, and *Ellagibacter isourolithinifaciens*) rather than classical probiotic strains; therefore, urolithin production should be discussed as a microbiota-dependent transformation rather than a general *Lactobacillus/Bifidobacterium* function [357].

### 5.6. Reduced Antibiotic Requirement Through Potent Synergy

By simultaneously disrupting pathogen membranes, inhibiting quorum signaling, suppressing virulence, and supporting host defenses, probiotic–phytochemical combinations may reduce the minimum effective concentration of selected antibiotics [104,358]. This supports the development of antibiotic-sparing therapeutics, which is a critical approach for mitigating AMR.

### 5.7. Conceptual Model of Probiotic–Phytochemical Synergy

The emerging model suggests that synergy arises through the following:

1. Direct microbial antagonism (multi-target inhibition of pathogens);
2. Metabolic cooperation (biotransformation + enhanced probiotic metabolite production);
3. Immune co-modulation;
4. Ecological stabilization of the microbiota;
5. Barrier reinforcement;
6. Biofilm eradication.

This multifaceted cross-domain interaction underscores the translational potential of combined probiotic–phytochemical interventions (Table 3).

## 6. Methodological Approaches to Evaluate Probiotic–Phytochemical Interactions

**Figure 3:**
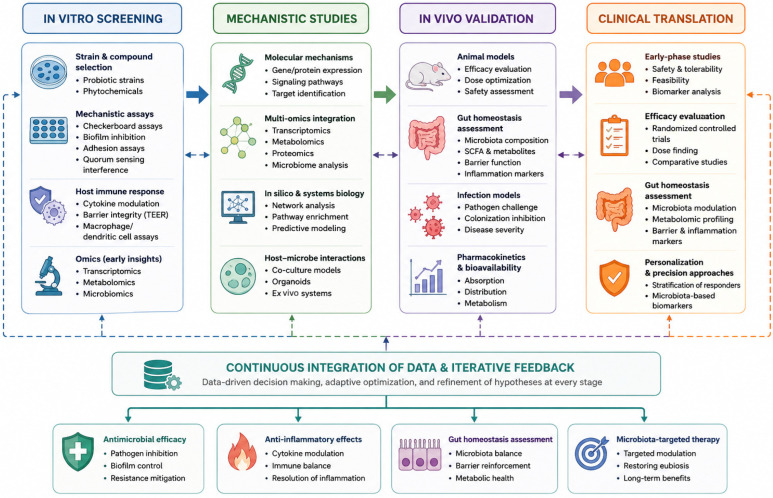
Translational framework for the development of probiotic–phytochemical combinations from in vitro screening and mechanistic validation to formulation, safety evaluation, clinical application, and real-world implementation.

A major methodological limitation in this field is the inconsistent application of the term “synergy.” In this review, quantitative synergy refers only to interactions confirmed by formal assays, such as checkerboard analysis with FICI calculation or time-kill experiments showing enhanced pathogen reduction compared with the most active single treatment [363]. In the absence of formal synergy testing, the combined effects are described as additive, complementary, or mechanistically plausible interactions. Because probiotic–phytochemical interactions may involve direct antimicrobial activity, biofilm modulation, metabolic crosstalk, epithelial barrier protection, and immune regulation, multiple endpoints are often necessary (Figure 3) [364,365]. However, methodological interpretation should remain conservative: growth inhibition, improved probiotic viability, altered cytokine production, or biofilm reduction alone should not be considered proof of synergy unless appropriate single-agent controls and quantitative interaction metrics are included. In biofilm-based models, complementary structural or molecular readouts may be useful because biofilm tolerance reflects multiple interacting processes, including diffusion, metabolism, gene expression, and physiological heterogeneity [130,366].

The principal methodological approaches and recommended endpoints are presented in Table 4.

## 7. Potential Clinical, Biotechnological, and Nutraceutical Applications

Emerging evidence suggests that the interactions between probiotics and plant-derived phytochemicals may be relevant to clinical medicine, biotechnology, and the rapidly growing nutraceutical sector [324,405,406]. These combined bio-interventions may provide multi-target antimicrobial action, immune modulation, and microbiota support, which aligns with current efforts to develop sustainable and biologically based strategies for gut health and antimicrobial protection [294,407]. However, most interactions between probiotics and plant-derived bioactives have been investigated in vitro or in animal models. Only a limited number of human studies have evaluated combined interventions involving probiotic strains and plant-derived bioactives (Table 5). These studies provide preliminary translational evidence; however, their interpretation remains limited by short intervention periods, heterogeneous formulations, small or disease-specific cohorts, and the frequent absence of formal synergy metrics.

Table 5 includes human studies evaluating combined interventions involving probiotic strains and plant-derived bioactive compounds; however, not all studies were specifically designed to quantify the synergy between both components.

### 7.1. Potential Clinical Applications

#### 7.1.1. Potential Support in Gastrointestinal Infection Management

Probiotic–phytochemical combinations represent promising alternatives or adjuncts to antibiotic therapy for GI infections caused by *C. difficile*, *Salmonella*, *E. coli*, *S. aureus*, and other pathogens [108,414]. Their benefits arise from the restoration of healthy microbiota balance, suppression of pathogen growth via multi-target inhibition, reinforcement of epithelial barrier integrity, and reduction of inflammation and toxin-mediated damage. Such formulations may potentially reduce relapse risk and lessen the need for broad-spectrum antibiotics, thereby mitigating dysbiosis and slowing the emergence of resistance [407,415].

#### 7.1.2. Management of Inflammatory Bowel Disease (IBD) and Irritable Bowel Syndrome (IBS)

Phytochemicals with anti-inflammatory and antioxidant activities (e.g., curcumin, resveratrol, and quercetin) combined with probiotic strains (*Lactobacillus s.l.*, *Bifidobacterium*, and *Bacillus*) can modulate cytokine profiles, improve mucosal healing, and restore gut barrier function. Combined interventions may reduce oxidative stress and mucosal damage, increase SCFA production, and promote the normalization of dysregulated immune response. Such combinations have shown encouraging results in preclinical models, paving the way for future clinical trials [416,417,418,419].

#### 7.1.3. Potential Support in Oral and Dermal Infection Prevention

Topical and oral formulations combining probiotics and plant extracts have shown promising activity in preliminary studies against opportunistic pathogens in the oral cavity (*Candida* spp. and *Streptococcus mutans*) and skin (*S. aureus* and *P. aeruginosa*). Benefits include inhibition of biofilm formation, suppression of virulence factors, reduced pathogen adhesion, and enhancement of local immune defenses. The generally favorable safety profiles support further evaluation of preventive care formulations [420,421,422,423].

#### 7.1.4. Antibiotic-Sparing Therapeutics

A potential clinical advantage of probiotic–phytochemical combinations is their capacity to reduce the required antibiotic dosage while maintaining beneficial antimicrobial effects. This approach decreases selective pressure for resistant strains, preserves commensal microbiota, and lowers the risk of adverse drug effects. These combinations align with global antimicrobial stewardship efforts. Moreover, combining plant extracts with antibiotics or probiotics has been reported to reduce the minimum inhibitory concentration (MIC) of antibiotics in selected experimental models, lowering the required dosage and minimizing side effects [424,425].

### 7.2. Biotechnological Applications

#### 7.2.1. Development of Synbiotic Formulations

Documented or proposed probiotic–phytochemical interactions may support the development of microbiota-targeted synbiotic formulations, in which plant-derived bioactives function not only as prebiotic substrates but also as antimicrobial and immunomodulatory co-effectors in the gut. This new class integrates probiotics with proven antagonistic activity, phytochemicals that enhance metabolite production, and formulations optimized using metabolomic and transcriptomic profiling [4,426,427,428,429].

#### 7.2.2. Fermentation Technology and Metabolite Bioengineering

Probiotic fermentation of phytochemicals generates more bioactive metabolites such as hydroxycinnamic acid derivatives and activated flavonoids. Industrial applications include the biotechnological production of health-promoting compounds, natural antimicrobial preservatives, and valorization of plant waste streams [430,431,432,433].

#### 7.2.3. Biocontrol in Agriculture and Food Safety

Probiotic–phytochemical combinations can be integrated into biocontrol strategies to reduce bacterial contamination in agriculture [434,435], aquaculture [436,437], and food processing [438]. The potential advantages of this approach include an environmentally friendly profile, multi-target pathogen suppression, and compatibility with organic farming practices. This reduces reliance on chemical antibiotics in livestock and crop protection [439,440].

### 7.3. Nutraceutical and Functional Food Applications

#### 7.3.1. Functional Foods with Enhanced Antimicrobial and Immunomodulatory Activity

There is an increasing demand for foods enriched with probiotics and phytochemicals to support gut health, immunity, and microbiota balance. The food matrices used include yogurt and fermented dairy, kombucha and fermented beverages, plant-based fermented foods, and encapsulated-supplement formats. Combined probiotic–phytochemical formulations may offer additional health benefits compared to traditional single-component products, although further clinical validation is required [294,441,442,443].

#### 7.3.2. Encapsulation and Controlled-Release Technologies

Advanced encapsulation techniques (e.g., alginate beads, microcapsules, and biopolymer composites) can co-deliver probiotics and phytochemicals to the gut. Their benefits include targeted release in the intestine, enhanced viability of probiotics, protection of sensitive phytochemicals from degradation, and controlled modulation of the microbiota composition [444,445,446,447].

#### 7.3.3. Personalized Nutrition and Microbiome-Targeted Products

The integration of metagenomics and personalized nutrition trends has led to the development of microbiota-tailored nutraceuticals. Probiotic–phytochemical combinations fit this paradigm by offering formulations that are tuned to individual microbial profiles, optimized for immune or metabolic outcomes, and adaptable to specific populations (elderly, athletes, IBS patients, etc.) [448,449,450,451].

## 8. Regulatory and Safety Considerations

Although probiotics and plant-derived bioactives are generally considered safe when used individually, their combined use in concentrated formulations necessitates thorough safety and regulatory evaluations. First, plant-derived bioactives may exert dose-dependent effects; compounds that are beneficial at nutritional levels may become cytotoxic, pro-oxidant, hepatotoxic, or irritant at high concentrations or after prolonged exposure. Therefore, concentrated extracts should be chemically standardized and evaluated for toxicity, acceptable daily intake, contaminants, solvent residues, and potential interactions with drugs or host metabolism [452,453,454].

Second, phytochemicals may negatively affect the viability of probiotics. Some phenolics, essential oils, alkaloids, and tannins can inhibit bacterial growth, disrupt membranes, or reduce metabolic activity, which may compromise the survival of probiotic strains during manufacturing, storage, GI transit, and co-delivery. Therefore, compatibility testing between each probiotic strain and plant-derived bioactives should be performed before formulation, including viability assays, stress tolerance tests, and stability studies under relevant pH, oxygen, temperature, and storage conditions [455,456].

Third, strain safety is essential. Probiotic candidates should be identified at the strain level, preferably using whole-genome sequencing, and screened for virulence factors, transferable antibiotic resistance genes, toxin production, hemolytic activity, and undesirable metabolic traits. This is particularly important for non-LAB probiotics and spore-forming bacteria, for which safety cannot be inferred from the species name alone [453,455].

Fourth, microbiome-related risks should be considered in future studies. Probiotic–phytochemical combinations may have off-target effects on commensal microorganisms, alter microbial diversity, or shift metabolic outputs in a host-dependent manner [171,457]. These effects may be beneficial in some contexts but undesirable in others, especially in vulnerable populations, including immunocompromised individuals, infants, older adults, and patients with severe dysbiosis [171,458]. Interindividual variability in phytochemical metabolism and gut microbial metabotypes may further influence biological responses to these interventions [185,186]. Therefore, it is important to monitor the microbiome and include metabolomic endpoints to determine the intended and unintended ecological effects in future studies [459].

Finally, formulation-related risks must be considered. Co-formulation may modify the stability, bioavailability, release kinetics, and biological activity of probiotics and phytochemicals [460,461]. Encapsulation, microencapsulation, and controlled-release systems can improve probiotic survival and targeted delivery. Nonetheless, these methods may modify dose exposure, elevate production expenses, and necessitate further validation for safety and stability [461,462,463]. Regulatory classification remains challenging because probiotic–phytochemical products may fall between functional foods, dietary supplements, nutraceuticals, and therapeutic products [463,464,465]. Consequently, transparent reporting of strain identity, phytochemical composition, dosage, formulation matrix, viability, stability, and intended use is essential for the reproducibility, consumer safety, and regulatory evaluation of probiotic and plant-derived bioactive formulations [464,466].

Briefly, phytoprobiotic formulations require the following [452,453,454,455,456]:

- toxicity testing of concentrated phytochemicals;
- stability and viability assessment of the final formulation;
- clear documentation of genomically verified probiotic strain identity;
- compliance with the European Food Safety Authority (EFSA) and Food and Drug Administration (FDA) guidelines for probiotics and botanical supplements.

The regulatory landscape is evolving to accommodate potential synergistic bioactive combinations in functional foods and dietary supplements.

## 9. Limitations, Challenges, and Future Perspectives

Despite the growing interest in the synergistic interactions between probiotics and plant-derived phytochemicals, several conceptual, methodological, and translational challenges remain. Addressing these limitations is essential for advancing the field toward clinically validated, standardized, and commercially viable therapeutic strategies [467].

### 9.1. Limitations and Current Challenges

#### 9.1.1. Variability in the Composition of Plant Extracts

Plant extracts are inherently heterogeneous, and their chemical composition is influenced by factors such as cultivation conditions, plant age, extraction methods, and storage. This variability creates challenges in terms of reproducibility across studies, standardization of doses, regulatory approval, and consistency of clinical outcomes. Phytochemical fingerprinting (untargeted LC-MS/MS, NMR) is often required but not always performed in probiotic–phytochemical studies [468,469].

#### 9.1.2. Strain-Specific Response of Probiotics

- Probiotic responses to plant-derived bioactives are strongly strain-specific and cannot be reliably inferred from species-level taxonomy alone [466,470]. Strains belonging to the same species may differ in membrane composition, stress response systems, carbohydrate utilization pathways, bile and acid tolerance, adhesion capacity, bacteriocin production, and enzymatic machinery involved in phytochemical biotransformation [192,466,471]. These differences determine whether a given plant extract stimulates, has no measurable effect on, or inhibits probiotic growth [95,144]. For example, polyphenol-rich extracts may support selected *Lactobacillus* or *Bifidobacterium* strains by acting as fermentable substrates or metabolic modulators, whereas the same extracts may suppress other strains because of the presence of membrane-active phenolics, tannins, or essential oil constituents [95,144,192]. Therefore, probiotic–phytochemical combinations should be designed and tested at the strain level, with viability, metabolic activity, adhesion properties, and phytochemical biotransformation capacity assessed under formulation-relevant and GI conditions [172,466,472]. This strain-level variability is one of the main reasons why results obtained for one probiotic strain cannot be generalized to other strains, even within the same species [171,466].

#### 9.1.3. Lack of Standardized Synergy Definitions and Protocols

Although checkerboard and time-kill assays are common, there is no universal standard for defining synergy between biological agents. The differences across laboratories include the following [468,473,474]:

- Inoculum sizes;
- Extraction solvents;
- Culture media;
- Endpoints used to define inhibition;
- Interpretation thresholds.

This lack of harmonization hinders cross-study comparisons and meta-analyses.

#### 9.1.4. Limited in Vivo and Clinical Evidence

Most synergy research remains at the in vitro level. Challenges include differences in the in vivo bioavailability of phytochemicals, survival and colonization of probiotics in the gut, rapid metabolism of plant compounds, and host-specific microbiota response. Only a few studies have validated the synergistic effects in animal models, and even fewer have extended this validation to humans [107,468,473,475].

#### 9.1.5. Challenges in Co-Formulation and Stability

The combination of probiotics and plant extracts introduces significant formulation issues, including the sensitivity of probiotics to pH, oxygen, heat, and solvents; instability of polyphenols during processing; antagonism between certain strains and compounds; and difficulty in synchronizing their release profiles. Advanced encapsulation technologies can mitigate these problems; however, they also increase production costs [476,477].

#### 9.1.6. Regulatory Ambiguity

Current regulatory frameworks for probiotics and botanical supplements are not fully adapted to combined biological therapies. Regulators often lack clear categories for synbiotic formulations with therapeutic claims, probiotic–phytochemical medicinal products, and biotransformed phytochemicals produced during fermentation. This regulatory gap complicates commercialization and clinical trial designs [469,475].

The major limitations and corresponding technological priorities for advancing probiotic–phytochemical strategies are summarized in Table 6.

Together, these challenges highlight the need for integrated, standardized, and multilevel approaches to fully realize the therapeutic potential of probiotic–phytochemical synergy.

### 9.2. Future Perspectives

#### 9.2.1. Precision Synbiotics and Personalized Microbiome Interventions

One of the most promising future directions is the development of precision synbiotics, defined as rationally selected combinations of probiotic strains and plant-derived bioactives tailored to the microbiome composition, metabolic capacity, and clinical or nutritional context [305,494]. This approach is particularly relevant because probiotic effects are strain-specific, whereas phytochemical metabolism strongly depends on the functional capacity of the resident gut microbiota [190,466]. For example, interindividual differences in microbial metabotypes may determine whether dietary ellagitannins are efficiently converted into urolithins, thereby influencing the biological response to pomegranate and berry-derived polyphenols [185,190]. Therefore, the successful implementation of probiotic–phytochemical interventions will depend not only on the botanical source or probiotic species but also on strain-level functionality and phytochemical biotransformation capacity [192,466].

Future advances in this area are likely to incorporate metagenomic diagnostics, metabolomic profiling, machine-learning prediction of promising probiotic–phytochemical pairs, and microbiome-based participant stratification [491,495,496]. However, precision synbiotic design will require standardized reporting of probiotic strain identity, phytochemical composition, dose, formulation, viability, and stability to ensure reproducibility and facilitate validation across studies [172,466]. Currently, personalized probiotic–phytochemical approaches remain an emerging area of research rather than an established clinical tool, and their translation into practice will require robust human validation studies, reproducible biomarkers of response, and clinically meaningful outcomes [171,172].

#### 9.2.2. Translational Challenges

Several additional factors complicate the translational development of probiotic–phytochemical combinations. Pharmacokinetic and pharmacodynamic challenges arise because many phytochemicals exhibit limited oral bioavailability, rapid metabolism, chemical instability, and significant interindividual variability in absorption and microbial biotransformation [497]. In parallel, probiotic viability, colonization efficiency, and metabolite production are strongly influenced by the formulation, gastrointestinal conditions, and host microbiota composition [498]. Clinical trial design is further complicated by differences in probiotic strains, phytochemical preparations, dosages, treatment duration, dietary backgrounds, and outcome measures [499]. Variability in the host microbiota and responder phenotypes may also contribute to heterogeneous clinical responses. Consequently, standardized formulations, validated biomarkers, and adequately powered randomized controlled trials are required to facilitate the successful translation of these findings into clinical practice [499,500].

#### 9.2.3. Multi-Omics Integration

A robust understanding of probiotic–phytochemical interactions requires the integration of multiple omics technologies, as no single analytical approach can fully capture the complexity of microbial metabolism, host responses, and microbiota-mediated effects [459,493]. Metabolomics can identify changes in SCFA production, phytochemical biotransformation products, and other microbiota-derived metabolites, whereas genomics, transcriptomics, and proteomics provide complementary information on strain functionality, gene expression, metabolic pathways, and host–microbe interactions [171,172,459,466,493].

Integrated multi-omics approaches may help distinguish direct antimicrobial effects from indirect microbiota- or host-mediated responses and facilitate the identification of biomarkers associated with treatment responsiveness and microbial resilience [67,185,186]. However, the meaningful integration of multi-omics datasets remains challenging because of differences in analytical platforms, bioinformatic pipelines, and reporting standards [459]. The generation of consistent and biologically meaningful mechanistic insights will require standardized experimental frameworks combined with comprehensive microbiome and multi-omics analyses [171,172].

#### 9.2.4. High-Throughput Screening Platforms

The identification of effective probiotic–phytochemical combinations is challenging because of the large number of possible interactions among probiotic strains, phytochemicals, doses, formulations, and target microorganisms. Therefore, automated and miniaturized screening platforms may become increasingly important for prioritizing promising combinations for mechanistic validation [501,502]. Microfluidic and other miniaturized assay systems enable efficient testing of microbial growth, viability, antimicrobial activity, and dose–response relationships while reducing reagent consumption and experimental time [503,504,505]. Such platforms can be adapted to evaluate probiotic strain collections, plant-derived compounds, and complex microbial community models of the gut. However, preliminary screening results do not constitute evidence of synergy unless confirmed by quantitative methods, such as checkerboard, FICI, or time-kill assays.

Artificial intelligence and machine-learning approaches may further support this process by prioritizing combinations with a higher probability of functional compatibility or antimicrobial synergy [506,507,508]. Nevertheless, these tools are primarily hypothesis-generating rather than confirmatory, as predicted interactions still require experimental validation in microbiological, biofilm-, microbiome-based, and ultimately, clinical models.

#### 9.2.5. Next-Generation Delivery Systems

The development of advanced delivery technologies will be crucial, including microencapsulation (alginate, chitosan, lipid-based systems), layered microgels with dual-release kinetics, targeting ligands for colon-specific release, and encapsulated probiotics co-formulated with stabilized phytochemicals. These systems can improve viability, absorption, and target activity [509,510,511].

The successful implementation of probiotic–phytochemical formulations will depend on delivery systems capable of improving probiotic survival and targeted delivery while maintaining compatibility between probiotics and phytochemicals throughout storage and gastrointestinal transit [172,325]. Factors such as release kinetics, matrix composition, moisture sensitivity, oxygen exposure, and phytochemical–microbe interactions may substantially influence the performance of the formulation [325,472]. Advanced encapsulation and controlled-release technologies may protect probiotic cells from acid, bile, oxygen, and processing stress, while also stabilizing sensitive phytochemicals against oxidation or degradation [38,325]. Ensuring consistent biological activity and reproducible results will require viability testing, stability evaluation, dose-exposure analysis, characterization of release profiles, and safety validation [171,172].

#### 9.2.6. Eco-Friendly Biocontrol and Agricultural Applications

As concerns regarding antibiotic use in agriculture grow, probiotic–phytochemical combinations may represent eco-friendly candidates for future prevention-oriented strategies in crop pathogen management, livestock health support, and aquaculture microbiome modulation, although further validation under field conditions is required to confirm their efficacy. These applications may be particularly relevant for sustainable agriculture and food safety [512,513,514].

The translation of probiotic–phytochemical strategies to agriculture, aquaculture, and livestock production will require validation under realistic production conditions [11,515]. The successful implementation of probiotic–phytochemical strategies in agricultural settings will require long-term field studies evaluating not only pathogen suppression but also formulation persistence, probiotic survival, phytochemical stability, and consistency of performance across different environmental settings [172,325]. In aquaculture and animal production models, probiotic and plant-derived interventions may influence immune gene expression, intestinal microbiota composition, and disease resistance; however, these effects remain strongly dependent on host species, diet, formulation, and environmental conditions [10,515]. Assessment of potential unintended impacts on native microbial populations, ecosystem functions, and non-target organisms, including beneficial insects, soil microbes, and aquatic microorganisms, is also necessary [171,186]. Such data are essential for establishing the ecological safety, sustainability, and practical applicability of probiotic–phytochemical formulations as environmentally friendly alternatives or complements to conventional antimicrobial interventions [10,172].

#### 9.2.7. Clinical Translation and Therapeutic Validation

Although numerous in vitro and preclinical studies suggest beneficial interactions between probiotics and phytochemicals, their clinical translation remains limited [171,172]. Clinical translation will require well-designed RCTs capable of distinguishing the individual and combined contributions of probiotic and phytochemical components. Factorial trial designs incorporating probiotic-only, phytochemical-only, combination, and placebo groups would provide a more rigorous assessment of the additive, complementary, or potentially synergistic effects under clinical conditions [171]. There is also a need for pharmacokinetic and pharmacodynamic studies of phytochemical–probiotic interactions, exploration of synergy in chronic inflammatory conditions, and evaluation of post-antibiotic microbiome restoration [516,517].

Clinical translation will require the integration of conventional clinical outcomes with microbiome-related, metabolomic, and inflammatory endpoints to improve mechanistic understanding and facilitate biomarker identification [185,186,459]. Greater consideration of dose optimization, treatment duration, formulation characteristics, and interindividual variability will also be necessary, as these factors may substantially influence treatment outcomes [185,190].

Long-term safety monitoring remains an essential component of clinical evaluation. A comprehensive assessment of both efficacy and safety is necessary to identify potential adverse effects, including microbiome disturbances, unintended metabolic consequences, and risks associated with prolonged exposure to concentrated phytochemicals or complex formulations, particularly in vulnerable populations [171,172]. Ultimately, successful clinical translation will require robust human validation studies, harmonized trial designs, and comprehensive integration of clinical, microbiological, metabolomic, and safety data [171,172,466].

#### 9.2.8. Phage-Based Strategies as Complementary Approaches to Probiotic–Phytochemical Interventions

Phage-based strategies may represent a complementary future direction rather than being a central component of probiotic–phytochemical formulations. Bacteriophages specifically target bacterial hosts and are increasingly considered adjunctive tools against MDR and biofilm-associated infections [518,519,520]. However, phage–phytochemical combinations cannot be assumed to be intrinsically synergistic because plant-derived compounds may either reduce or enhance phage stability, infectivity, and antibacterial efficacy depending on the extract composition, phage type, formulation matrix, and environmental conditions [521,522,523,524]. Therefore, the successful implementation of phage-based multi-component antimicrobial strategies will depend on compatibility testing, formulation optimization, microbiome safety assessment, and clinical validation.

## 10. Conclusions

The combination of probiotics and plant-derived bioactives may represent a promising multi-target strategy for addressing the escalating challenges of AMR, dysbiosis, and chronic inflammatory conditions. While both probiotics and phytochemicals individually possess well-established antimicrobial, immunomodulatory, and barrier-protective properties, their combination may offer enhanced efficacy through complementary mechanisms of action. These include disruption of pathogen membranes, interference with QS, inhibition of virulence factors, reinforcement of epithelial integrity, modulation of host immunity, and metabolic biotransformation of phytochemicals into more active derivatives.

Despite their substantial potential, significant challenges remain, including variability in plant extract composition, strain-specific probiotic responses, lack of standardized synergy protocols, and limited translational evidence, all of which hinder their clinical integration. In addition, the clinical translation of probiotic–phytochemical interventions is complicated by the limited bioavailability and extensive metabolism of many phytochemicals, variability in probiotic survival and colonization, interindividual differences in host microbiota composition, and the complexity of designing and conducting standardized clinical trials. Addressing these pharmacokinetic, pharmacodynamic, and methodological challenges will be essential for developing evidence-based microbiota-targeted interventions. Nevertheless, advances in multi-omics, computational modeling, precision microbiome profiling, and advanced delivery systems are facilitating the rational design and evaluation of probiotic–phytochemical combinations.

As research continues to uncover the mechanistic basis and translational relevance of these synergistic interactions, probiotic–phytochemical combinations could become useful resources in clinical and applied microbiome studies, biotechnology, and personalized nutrition. Their capacity to combine antimicrobial-supportive activity with microbiota and host-health support highlights their potential as microbiota-targeted antimicrobial and immunomodulatory interventions in the post-antibiotic era.

## Figures and Tables

**Table 1:** Examples of plant-derived compounds with antimicrobial properties and their mechanisms relevant to interaction with probiotics.

Plant Source/Key Phytochemical(s)	Target Pathogens	MIC/Effective Concentration/Probiotic Compatibility/Safety Note (If Reported)	Mechanism(s) of Action	Type of Interaction	References
*Allium sativum* (garlic)/allicin	Broad-spectrum bacteria and fungi	Fresh garlic extract (2.5–20 mM) exhibited concentration-dependent antibacterial activity, with MIC values of approximately 6.25% for *E. coli* and 12.5% for *P. aeruginosa*, and activity against MDR/MRSA strains. Oxidative/disulfide stress-related toxicity. Growth inhibition was observed in yeast and *Arabidopsis* models at low micromolar concentrations (≈2.5–12.5 µM), with markedly increased sensitivity in glutathione-deficient mutants. Dose-dependent cytotoxicity at higher concentrations (>50–100 μM), associated with thiol oxidation, glutathione depletion, and cytoskeletal disruption	Inhibits thiol-containing enzymes; disrupts metabolism	May sensitize pathogens to probiotic-derived antimicrobials, although direct garlic–bacteriocin synergy remains to be confirmed	[197,198,199,200,201]
*Camellia sinensis* (green tea)/epigallocatechin gallate (EGCG)	*Staphylococcus aureus*, *Escherichia coli*, *Candida albicans*	EGCG showed bactericidal activity against MDR bacteria, with reported MBC values of 1250 µg/mL for MDR *E. coli* and 625 µg/mL for MDR *S. aureus;* green tea extract showed MIC values of approximately 125 µg/mL. Cytotoxic and pro-oxidant effects in CHO-K1 cells at 0.5–1 µM EGCG (decreased viability, increased ROS, altered mitochondrial membrane potential). Dose-dependent hepatotoxicity, manifested by increased serum transaminases (ALT/AST), observed at ≥800 mg EGCG/day; rare idiosyncratic severe liver injury reported at 375 mg EGCG/day.	Membrane permeabilization; efflux pump inhibition; ROS generation	May sensitize pathogens to probiotic-derived antimicrobials through membrane disruption and redox imbalance; however, direct synergy with bacteriocins remains to be confirmed	[113,114,202,203,204,205,206,207]
*Cannabis sativa*/cannabidiol (CBD)	Gram-positive bacteria: MRSA, MSSA, *Streptococcus pneumoniae*, *E. faecalis*, *C. difficile*, *C. acnes*; selected Gram-negative bacteria: *Neisseria gonorrhoeae*, *Neisseria meningitidis*, *Moraxella catarrhalis*, *Legionella pneumophila*	MIC mostly 1–4 µg/mL against Gram-positive bacteria; MRSA MIC90 4 µg/mL in Australian isolates and MIC50/MIC90 1/1 µg/mL in USA isolates; *N. gonorrhoeae* MIC50/MIC90 2/2 µg/mL in broth and 2/4 µg/mL in agar assay. Active against MSSA/MRSA biofilms; MBEC 1–2 µg/mL for MSSA and 2–4 µg/mL for MRSA; confocal microscopy showed biofilm penetration and killing. Topical activity was formulation-dependent; 5–20% CBD topical formulations reduced MRSA load in ex vivo pig skin and 5% CBD showed activity in a mouse topical skin infection model. Low propensity to induce resistance in MRSA after serial passage; no hemolysis up to 256 µg/mL; modest HEK-293 cytotoxicity, CC50 around 200 µg/mL. Animal studies have consistently identified the liver as a sensitive target organ. A 90-day OECD TG 408 study in rats established a NOAEL of 50 mg/kg bw/day, whereas benchmark dose modelling yielded a BMDL10 of 11 mg/kg bw/day. Additional concerns include reproductive and neurodevelopmental toxicity, and endocrine effects. The EFSA derived a provisional safe intake of 0.0275 mg/kg bw/day (~2 mg/day for a 70-kg adult)	Rapid bactericidal activity; primary mechanism: bacterial membrane disruption; membrane depolarization and increased permeability; inhibition of macromolecular synthesis secondary to rapid killing	Antibacterial synergy through outer membrane permeabilization by polymyxin B enables CBD activity against Gram-negative pathogens	[208,209]
*Cannabis sativa*/cannabidiol (CBD), cannabigerol (CBG)	*Streptococcus mutans*, oral multispecies biofilms, *Vibrio harveyi*, *S. aureus*, *E. coli*, *P. aeruginosa*	CBD MIC 20 µM and CBG MIC 10 µM against *S. mutans*; CBD/CBG MIC/lethal concentrations against *P. aeruginosa* and *E. coli* reported in the range 400–3180 µM. In vitro fibroblast viability remained >95% for CBD and >88% for CBG in an oral-cell model. No mortality or histopathological changes were observed in rats after oral CBG administration (35–140 mg/kg bw/day for 14 days). Minor increases in ALP and chloride levels at 140 mg/kg bw/day were not considered toxicologically relevant. Data on genotoxicity and reproductive toxicity are lacking.	Membrane disruption; anti-biofilm activity; CBG-mediated QS/biofilm inhibition	Potential anti-biofilm/antivirulence phytochemicals; CBG prevents QS and biofilm formation in *V. harveyi*. Direct probiotic compatibility remains to be evaluated	[210,211,212]
*Cinnamomum verum* (cinnamon)/cinnamaldehyde	*E. coli*, *Enterococcus faecalis*, *Porphyromonas gingivalis*, *S. aureus*, *Candida* spp.	Cinnamon bark essential oil and cinnamaldehyde exhibited MIC values of 6.25 µg/mL and 2.5 µM, respectively, against *Porphyromonas gingivalis*, and both inhibited biofilm formation at sub-MIC levels. Essential oil inhibited *E. coli* isolates in a well-diffusion assay, producing inhibition zones of 4.5–5.2 cm. Cinnamaldehyde derivatives exhibited modest antibacterial activity against *E. coli* and *S. aureus* at mM concentrations. Dose-dependent cytotoxicity has been reported for cinnamon essential oil in rat bone marrow mesenchymal stem cells, with an LC50 of 0.004% and marked reductions in cell viability at 0.0312–0.5% concentrations. Cinnamaldehyde also exhibited weak in vitro mutagenicity in the Ames test, although no in vivo mutagenicity was detected in mice up to 1000 mg/kg bw/day	QS inhibition; membrane destabilization	May complement probiotic quorum-quenching by inhibiting pathogen virulence and biofilm-associated signaling; however, direct probiotic synergy remains to be confirmed	[166,213,214,215,216,217,218,219,220]
*Curcuma longa* (turmeric)/curcumin	*S*. *aureus*, *E. coli*, *Pseudomonas aeruginosa*	The MIC values for pure curcumin vary widely, typically ranging from 7.8 to 5000 µg/mL. Curcumin exhibits strain-dependent antibacterial activity, which is generally stronger against Gram-positive bacteria, such as *S. aureus*, and the MIC values vary substantially between strains and experimental systems. Generally recognized as safe (GRAS) status. Oral doses of up to 8–12 g/day have been reported to be well tolerated in clinical studies; however, mild gastrointestinal adverse effects (nausea, diarrhea, abdominal discomfort, and dyspepsia) may occur at higher doses (>4 g/day). In vitro, cytotoxic effects, including cell cycle arrest and apoptosis, have been observed at high micromolar concentrations	Disrupts membrane integrity; inhibits FtsZ; suppresses quorum sensing; anti-inflammatory activity	May complement probiotic antimicrobial activity through membrane sensitization and QS/biofilm suppression; direct probiotic–turmeric synergy has been reported against *Cutibacterium acnes*, but further validation is needed in gut-relevant models	[117,118,133,221,222,223]
*Glycyrrhiza glabra* (licorice)/glycyrrhizin	*Helicobacter pylori*, *S. aureus*	In a combined anti-*H. pylori* model, 3 µg/mL *G. glabra* extract together with *Lacticaseibacillus paracasei* HP7 and *Perilla frutescens* extract reduced *H. pylori* growth, adhesion to AGS gastric epithelial cells, virulence gene expression, and colonization in mice. The antimicrobial activity of *G. glabra* extract has also been reported in vitro, although the MIC values vary depending on the extract type, test organism, and assay conditions. The major adverse effects of licorice and glycyrrhizin are hypertension, hypokalemia, and secondary electrolyte-related disorders. Acute toxicity studies have reported LD50 values ranging from 412 to 12,700 mg/kg for glycyrrhizin salts. Caution should be exercised during pregnancy	Enzyme inhibition; anti-inflammatory activity	May complement probiotic-mediated mucosal protection, particularly in anti-*H. pylori* and anti-inflammatory contexts, however, direct licorice–probiotic synergy remains insufficiently validated	[224,225,226,227,228,229,230,231,232]
*Lannea barteri*/flavonoids, saponins, tannins	Clinical isolates of bacteria and fungi	Crude aqueous and ethanolic *L. barteri* extracts, along with *Senna alata* and *Ricinus communis* extracts, exhibited antibacterial activity against wound- and skin-associated pathogens, including *S. aureus*, *P. aeruginosa*, *Klebsiella pneumoniae*, and *E. coli*. The MIC values for the tested plant extracts ranged from 3.13 to 12.50 mg/mL, whereas the MBC values ranged from 200 to 400 mg/mL. The extract combinations were further evaluated using FICI-based interaction analysis. Low acute oral toxicity; oral LD50 > 2500 mg/kg bw in mice. Transient weakness, inactivity and shivering were observed at 1500–2500 mg/kg without mortality	The antibacterial activity is likely related to tannins, saponins, and phenolic constituents; however, direct mechanistic validation of membrane permeabilization or protein denaturation by *L. barteri* remains limited	May complement probiotic-derived antimicrobial metabolites through independent antibacterial mechanisms; direct *Lannea*–probiotic synergy remains to be demonstrated	[233,234,235]
*Origanum vulgare* (oregano)/carvacrol, thymol	*P. aeruginosa*, *Listeria monocytogenes*, *Salmonella* spp.	Carvacrol exhibited antibacterial activity, with reported MIC values ranging from 0.005 to 0.04 mg/mL against selected foodborne/pathogenic bacteria, primarily attributed to carvacrol and thymol. Thymol exhibited antimicrobial activity, with MIC values ranging from 30–250 μg/mL against selected Gram-positive bacteria, 60–4000 μg/mL against Gram-negative bacteria. No mortality at 2000 mg/kg bw and a NOAEL of 200 mg/kg bw/day were reported for oregano essential oil. As a constituent of oregano essential oil, thymol contributes to the toxicological profile of the mixture. Carvacrol showed an oral LD50 of ~810 mg/kg and was well tolerated in humans at 1–2 mg/kg/day. Genotoxic effects have been reported in vitro at 460 μM. Oregano oil may irritate the skin, eyes, and respiratory mucosa.	Disrupts lipid membranes; interferes with ATP synthesis	May complement probiotic antimicrobial metabolites through membrane disruption and metabolic stress; however, direct oregano–probiotic synergy remains to be confirmed	[115,116,236,237,238,239,240,241,242,243]
*Punica granatum* (pomegranate)/ellagitannins, punicalagin	*S. aureus*, *Salmonella enterica*, *Clostridioides difficile*, *E. coli*, *Streptococcus mutans*, *Candida albicans*	Pomegranate peel extracts exhibited a concentration-dependent antimicrobial activity against *E. coli* (2.0–12.5 mg/mL), *S. mutans* (0.032–2.0 mg/mL), *S. enterica*, *S. aureus* (0.19–2.0 mg/mL), and *C. albicans* (0.125–1.0 mg/mL). MIC and MBC values have been reported for aqueous, ethanolic, and methanolic extracts, with the methanolic extract generally showing the lowest MIC/MBC values. Pomegranate extracts were also tested against β-lactamase-producing *E. coli*, confirming their activity against resistant Gram-negative isolates. Low toxicity; acute oral LD50 > 5000 mg/kg bw and NOAEL = 600 mg/kg bw/day for standardized pomegranate extract. No adverse effects were reported in humans receiving 1420 mg/day for 28 days	Protein precipitation; enzyme inhibition; antioxidant activity	Gut microbiota can biotransform pomegranate ellagitannins into bioactive urolithins, and probiotic-based strategies may support this metabolic pathway depending on the strain and host metabotype	[121,122,190,244,245,246,247,248,249]
*Ricinus communis*/ricinoleic acid	Gram-positive and Gram-negative pathogens	*R. communis* leaf extracts exhibited concentration- and solvent-dependent antimicrobial activity. MIC values ranging from 3.13 to 25.00 mg/mL and MBC/MFC values ranging from 12.50 to 200.00 mg/mL against selected bacterial and fungal pathogens. MIC and MBC values of 25–200 µg/mL and 25–400 µg/mL, respectively, for solvent fractions of *R. communis* leaf extract against *Bacillus* pathogens isolated from bovine mastitis. Ricin D is the principal toxic constituent of *R. communis*. The aqueous seed extract showed an estimated oral LD50 of 50–300 mg/kg bw, with mortality observed at 300 mg/kg bw, whereas the ethanolic seed extract exhibited an LD50 of 1100 mg/kg, with mortality beginning at 2000 mg/kg. Repeated administration (50–150 mg/kg bw/day) induced histopathological alterations in the liver and kidneys	Biofilm inhibition; membrane destabilization	May complement probiotic-derived anti-biofilm mechanisms, but direct probiotic–*R. communis* synergy remains to be demonstrated	[250,251,252,253,254,255,256]
*Rosmarinus officinalis* (rosemary)/rosmarinic acid, carnosic acid	*Bacillus* spp., *Staphylococcus* spp., *E. coli*, *Enterococcus faecalis*, *Candida*	Rosemary extracts showed antibacterial and anti-biofilm activity against clinically relevant pathogens, including UTI-associated isolates. The general MIC profile for rosmarinic acid ranges from 0.002 mg/mL to over 1.0 mg/mL for bacteria and from 0.1 mg/mL to 1.28 mg/mL for fungi of the genus *Candida.* Carnosic acid exhibited antimicrobial activity, with MIC values ranging from 2–5 μg/mL against selected Gram-positive bacteria, and effectively inhibited the QS activity of *S. aureus* at concentrations as low as 5 μM. Methanol extracts of *R. officinalis* (rich in carnosic acid ~30% and carnosol): The MIC ranges from 2 to 15 mg/mL for Gram-positive bacteria and from 2 to 60 mg/mL for Gram-negative bacteria. Rosemary extracts showed no genotoxicity in Ames and in vitro micronucleus assays. In a 90-day rat study, the NOAEL was 563 mg/kg bw/day, corresponding to 65 mg carnosic acid/kg bw/day and 7.65 mg carnosol/kg bw/day. JECFA established a temporary ADI of 0–0.3 mg/kg bw for rosemary extract, expressed as carnosic acid and carnosol. Rosemary tinctures were considered safe in feed at 500 mg/kg complete feed, although they may irritate the skin and eyes and act as dermal/respiratory sensitizers	Dual antioxidant + antimicrobial activity; metal chelation; QS activity	May support functional fermented/probiotic matrices while contributing antimicrobial and anti-biofilm activity against selected pathogens; however, direct probiotic–rosemary synergy requires further validation	[257,258,259,260,261,262,263,264,265,266]
*Senna alata*/anthraquinones	*S. aureus*, *Streptococcus pyogenes*, *E. coli*, *Pseudomonas* spp. and fungi	An ethanol/methanol extract of *S. alata* was effective at concentrations ranging from 0.313 to 6.25 mg/mL against *S. aureus*, at 0.483 mg/mL against *S. pyogenes*, and at concentrations ranging from 0.625 to 12.5 mg/mL against *E. coli*. The ethanol extract was effective in the range of 1.25–5.60 mg/mL against *Candida albicans*, 3.50 mg/mL against *Aspergillus niger*, and 9.80 mg/mL against *Trichophyton mentagrophytes.* *S. alata* leaf extracts have been reported to have low toxicity. The oral LD50 was >2000 mg/kg bw. Repeated administration at 500–3000 mg/kg bw/day for 15–28 days caused no significant changes in hematological, biochemical, or histopathological parameters. Mild hepatic steatosis and slight hepatocyte edema were observed at 2000 mg/kg bw/day after 28-day exposure	DNA intercalation; oxidative stress	May complement probiotic-derived organic acids and other metabolites through independent redox- and metabolism-disrupting effects; direct probiotic–Senna synergy remains to be demonstrated	[267,268,269,270,271,272,273,274,275]
*Vitis vinifera* (grape seed)/proanthocyanidins	Enteric pathogens	Procyanidin-rich grape seed extract exhibits antibacterial and antivirulence activities against Gram-negative pathogens. An MIC50 of 44.17 µg/mL was reported against *Salmonella* Typhimurium and demonstrated the inhibition of biofilm formation by *E. coli* and *S.* Typhimurium. Other studies support the barrier-protective and microbiota-modulating effects of grape seed proanthocyanidins but do not provide classical MIC values for antimicrobial activity. *V. vinifera* extracts have low toxicity. An aqueous leaf extract showed an oral LD50 of 2828.43 mg/kg bw, and repeated administration (250–1000 mg/kg bw/day for 28 days) caused no marked hepatic or renal toxicity, although mild histopathological alterations were observed at higher doses. In vitro, a proanthocyanidin-rich grape seed extract showed no significant cytotoxicity toward Caco-2 cells after 24 h of exposure at concentrations ranging from 3.13 to 50 μg/mL	Inhibition of adhesion, EPS formation, barrier-protective and microbiota-modulating effects of grape seed proanthocyanidins	May complement probiotic-derived anti-biofilm mechanisms, as grape seed procyanidins inhibit pathogen adhesion/biofilm formation, whereas probiotic EPS can independently disrupt pathogen biofilms. However, direct grape seed–probiotic synergy requires further investigation	[119,120,276,277,278]
*Zingiber officinale* (ginger)/gingerols, shogaols	*E. coli*, *Shigella*, *Streptococcus* spp.	Ginger extracts exhibit extract- and strain-dependent antibacterial activities. Ginger extract inhibited selected *E. coli* strains with reported MIC/MBC values of 625 µg/mL, whereas no inhibition was observed for an AcrAB-TolC-positive *E. coli* strain, even at 10 mg/mL, indicating strain-dependent resistance. The red ginger ethanol extract also showed MIC values of 125 and 500 µg/mL against *E. coli* and *S. aureus*, respectively. No treatment-related adverse effects were observed in a 90-day oral toxicity study in rats administered ginger essential oil, and a NOAEL of 500 mg/kg bw/d was established. The EFSA also retained a NOAEL of 11 mg/kg bw/day for gingerols and shogaols based on a 35-day rat study	Anti-adhesion effects; inhibition of virulence factors	May support probiotic growth and microbiota modulation, but direct evidence of ginger-mediated enhancement of probiotic coaggregation remains limited	[279,280,281,282,283,284,285]

Abbreviations: ADI, acceptable daily intake; bw, body weight; CBD, cannabidiol; CBG, cannabigerol; EPS, extracellular polymeric substances; GRAS, Generally Recognized As Safe; JECFA, Joint Food and Agriculture Organization of the United Nations/ World Health Organization Expert Committee on Food Additives; LD50, Median Lethal Dose; MBC, minimum bactericidal concentration; MDR, multidrug-resistant; MIC, minimum inhibitory concentration; MSSA, methicillin-sensitive *Staphylococcus aureus*; MRSA, methicillin-resistant *S. aureus*; NOAEL, No-observed-adverse-effect level; ROS, reactive oxygen species; UTI, urinary tract infection; QS, quorum sensing. Note: Interaction types were categorized as quantitative synergy, additive effect, complementary interaction, or proposed mechanism according to the original study design and reported outcomes.

**Table 2:** Comparison of probiotic versus phytochemical mechanisms relevant to antimicrobial activity and synergy.

Feature	Probiotics (Live Microorganisms)	Phytochemicals (Plant-Derived Compounds)	Evidence-Based Interpretation of Interaction	References
Primary nature	Living cells with dynamic metabolism and replication	Small molecules or complex mixtures, chemically characterizable after extraction	Hypothesized complementary interaction: Selected living probiotics can continuously produce metabolites that interact with relatively stable phytochemical compounds	[289,290,291]
Main antimicrobial mechanisms	Production of organic acids, bacteriocins, biosurfactants, hydrogen peroxide; competitive exclusion; biofilm disruption; coaggregation with pathogens	Membrane disruption; enzyme inhibition and inhibition of DNA replication/transcription; quorum-sensing interference; inhibition of virulence factors; oxidative stress induction	Additive/complementary evidence: Different antimicrobial targets may support multi-site pathogen inhibition; quantitative synergy requires confirmation by FICI, checkerboard, or time-kill assays	[292,293]
Host-related effects	Modulation of immune responses (innate and adaptive); enhancement of epithelial barrier integrity; restoration of microbiota balance	Anti-inflammatory, antioxidant, and immunomodulatory effects; protection of epithelial cells and tight junctions	Complementary evidence: The combined effects may support epithelial barrier reinforcement and immune regulation, although the outcomes are strain-, compound-, and model-dependent	[294,295]
Prebiotic/metabolic interactions	Utilize dietary substrates and plant compounds; biotransform phytochemicals into new metabolites (e.g., phenolic acids)	Serve as substrates or modulators of probiotic metabolism; can increase SCFA and bacteriocin production	Documented complementary evidence: Plant bioactives may stimulate probiotic metabolism, while selected probiotics can biotransform phytochemicals into more bioactive metabolites	[296,297]
Specificity of activity	Often strain-specific; effects vary widely between strains of the same species	Compound- and dose-dependent; mixture effects can be broad but less strain-specific	Hypothesized/rational design criterion: strain identity, phytochemical composition, and dose must be matched to maximize the probability of synergy	[192,298]
Stability and formulation	Sensitive to temperature, pH, oxygen, processing, and storage conditions	Generally, more stable than live cells but some are light/heat/oxidation-sensitive	Formulation-dependent interactions: Co-encapsulation may improve delivery and stability, but compatibility must be experimentally validated	[299,300,301]
Risk of resistance development	Lower risk than conventional antibiotics; act via ecological and competitive mechanisms	Multi-target actions reduce but do not eliminate the risk of resistance	Hypothesized benefit: Multi-target actions may reduce the selective pressure on single bacterial targets; however, this requires validation in long-term in vivo models	[302,303]
Regulatory status	Often classified as foods, supplements, or live biotherapeutic products; strain-level safety assessment required	Regulated as botanical extracts, supplements, or drugs depending on their use and purity	Translational consideration: Combined products require separate assessments of probiotic strain safety, phytochemical characterization, and formulation stability	[304,305]
Advantages	Potentially self-renewing, adaptable, microbiota-restoring; transient persistence or colonization possible	Rapid, direct antimicrobial effect; chemically standardizable; easy to dose	Complementary rationale: Rapid phytochemical activity may be combined with the longer-term ecological and immunomodulatory effects of probiotics	[306]
Limitations	Variable colonization success; survival through the GI tract not guaranteed; strain selection critical	Variability in plant composition; bioavailability issues; potential toxicity at high doses	Critical interpretation: Synergy cannot be assumed; antagonism, reduced probiotic viability, poor bioavailability, or dose-dependent toxicity may occur	[172,307,308]

Abbreviations: FICI, fractional inhibitory concentration index; GI, gastrointestinal tract; SCFA, short-chain fatty acid. Explanation: Interaction categories are interpreted as documented quantitative synergy, additive/complementary evidence, hypothesized mechanisms, or formulation-dependent interactions according to the type of evidence available in the cited literature. Quantitative synergy should be reserved for studies using formal synergy assessments, such as FICI, checkerboard, or time-kill assays.

**Table 3:** Selected studies reporting synergistic or complementary interactions between probiotics and plant-derived bioactives against pathogens.

Combination of Probiotic Strain(s)/Microbial Component	Plant-Derived Bioactive/Extract	Synergy Assessment Method	Target Pathogen(s)	Reported Interaction Outcome	References
*Lactiplantibacillus plantarum* Zs2058	Eugenol, the main bioactive compound of clove (*Syzygium aromaticum*)	In vitro antibacterial assay, HT-29 epithelial cell model, and *Salmonella*-infected C57BL/6 mouse model	*Salmonella* Typhimurium SL1344	Combined treatment reduced *Salmonella* growth, invasion, virulence gene expression, and inflammatory cytokine responses more effectively than individual interventions	[328]
Human intestinal *Lactobacillus* spp., including *L. plantarum* NG6 and *L. paracasei* DB3	Garlic extract	In vitro synbiotic antibacterial assay; SEM validation	*Salmonella* Typhi and *S.* Typhimurium	Combination with 12.5% garlic extract inhibited *S.* Typhi and *S.* Typhimurium while maintaining *Lactobacillus* viability; SEM confirmed pathogen cell damage.	[359,360]
*Lacticaseibacillus rhamnosus*	Red raspberry (*Rubus idaeus*) pomace and seed preparations with ellagitannins	Co-culture assay	EHEC, *S.* Typhimurium, *Salmonella* Enteritidis, *Listeria monocytogenes*	Combined use of this phytoprobiotic enhanced pathogen suppression compared with raspberry preparations alone, including the reduction or elimination of selected *Salmonella* strains and reduced *L. monocytogenes* counts.	[361]
*L. plantarum*	*Lawsonia inermis* extract	In vitro antimicrobial and anti-inflammatory evaluation	*Staphylococcus aureus*	The combination exhibited synergistic antimicrobial activity against *S. aureus* and reduced IL-6 and TNF-α levels to levels similar to those observed in the uninfected control group	[362]
*L. plantarum* CICC 6253	Tea polyphenols	Mono-culture and co-culture growth assays; fermented sausage model	*S. aureus* and *E. coli*	Tea polyphenols exhibited concentration-dependent dual-directional regulation: promoting *L. plantarum* growth while suppressing pathogens at appropriate concentrations (e.g., 2 mg/mL) by acting as a prebiotic, enhancing its proliferation and dominance in co-culture systems	[320]
*L. plantarum* 299v, *L. rhamnosus* GG (ATCC 53103), *L. rhamnosus* ATCC 7469 and *Saccharomyces cerevisiae var. boulardii* HANSEN CBS 5926	Polyphenol-rich extracts from *Gentiana asclepiadea*, *Hypericum perforatum*, *Satureja montana*, and *Achillea millefolium*	In vitro growth modulation assay	Opportunistic intestinal bacteria and yeast	Plant extracts selectively modulated probiotic and pathogenic microorganisms, supporting complementary gut-targeted interactions rather than formally quantified synergy. Plant extracts stimulated probiotic yeast growth along with the suppression of *Candida* spp.	[144]

Abbreviations: EHEC, enterohemorrhagic *Escherichia coli*; SEM, scanning electron microscopy.

**Table 4:** Methodological approaches used to evaluate probiotic–phytochemical interactions.

Methodological Category	Method/Model	Main Purpose	Key Endpoints/Outputs	Interpretation/Limitations	References
In vitro antimicrobial synergy testing	Checkerboard microdilution assay	Quantitative evaluation of combined antimicrobial activity of two agents	Fractional inhibitory concentration index (FICI): FICI ≤ 0.5, synergy; 0.5 < FICI ≤ 1, additive effect; 1 < FICI ≤ 4, indifference; FICI > 4, antagonism	Adaptable to probiotic cell-free supernatants, probiotic cell suspensions, plant extracts alone, or plant extracts combined with probiotic metabolites	[293]
	Time-kill curves	Assessment of the kinetics of antimicrobial interactions over time	Change in viable counts over 24–48 h; synergy typically defined as ≥2 log_10_ CFU/mL reduction compared with the most active single treatment	Captures dynamic killing effects; identifies delayed or time-dependent synergy; distinguishes bacteriostatic from bactericidal activity	[367,368]
	Agar diffusion and co-culture inhibition assays	Visual and semi-quantitative assessment of inhibition between probiotics and phytochemicals	Inhibition zone enhancement or interference; pathogen growth suppression in co-culture	Includes well diffusion, spot-on-lawn, and double-layer assays; useful for visualizing interactions, especially when probiotics and pathogens physically interact	[369,370]
Biofilm-related synergy assays	Microtiter biofilm formation and disruption tests	Evaluation of anti-biofilm activity of single agents and combinations	Biofilm biomass (Crystal Violet), metabolic activity (XTT, resazurin), total DNA or EPS content	Useful for showing whether combinations inhibit biofilm formation or eradicate established biofilms more effectively than individual treatments	[371,372,373,374,375,376]
	Confocal laser scanning microscopy (CLSM)	Structural visualization of biofilm-related synergistic effects	Biofilm architecture, membrane integrity (LIVE/DEAD staining), EPS disruption, cell clustering, coaggregation	Reveals structural synergy not detectable in bulk assays; especially valuable for mechanistic interpretation	[377,378,379]
Probiotic functional and metabolic assays	Growth stimulation and prebiotic activity tests	Determination of whether plant extracts stimulate probiotic proliferation	Optical density growth curves, colony-forming unit (CFU) counts, fermentation endpoint analyses	Indicates prebiotic or metabolic synergy when plant compounds promote probiotic growth or activity	[380,381,382,383]
	Metabolomics and profiling of bioactive metabolites	Characterization of metabolites involved in synergistic interactions	LC-MS/MS, GC-MS, or NMR-based quantification of SCFAs, bacteriocins, biosurfactants, reuterin, and phenolic metabolites	Demonstrates whether plant extracts stimulate probiotic metabolite production or whether probiotics biotransform phytochemicals into more active compounds	[384,385,386,387,388]
Immunological synergy assays	Epithelial cell line models (e.g., Caco-2, HT-29, IEC-6)	Assessment of barrier-protective and anti-inflammatory effects of combinations	Tight junction proteins (ZO-1, claudins, occludin), cytokines (IL-6, IL-1β, TNF-α), ROS production, pathogen translocation	Useful for evaluating whether combinations strengthen epithelial barrier function and reduce inflammatory damage	[389]
	Macrophage/dendritic cell models	Evaluation of immune-modulating effects of probiotic–phytochemical treatments	Phagocytosis, nitric oxide production, TLR signaling modulation, antimicrobial peptide expression	Reveals synergistic effects on innate immune responses and immunomodulation	[390,391,392]
Genomic and transcriptomic approaches	RNA-seq and qPCR	Identification of gene expression changes induced by combined treatment	Downregulation of virulence genes, upregulation of stress-response genes, modulation of quorum-sensing pathways, induction of probiotic metabolic pathways	Provides mechanistic insight into molecular pathways underlying synergy	[393,394]
	Whole-genome sequencing (WGS) of pathogens	Monitoring of genomic adaptations under combined treatment pressure	Resistance-associated mutations, adaptive stress responses	Helps distinguish mechanisms of synergistic versus antagonistic interactions	[395,396]
In vivo and ex vivo synergy models	Animal models	Evaluation of probiotic–phytochemical synergy under physiologically relevant conditions	Pathogen burden in tissues, gut microbiota composition, systemic immune responses, intestinal permeability, protection in infection models	Provides translational relevance and validates synergistic effects beyond in vitro systems	[307,397]
	Ex vivo human-derived models	Mechanistic testing in systems approximating the human gut environment; Fecal fermentation systems (e.g., SHIME, TIM-2), human intestinal organoids	Changes in microbiota composition, SCFA production, microbial metabolic activity, epithelial barrier integrity, cytokine responses, and metabolomic profiles	Enables physiologically relevant evaluation of microbiome-level and host–microbe interactions under controlled conditions	[398,399,400]
Computational and systems biology approaches	Network modeling and molecular docking	Prediction of mechanistic interactions between phytochemicals, probiotics, and pathogen targets	Binding interactions, pathway interference, quorum-sensing-related targets	Supports hypothesis generation and mechanistic interpretation of synergistic interactions	[401,402]
	Machine learning and predictive synergy modeling	Identification of combinations with high probability of synergy	AI-driven integration of multi-omics and experimental datasets	Emerging approach for rational design of synbiotic and probiotic–phytochemical formulations	[401,403,404]
Standardization and interpretation	Multi-assay framework for synergy interpretation	Improvement of reproducibility and confidence in synergy classification	At least two independent synergy assays recommended, ideally checkerboard plus time-kill, combined with biofilm assays and metabolomic or transcriptomic validation	Reduces overinterpretation; increases robustness of conclusions; helps distinguish true synergy from additive or independent effects	[130,363,366]

Abbreviations: AI, artificial intelligence; CFU, colony-forming unit; CLSM, confocal laser scanning microscopy; EPS, extracellular polymeric substances; FICI, fractional inhibitory concentration index; GC-MS, gas chromatography–mass spectrometry; LC-MS/MS, liquid chromatography–tandem mass spectrometry; NMR, nuclear magnetic resonance; qPCR, quantitative polymerase chain reaction; RNA-seq, RNA sequencing; ROS, reactive oxygen species; SCFAs, short-chain fatty acids; SHIME, Simulator of the Human Intestinal Microbial Ecosystem; TIM-2, TNO in vitro model of the colon; TLR, Toll-like receptor; WGS, whole-genome sequencing; XTT, 2,3-bis(2-methoxy-4-nitro-5-sulfophenyl)-2H-tetrazolium-5-carboxanilide; ZO-1, zonula occludens-1.

**Table 5:** Selected human and translational evidence for combined probiotic–plant bioactive interventions. Only studies published within the predefined search period (January 2016–April 2026) were included in this table.

Condition/Population	Probiotic	Plant-Derived Bioactives	Study Design	Main Outcome	Limitations	References
Pediatric irritable bowel syndrome (IBS)	Synbiotic/probiotic intervention: *Bifidobacterium lactis* B94, 5 × 10^9^ CFU	Prebiotic inulin, 900 mg	Randomized, double-blind, controlled, and prospective clinical trial; *n* = 71 children between the ages of 4 and 16 years divided for 3 groups: synbiotic, probiotic and prebiotic; 2 daily for 4-week intervention	Synbiotic and probiotic treatments improved initial IBS complaints compared with prebiotic treatment; the synbiotic group showed a higher full recovery rate than the prebiotic group (39.1% vs. 12.5%, respectively)	Pediatric and condition-specific cohort; short-term symptom-based outcomes; synbiotic formulation was not based on a defined plant extract or isolated phytochemical; no formal synergy metrics such as FICI or time-kill assays.	[408]
Symptomatic COVID-19/post-infectious symptom burden (post-COVID-19 syndrome)	*Lactobacillus*-based probiotic/prebiotic (synbiotic) capsule containing *L. plantarum*, *L. rhamnosus*, *L. bulgaricus*, *Lactococcus lactis*, *L. paracasei*, 10 × 10^9^ CFU; with prebiotic inulin, 200 mg	Phytochemical-rich whole food capsule: *Citrus sinensis* fruit, 400 mg from 200 mg of 2:1 extract, standardized to contain 70 mg of bioflavonoids; *Chamomile/Matricaria recutita* (flower), 1000 mg from 22 mg of 10:1 extract and 65 mg of 12:1 extract; *Curcuma longa rhizome* in curcumin complex, 1600 mg of curcumin from 25 mg of 64:1 extract, standardized to contain 23.8 mg of curcuminoid; *Punica granatum* (rinds and seeds), 1000 mg from 25 mg of 40:1 extract, standardized to contain 10 mg of ellagic acid; *Polygonum cuspidatum* root containing 100 mg of resveratrol	Randomized, double-blind, placebo-controlled trial; *n* = 147 adults; UK Phyto-V Study; participants received one phytochemical-rich capsule or placebo 2 daily, in addition to a synbiotic capsule, for 30 days.	Addition of phytochemical-rich capsules to synbiotic supplementation improved fatigue, cough, and overall well-being scores; GI symptoms improved in many participants reporting baseline GI complaints	Protocol was amended so most participants received probiotic/prebiotic supplementation; not a full factorial design; outcomes were symptom-based; spontaneous recovery possible	[409]
Mild to moderate facial acne	Sachet blend, containing probiotic compounds: *Bifidobacterium breve* BR03 DSM 16604, ≥0.5 × 10^9^ CFU; *Lacticaseibacillus casei* LC03 DSM 27537, ≥0.5 × 10^9^ CFU; *Ligilactobacillus salivarius* LS03 DSM 22776, ≥1.0 × 10^9^ CFU; combined dose of ≥2 × 10^9^ CFU	Botanical extract as sachet blend, containing lupeol from *Solanum melongena* and *Echinacea* extract (exact botanical dose not reported by authors)	Monocentric, randomized, double-blind, four-arm, placebo-controlled study; *n* = 114 adults; 1 sachet daily for 8 weeks	Significant reduction in inflammatory lesions, erythema, desquamation, sebum secretion, and *Cutibacterium acnes* abundance; strongest effect observed with combined probiotic–botanical intervention.	Exact dose of botanical extracts not reported; single-center study; relatively short intervention period; no formal quantitative synergy assessment	[410]
Low-risk prostate cancer under active surveillance/older men with untreated early prostate cancer; related analyses from the same randomized trial/cohort	Five-blend 10^9^ CFU *Lactobacillus* probiotic/prebiotic (synbiotic) capsule: *L. rhamnosus* 300 CFU, 5.6 mg; *L. plantarum* 500 CFU, 5.6 mg; *L. paracasei* 300 CFU, 835 µg; *L. bulgaricus* 50 CFU, 100 mg; *Lactococcus lactis* 200 CFU, 835 µg; combined dose of 10^9^ CFU; prebiotic inulin 90%, 100 mg; and vitamin D, 2.2 mg (500 IU);	Phytochemical-rich supplement (PRS) containing broccoli (*Brassica oleracea* 150 mg); *Curcuma longa* (150 mg and 50:1 extract), standardized to curcuminoids 95% 500 mg; pomegranate (*Punica granatum* 150 mg and 50:1 extract), standardized to 90% ellagic acid (500 mg); green tea (*Camellia sinens* is 3:1 extract), standardized to 45% epigallocatechin-3-gallate (EGCG; 150 mg); ginger (*Zingiber officinale* Roscoe 5 mg); and cranberry (*Vaccinium* subg. *oxycoccus*, 25:1 extract, 100 mg)	Phase II randomized, placebo-controlled trial; *n* = 208 evaluable men; all received phytochemical-rich supplement (2 capsules daily for 4 months) and were randomized to probiotic or placebo for 4 months	Phytochemical-rich supplementation was associated with slower prostate-specific antigen (PSA) progression, and addition of *Lactobacillus* probiotic further improved PSA dynamics, urinary symptoms, erectile function, and inflammatory markers. PRS + probiotic improved grip strength more than PRS + placebo, reduced NLR, and was associated with higher testosterone levels at 4 months. In the prostate cancer analysis from the same trial, PRS + probiotic capsules were also associated with more favorable PSA dynamics, urinary symptoms, and erectile function	Disease-specific cohort of older men under active surveillance for prostate cancer; no placebo-only arm; baseline imbalances between groups; reliance on surrogate biomarkers (including PSA); limited duration of follow-up; and the need for independent replication in a broader population	[411,412,413]

Abbreviation: CFU, colony-forming unit; EGCG, epigallocatechin gallate; FICI, fractional inhibitory concentration index; GI, gastrointestinal; IBS, irritable bowel syndrome; IU, international units; PRS, phytochemical-rich supplement; PSA, prostate-specific antigen.

**Table 6:** Research gaps and technological needs in probiotic–phytochemical antimicrobial development.

Area	Key Research Gaps	Impact on Synergy Assessment	Technological and Methodological Needs	Selected References
Plant extract variability	Heterogeneous phytochemical composition due to source, processing, and extraction methods	Leads to inconsistent antimicrobial and synergy outcomes across studies	Standardized extraction; LC-MS/MS and NMR fingerprinting; validated chemical markers	[107,468,469]
Strain-specific probiotic responses	Strong dependence on strain identity, genome, metabolism, and stress tolerance	Limits reproducibility and generalization of probiotic–phytochemical interactions	Strain-level characterization; WGS; phenotype–genotype mapping; high-throughput screening	[478,479]
Lack of standardized synergy protocols	Variability in inoculum size, media, extraction solvents, endpoints (MIC vs. biofilm vs. CFU), and interpretation thresholds	Hampers comparison between studies and increases the risk of overinterpretation of synergy claims	Harmonized protocols; combined use of checkerboard and time-kill assays; inclusion of biofilm and multi-endpoint analyses	[470]
Limited in vivo and clinical validation	Predominance of in vitro data; limited animal and human studies; unclear bioavailability and microbiota interactions	Reduces translational relevance and clinical confidence in synergistic efficacy	Animal models; ex vivo gut systems; randomized clinical trials; PK/PD studies	[289,480]
Co-formulation and stability challenges	Probiotic sensitivity (pH, oxygen, temperature); phytochemical instability; antagonistic interactions; mismatched release profiles	May reduce efficacy or negate synergistic effects in real formulations	Co-encapsulation; microgels; controlled-release systems; stability optimization	[481,482,483]
Microbiota-level effects	Limited understanding of community-wide and resistome-level responses	Overlooks ecological consequences and long-term effects of combined interventions	Metagenomics, metabolomics, and network analysis; longitudinal microbiome studies	[484,485,486]
Biofilm and quorum-sensing models	Lack of standardized multi-species and physiologically relevant models	Underestimates physiologically relevant pathogen behavior and synergy effects	Multispecies biofilm systems; CLSM imaging; QS reporter assays; microfluidic models	[487,488]
Regulatory and safety framework	Unclear classification of combined probiotic–phytochemical products	Complicates clinical translation and commercialization	Strain-level safety assessment; toxicity evaluation; regulatory harmonization	[489,490]
Data integration and prediction	Fragmented datasets and lack of predictive models	Limits rational design of effective combinations	Multi-omics integration; machine learning; predictive synergy modeling	[491,492,493]

Abbreviations: CLSM, confocal laser scanning microscopy; CFU, colony-forming unit; LC-MS/MS, liquid chromatography–tandem mass spectrometry; NMR, nuclear magnetic resonance; QS, quorum sensing; WGS, whole-genome sequencing; PK/PD, pharmacokinetics/pharmacodynamics.

## Data Availability

No new data were created or analyzed in this study.

## Abbreviations

The following abbreviations are used in this manuscript:

3-HPA	3-hydroxypropionaldehyde
ADI	Acceptable daily intake
AI	Autoinducer
AMPs	Antimicrobial peptides
AMR	Antimicrobial resistance
bw	Body weight
CBD	Cannabidiol
CBG	Cannabigerol
CBGA	Cannabigerolic acid
CFU	Colony-forming unit
CLSM	Confocal laser scanning microscopy
DC	Dendritic cell
DSS	Dextran sulfate sodium
EFSA	European Food Safety Authority
EGCG	Epigallocatechin gallate
EHEC	Enterohemorrhagic *Escherichia coli*
ETEC	Enterotoxigenic *Escherichia coli*
EPS	Extracellular polymeric substances
FDA	Food and Drug Administration
FICI	Fractional inhibitory concentration index
FOS	Fructooligosaccharides
FtsZ	Filamenting temperature-sensitive mutant Z
GC-MS	Gas chromatography–mass spectrometry
GI	Gastrointestinal tract
GOS	Galacto-oligosaccharides
GRAS	Generally recognized as safe
HDAC	Histone deacetylase
IL-6	Interleukin 6
IU	International units
JECFA	Joint Food and Agriculture Organization/World Health Organization Expert Committee on Food Additives
LAB	Lactic acid bacteria
LC-MS/MS	Liquid chromatography–tandem mass spectrometry
LD50	Median Lethal Dose
LPS	Lipopolysaccharide
LUB	Lactate-utilizing bacterium
MAMPs	Microbial-associated molecular patterns
MAPK	Mitogen-activated protein kinase
MBC	Minimum bactericidal concentration
MDR	Multidrug-resistant
MIC	Minimum inhibitory concentration
MLCK	Myosin light-chain kinase
MSSA	Methicillin-sensitive *Staphylococcus aureus*
MRSA	Methicillin-resistant *Staphylococcus aureus*
NF-κB	Nuclear factor kappa-light-chain-enhancer of activated B cells
NMR	Nuclear magnetic resonance
NOAEL	No-observed-adverse-effect level
Nrf2	Nuclear factor erythroid 2-related factor 2 (Nrf2)
PK/PD	Pharmacokinetics/pharmacodynamics
PRS	Phytochemical-rich supplement
PSA	Prostate-specific antigen
qPCR	Quantitative polymerase chain reaction
QS	Quorum sensing
RCT	Randomized controlled trial
ROS	Reactive oxygen species
SCFAs	Short-chain fatty acids
SEM	Scanning electron microscopy
SHIME	Simulator of the Human Intestinal Microbial Ecosystem
*s.l.*	*Sensu lato*
TJ	Tight junction
TLR	Toll-like receptor
TNF-α	Tumor necrosis factor-alpha
UTI	Urinary tract infection
WGS	Whole-genome sequencing
XTT	2,3-bis(2-methoxy-4-nitro-5-sulfophenyl)-2H-tetrazolium-5-carboxanilide
ZO-1	Zonula occludens-1

## References

[1] Langford B.J., Wrona F.J., Hardcastle L., Wojcik K.M. (2025). Beyond the Echo Chamber: Reframing AMR Awareness Efforts to Reach the Other 99.9%. Antimicrob. Steward. Heal. Epidemiol..

[2] Lawani-Luwaji E., Nkweke F. (2026). Public Awareness as a Strategic Tool for Combating Antibiotic Resistance in Low- and Middle-Income Countries: Evidence from Nigeria. Discov. Public Health.

[3] Saputra I.W.A.G.M., Putri W.C.W.S., Lesmana C.B.J., Budayanti N.N.S., Wirawan I.M.A. (2024). Community Engagement-Related Intervention to Address Antimicrobial Resistance (AMR) across Human, Animal and Environmental Health: A Systematic Review. Univers. J. Public Health.

[4] Pandey S., Dhawan K., Murali S., Altaf A., Rexwal Y., Gupta A., Mahato D.K., Kamle M., Kumar P. (2026). Probiotics, Prebiotics, and Synbiotics as Functional Foods. Handbook of Functional Foods.

[5] Dhami N., Gangwar M., Kumar D., Rao A.K., Kumar S., Gangwar M., Nath G. (2024). Beyond Antibiotics: Pioneering Strategies in Infection Control to Counter Antibiotic Resistance’s Rising Tide. Emerging Paradigms for Antibiotic-Resistant Infections: Beyond the Pill.

[6] Jhuma T.A., Dey S.S., Sarkar R., Siddique S., Moniruzzaman M., Chowdhury A. (2025). Biofilm Inhibition and Antagonism of *Klebsiella Pneumoniae* by Probiotic Lactic Acid Bacteria (LAB) Isolated from Raw Cow Milk. Microb. Pathog..

[7] Vieira Sabino Y.N., De Almeida T.C., Faria C.A., Da Cunha Rezende S.D., Costa Miranda J.P., Paiva A.D., Machado A.B.F. (2025). Antivirulence Effects of Lactic Acid Bacteria: Pioneering New Probiotic Applications. Benef. Microbes.

[8] Mishra B., Mishra A.K., Mohanta Y.K., Yadavalli R., Agrawal D.C., Reddy H.P., Gorrepati R., Reddy C.N., Mandal S.K., Shamim M.Z. (2024). Postbiotics: The New Horizons of Microbial Functional Bioactive Compounds in Food Preservation and Security. Food Prod. Process. Nutr..

[9] Bocchio F., Mancabelli L., Milani C., Lugli G.A., Tarracchini C., Longhi G., Conto F.D., Turroni F., Ventura M. (2024). Compendium of *Bifidobacterium*-Based Probiotics: Characteristics and Therapeutic Impact on Human Diseases. Microbiome Res. Rep..

[10] Rabetafika H.N., Razafindralambo A., Ebenso B., Razafindralambo H.L. (2023). Probiotics as Antibiotic Alternatives for Human and Animal Applications. Encyclopedia.

[11] Elshaghabee F.M.F., Rokana N., Gulhane R.D., Sharma C., Panwar H. (2017). *Bacillus* As Potential Probiotics: Status, Concerns, and Future Perspectives. Front. Microbiol..

[12] Sharma A., Lee H.-J. (2025). Antimicrobial Activity of Probiotic Bacteria Isolated from Plants: A Review. Foods.

[13] Massip C., Branchu P., Bossuet-Greif N., Chagneau C.V., Gaillard D., Martin P., Boury M., Sécher T., Dubois D., Nougayrède J.-P. (2019). Deciphering the Interplay between the Genotoxic and Probiotic Activities of *Escherichia Coli* Nissle 1917. PLoS Pathog..

[14] Ali S.S., Al-Tohamy R., Al-Zahrani M., Badr A., Sun J. (2026). Essential Oils and Plant-Derived Bioactive Compounds: A Comprehensive Review of Their Therapeutic Potential, Mechanisms of Action, and Advances in Extraction Technologies. Phytochem. Rev..

[15] Bouyahya A., Bakri Y., Atta-ur-Rahman (2020). Anti-Inflammatory and Immunomodulatory Properties of Medicinal Plant Products. Frontiers in Clinical Drug Research—Anti Allergy Agents.

[16] Coimbra A., Gallardo E., Luís Â., Gaspar P.D., Ferreira S., Duarte A.P. (2025). Bioactive Potential of Wild Plants from Gardunha Mountain: Phytochemical Characterization and Biological Activities. Molecules.

[17] Da Silva L.E., Confortin C., Swamy M.K., Pal D., Nayak A.K. (2021). Antibacterial and Antifungal Plant Metabolites from the Tropical Medicinal Plants. Bioactive Natural Products for Pharmaceutical Applications.

[18] França F., De Souza J.C., O’Connor P.M., Matos A.P., Pimentel-Filho N.D.J. (2026). Plant Antimicrobials: Extraction, Characterization and Activity against Foodborne Microorganisms. Folia Microbiol..

[19] Verma P.K., Verma S., Pandey N., Chakrabarty D., Upadhyay S.K., Singh S.P. (2021). Antimicrobial Products from Plant Biodiversity. Bioprospecting of Plant Biodiversity for Industrial Molecules.

[20] Gościniak A., Sip A., Szulc P., Cielecka-Piontek J. (2025). Bifunctional Systems of *Amelanchier Alnifolia* Leaves Extract-Oligosaccharides with Prebiotic and Antidiabetic Benefits. Molecules.

[21] Aydın B. (2025). Exploring the Multifaceted Health Benefits of *Tripolium pannonicum*: Antimicrobial, Antioxidant, and Prebiotic Properties With Phytochemical Insights. Chem. Biodivers..

[22] Reza M.A., Hossain M.A., Lee S.-J., Kim J.-C., Park S.-C. (2016). In Vitro Prebiotic Effects and Quantitative Analysis of *Bulnesia Sarmienti* Extract. J. Food Drug Anal..

[23] Chaudhari A., Dwivedi M.K. (2022). The Concept of Probiotics, Prebiotics, Postbiotics, Synbiotics, Nutribiotics, and Pharmabiotics. Probiotics in the Prevention and Management of Human Diseases.

[24] Abdelhamid S.M., Darwish W.S., Tantawy E.A., Shaheen M.N., El Dairouty R.K. (2025). Rosemary Plantarum Kariesh Cheese: Phytochemical, Microbiological, Virology Activities and Sensory Evaluations. Egypt. J. Chem..

[25] Oulahal N., Degraeve P. (2022). Phenolic-Rich Plant Extracts With Antimicrobial Activity: An Alternative to Food Preservatives and Biocides?. Front. Microbiol..

[26] Wishna-Kadawarage R.N., Jensen M., Powałowski S., Hickey R.M., Siwek M. (2023). In-Vitro Screening of Compatible Synbiotics and (Introducing) “Prophybiotics” as a Tool to Improve Gut Health. Int. Microbiol..

[27] Savelyeva L.N., Bondarchuk M.L., Nekrasova O.S., Bazaron B.Z., Dashinimaev B.T. (2026). Efficacy of a New Natural Remedy in the Treatment of Calves with Gastrointestinal Diseases Accompanied by Diarrhea. Sib. Her. Sci..

[28] Aleman R.S., Yadav A. (2023). Systematic Review of Probiotics and Their Potential for Developing Functional Nondairy Foods. Appl. Microbiol..

[29] Czarnecka N., Jankowska M., Nawrot S., Nogal-Nowak K., Wąsik S., Czerwonka G. (2025). Phenotypic and Genetic Characterization and Production Abilities of *Lacticaseibacillus rhamnosus* Strain 484—A New Probiotic Strain Isolated From Human Breast Milk. Food Sci. Nutr..

[30] Matejčeková Z., Čmiková T., Ačai P., Valík Ľ. (2025). Temperature-Dependent Growth Kinetics of *Lacticaseibacillus rhamnosus* GG in Oat-Based Milk Alternative. Acta Chim. Slovaca.

[31] Popova-Krumova P., Danova S., Atanasova N., Yankov D. (2024). Lactic Acid Production by *Lactiplantibacillus Plantarum* AC 11S—Kinetics and Modeling. Microorganisms.

[32] Calvigioni M., Bertolini A., Codini S., Mazzantini D., Panattoni A., Massimino M., Celandroni F., Zucchi R., Saba A., Ghelardi E. (2023). HPLC-MS-MS Quantification of Short-Chain Fatty Acids Actively Secreted by Probiotic Strains. Front. Microbiol..

[33] Hagihara M., Yamashita R., Matsumoto A., Mori T., Kuroki Y., Kudo H., Oka K., Takahashi M., Nonogaki T., Yamagishi Y. (2018). The Impact of *Clostridium Butyricum* MIYAIRI 588 on the Murine Gut Microbiome and Colonic Tissue. Anaerobe.

[34] Patiño L.A., Fuentes C.M., Ochoa O.I., Estela-Zape J.L. (2025). Metabolic Acidosis Due to D-Lactate in a Patient with Intestinal Resection: Diagnostic Challenges and Nutritional Strategies. Int. J. Surg. Case Rep..

[35] Honda S., Eguchi H., Okino Y., Wang D.-S. (2025). The Probiotic Strain *Clostridium Butyricum* TO-A Produces Butyrate by Utilizing Lactate and Acetate. Int. J. Mol. Sci..

[36] Dos Santos Freitas A., Da Silva Fernandes L.J., Coelho-Rocha N.D., De Jesus L.C.L., De Rezende Rodovalho V., Da Silva T.F., De Oliveira Carvalho R.D., Azevedo V. (2022). Immunomodulatory and Antiinflammatory Mechanisms of Probiotics. Probiotics.

[37] Silva Y.P., Bernardi A., Frozza R.L. (2020). The Role of Short-Chain Fatty Acids From Gut Microbiota in Gut-Brain Communication. Front. Endocrinol..

[38] Wu G., Wang R., Wang Y., Sun S., Chen J., Zhang Q. (2025). Crosstalk Among Gut Microbiota, Microbial Metabolites, and Inflammatory Cytokines: Current Understanding and Future Directions. Foods.

[39] Manoharan M., Balasubramaniam T.S. (2022). An Extensive Review on Production, Purification, and Bioactive Application of Different Classes of Bacteriocin. J. Trop. Biodivers. Biotechnol..

[40] Suda S., Field D., Barron N., Walls D., Loughran S.T. (2017). Antimicrobial Peptide Production and Purification. Protein Chromatography.

[41] Bédard F., Biron E. (2018). Recent Progress in the Chemical Synthesis of Class II and S-Glycosylated Bacteriocins. Front. Microbiol..

[42] Kuniyoshi T.M., O’Connor P.M., Lawton E., Thapa D., Mesa-Pereira B., Abulu S., Hill C., Ross R.P., Oliveira R.P.S., Cotter P.D. (2022). An Oxidation Resistant Pediocin PA-1 Derivative and Penocin A Display Effective Anti- *Listeria* Activity in a Model Human Gut Environment. Gut Microbes.

[43] García-Márquez J., Tapia-Paniagua S., Moriñigo M.Á., Arijo S., Austin B., Sharifuzzaman S.M. (2022). Probiotics for Controlling Infectious Diseases. Probiotics in Aquaculture.

[44] Mihaylova-Garnizova R., Davidova S., Hodzhev Y., Satchanska G. (2024). Antimicrobial Peptides Derived from Bacteria: Classification, Sources, and Mechanism of Action against Multidrug-Resistant Bacteria. Int. J. Mol. Sci..

[45] Shleeva M.O., Kondratieva D.A., Kaprelyants A.S. (2023). *Bacillus Licheniformis*: A Producer of Antimicrobial Substances, Including Antimycobacterials, Which Are Feasible for Medical Applications. Pharmaceutics.

[46] Antoshina D.V., Balandin S.V., Ovchinnikova T.V. (2022). Structural Features, Mechanisms of Action, and Prospects for Practical Application of Class II Bacteriocins. Biochemistry.

[47] Anjana, Tiwari S.K. (2022). Bacteriocin-Producing Probiotic Lactic Acid Bacteria in Controlling Dysbiosis of the Gut Microbiota. Front. Cell. Infect. Microbiol..

[48] Bengtsson T., Selegård R., Musa A., Hultenby K., Utterström J., Sivlér P., Skog M., Nayeri F., Hellmark B., Söderquist B. (2020). Plantaricin NC8 Aβ Exerts Potent Antimicrobial Activity against *Staphylococcus* Spp. and Enhances the Effects of Antibiotics. Sci. Rep..

[49] Al-Hazmi N.E. (2025). Antibacterial and Anticancer Activities of Rhizobacterial Lipopeptides against Carcinogenic Bacteria. Antonie Van. Leeuwenhoek.

[50] Cossus L., Roux-Dalvai F., Kelly I., Nguyen T.T.A., Antoun H., Droit A., Tweddell R.J. (2021). Interactions with Plant Pathogens Influence Lipopeptides Production and Antimicrobial Activity of *Bacillus Subtilis* Strain PTB185. Biol. Control.

[51] Saiyam D., Dubey A., Malla M.A., Kumar A. (2024). Lipopeptides from *Bacillus*: Unveiling Biotechnological Prospects—Sources, Properties, and Diverse Applications. Braz. J. Microbiol..

[52] Monteagudo-Mera A., Rastall R.A., Gibson G.R., Charalampopoulos D., Chatzifragkou A. (2019). Adhesion Mechanisms Mediated by Probiotics and Prebiotics and Their Potential Impact on Human Health. Appl. Microbiol. Biotechnol..

[53] Rana A. (2026). Smriti Probiotics: Mechanism of Action and Gastrointestinal Health: Gut Guardians: Unlocking the Power of Probiotics. J. Sci. Food Agric..

[54] Gorreja F., Walker W.A. (2022). The Potential Role of Adherence Factors in Probiotic Function in the Gastrointestinal Tract of Adults and Pediatrics: A Narrative Review of Experimental and Human Studies. Gut Microbes.

[55] Horrocks V., King O.G., Yip A.Y.G., Marques I.M., McDonald J.A.K. (2023). Role of the Gut Microbiota in Nutrient Competition and Protection against Intestinal Pathogen Colonization. Microbiology.

[56] Dang Y., Sun Y., Zhou Y., Men X., Wang B., Li B., Ren Y. (2022). Effects of Probiotics on Growth, the Toll-like Receptor Mediated Immune Response and Susceptibility to *Aeromonas Salmonicida* Infection in Rainbow Trout Oncorhynchus Mykiss. Aquaculture.

[57] Mansilla F., Takagi M., Garcia-Castillo V., Aso H., Nader-Macias M.E., Vignolo G., Kitazawa H., Villena J. (2020). Modulation of Toll-like Receptor-Mediated Innate Immunity in Bovine Intestinal Epithelial Cells by Lactic Acid Bacteria Isolated from Feedlot Cattle. Benef. Microbes.

[58] Azad A.K., Sarker M., Wan D. (2018). Immunomodulatory Effects of Probiotics on Cytokine Profiles. BioMed Res. Int..

[59] Rastin M., Mahmoudi M., Tabasi N., Kia N., Hajavi J., Esmaeili S. (2023). The Evaluation of the Effect of Tolerogenic Probiotics on the Maturation of Healthy Dendritic Cells versus Immature Dendritic Cells. Iran. J. Immunol..

[60] Rostoll Cangiano L., Villot C., Amorin-Hegedus R., Malmuthuge N., Gruninger R., Guan L.L., Steele M. (2023). Saccharomyces Cerevisiae Boulardii Accelerates Intestinal Microbiota Maturation and Is Correlated with Increased Secretory IgA Production in Neonatal Dairy Calves. Front. Microbiol..

[61] Vale G.C., Mota B.I.S., Ando-Suguimoto E.S., Mayer M.P.A. (2024). Lactobacilli Probiotics Modulate Antibacterial Response Gene Transcription of Dendritic Cells Challenged with LPS. Probiotics Antimicrob. Proteins.

[62] Zhao M., Liang X., Meng Y., Lu H., Lin K., Gong P., Liu T., Yi H., Pan J., Zhang Y. (2024). Probiotics Induce Intestinal IgA Secretion in Weanling Mice Potentially through Promoting Intestinal APRIL Expression and Modulating the Gut Microbiota Composition. Food Funct..

[63] Orlando A., Maqoud F., Mallardi D., Drago S., Malerba E., Chimienti G., Russo F. (2025). *Lactobacillus Rhamnosus* GG and *Lactobacillus Paracasei* IMPC2.1 Mitigate LPS-Induced Epithelial Barrier Dysfunction: A Focus on Autophagy Regulation. Int. J. Mol. Sci..

[64] Di Vito R., Conte C., Traina G. (2022). A Multi-Strain Probiotic Formulation Improves Intestinal Barrier Function by the Modulation of Tight and Adherent Junction Proteins. Cells.

[65] Wang G., Xu Q., Jin X., Hang F., Liu Z., Zhao J., Zhang H., Chen W. (2018). Effects of *Lactobacilli* with Different Regulatory Behaviours on Tight Junctions in Mice with Dextran Sodium Sulphate-Induced Colitis. J. Funct. Foods.

[66] Zhang Y., Anderson R.C., You C., Purba A., Yan M., Maclean P., Liu Z., Ulluwishewa D. (2024). Lactiplantibacillus Plantarum ST-III and *Lacticaseibacillus Rhamnosus* KF7 Enhance the Intestinal Epithelial Barrier in a Dual-Environment In Vitro Co-Culture Model. Microorganisms.

[67] Vinayamohan P., Joseph D., Viju L.S., Baskaran S.A., Venkitanarayanan K. (2024). Efficacy of Probiotics in Reducing Pathogenic Potential of Infectious Agents. Fermentation.

[68] Markowska K., Szymanek-Majchrzak K., Pituch H., Majewska A. (2024). Understanding Quorum-Sensing and Biofilm Forming in Anaerobic Bacterial Communities. Int. J. Mol. Sci..

[69] Salman M.K., Abuqwider J., Mauriello G. (2023). Anti-Quorum Sensing Activity of Probiotics: The Mechanism and Role in Food and Gut Health. Microorganisms.

[70] Yan J., Bassler B.L. (2019). Surviving as a Community: Antibiotic Tolerance and Persistence in Bacterial Biofilms. Cell Host Microbe.

[71] Carvalho F.M., Mergulhão F.J.M., Gomes L.C. (2021). Using Lactobacilli to Fight *Escherichia Coli* and *Staphylococcus Aureus* Biofilms on Urinary Tract Devices. Antibiotics.

[72] Zhang C., Wang C., Xiu Z. (2021). Regulation of C-Di-GMP in Biofilm Formation of *Klebsiella Pneumoniae* in Response to Antibiotics and Probiotic Supernatant in a Chemostat System. Curr. Microbiol..

[73] Erega A., Stefanic P., Dogsa I., Danevčič T., Simunovic K., Klančnik A., Smole Možina S., Mandic Mulec I. (2021). Bacillaene Mediates the Inhibitory Effect of *Bacillus Subtilis* on *Campylobacter Jejuni* Biofilms. Appl. Environ. Microbiol..

[74] Sarwar A., Brader G., Corretto E., Aleti G., Abaidullah M., Sessitsch A., Hafeez F.Y. (2018). Qualitative Analysis of Biosurfactants from *Bacillus* Species Exhibiting Antifungal Activity. PLoS ONE.

[75] Grevanny R., Rizal M.F., Suharsini M. (2026). Effect of Limosilactobacillus Reuteri Probiotic on Dual-Species Biofilms of *Streptococcus Sobrinus* and *Candida Albicans*: An in Vitro Study. Saudi Dent. J..

[76] Lee M.-K., Chen I.-H., Hsu I.-L., Tsai W.-H., Lee T.-Y., Jhong J.-H., Liu B.-C., Huang T.-Y., Lin F.-K., Chang W.-W. (2024). The Impact of *Lacticaseibacillus Paracasei* GMNL-143 Toothpaste on Gingivitis and Oral Microbiota in Adults: A Randomized, Double-Blind, Crossover, Placebo-Controlled Trial. BMC Oral Health.

[77] Afonso A.C., Saavedra M.J., Simões M., Simões L.C. (2025). The Role of the Proteosurfaceome and Exoproteome in Bacterial Coaggregation. Biotechnol. Adv..

[78] Afonso A.C., Botting J., Gomes I.B., Saavedra M.J., Simões L.C., Liu J., Simões M. (2024). Elucidating Bacterial Coaggregation through a Physicochemical and Imaging Surface Characterization. Sci. Total Environ..

[79] Golletz P., Jensen S.D., Collignon M., Hall C., Khamas A.B., Møllebjerg A., Schlafer S., Meyer R.L., Tykwinska K. (2025). Coaggregation of Oral Pathogens by Postbiotic *Lactobacilli*. J. Oral Microbiol..

[80] Rashad Hameed S., Abdul Sattar Salman J. (2023). Co-Aggregative Effect of Probiotics Bacteria against Diarrheal Causative Bacteria. Arch. Razi Inst..

[81] Malfa P., Brambilla L., Giardina S., Masciarelli M., Squarzanti D.F., Carlomagno F., Meloni M. (2023). Evaluation of Antimicrobial, Antiadhesive and Co-Aggregation Activity of a Multi-Strain Probiotic Composition against Different Urogenital Pathogens. Int. J. Mol. Sci..

[82] Aydoğdu N.S., Özdemir N., Taş T.K. (2025). Functional Burrata Cheese Enriched with *Lacticaseibacillus Casei* ATCC 393: Insights into Production, Unique Characteristics, and Aromatic Profile. Mljekarstvo.

[83] Elshaghabee F.M.F., El-Hussein A., Mohamed M.S.M. (2022). Enhancement of Labneh Quality by Laser-Induced Modulation of *Lactocaseibacillus Casei* NRRL B-1922. Fermentation.

[84] Dalal K.S., Patil S.P., Pendharkar G.B., Dalal D.S., Chaudhari B.L., Verma P. (2023). Reuterin: A Broad Spectrum Antimicrobial Agent and Its Applications. Industrial Microbiology and Biotechnology.

[85] Zavišić G., Ristić S., Petričević S., Janković D., Petković B. (2024). Microbial Contamination of Food: Probiotics and Postbiotics as Potential Biopreservatives. Foods.

[86] Chen L., Bromberger P.D., Nieuwenhuiys G., Hatti-Kaul R. (2016). Redox Balance in *Lactobacillus Reuteri* DSM20016: Roles of Iron-Dependent Alcohol Dehydrogenases in Glucose/ Glycerol Metabolism. PLoS ONE.

[87] Petrariu O.-A., Barbu I.C., Niculescu A.-G., Constantin M., Grigore G.A., Cristian R.-E., Mihaescu G., Vrancianu C.O. (2024). Role of Probiotics in Managing Various Human Diseases, from Oral Pathology to Cancer and Gastrointestinal Diseases. Front. Microbiol..

[88] Ghorani M. (2022). Antiviral Effects of Probiotic Metabolites. Iran. J. Med. Microbiol..

[89] İspirli H., Öztürk H.İ., Dertli E. (2025). Characterization and in Situ Bioprotective Efficacy of Reuterin E81 Produced by *Limosilactobacillus Reuteri* E81 in White Cheese Model. Food Biosci..

[90] Niamah A.K., Mohammed A.A., Alhelf N.A. (2023). Antibacterial Activity and Identification of Produced Reuterin from Local *Lactobacillus Reuteri* LBIQ1 Isolate. J. microbiol. Biotechnol. Food Sci..

[91] Ortiz-Rivera Y., Sánchez-Vega R., Gutiérrez-Méndez N., León-Félix J., Acosta-Muñiz C., Sepulveda D.R. (2017). Production of Reuterin in a Fermented Milk Product by *Lactobacillus Reuteri*: Inhibition of Pathogens, Spoilage Microorganisms, and Lactic Acid Bacteria. J. Dairy Sci..

[92] Fernández-Cruz M.L., Martín-Cabrejas I., Pérez-del Palacio J., Gaya P., Díaz-Navarro C., Navas J.M., Medina M., Arqués J.L. (2016). In Vitro Toxicity of Reuterin, a Potential Food Biopreservative. Food Chem. Toxicol..

[93] Soltani S., Couture F., Boutin Y., Ben Said L., Cashman-Kadri S., Subirade M., Biron E., Fliss I. (2021). In Vitro Investigation of Gastrointestinal Stability and Toxicity of 3-Hyrdoxypropionaldehyde (Reuterin) Produced by *Lactobacillus Reuteri*. Toxicol. Rep..

[94] Efenberger-Szmechtyk M., Nowak A., Czyzowska A. (2021). Plant Extracts Rich in Polyphenols: Antibacterial Agents and Natural Preservatives for Meat and Meat Products. Crit. Rev. Food Sci. Nutr..

[95] Piekarska-Radzik L., Klewicka E. (2021). Mutual Influence of Polyphenols and *Lactobacillus* Spp. Bacteria in Food: A Review. Eur. Food Res. Technol..

[96] Boubker A., El Ouardi A., El Kamli T., El Hamidi A., Kaicer M., Kichou F., Ameur N., Errafii K., Ben Aakame R., Sifou A. (2025). Phytochemical Analysis, Antioxidant and Antibacterial Activities, Minerals Element Profiling, and Identification of Bioactive Compounds by UPLC-HRMS Orbitrap in Four Aromatic and Medicinal Plants. Molecules.

[97] Awad A.M., Kumar P., Ismail-Fitry M.R., Jusoh S., Ab Aziz M.F., Sazili A.Q. (2021). Green Extraction of Bioactive Compounds from Plant Biomass and Their Application in Meat as Natural Antioxidant. Antioxidants.

[98] El-Saadony M.T., Saad A.M., Mohammed D.M., Alkafaas S.S., Abd El-Mageed T.A., Fahmy M.A., Ezzat Ahmed A., Algopishi U.B., Abu-Elsaoud A.M., Mosa W.F.A. (2025). Plant Bioactive Compounds: Extraction, Biological Activities, Immunological, Nutritional Aspects, Food Application, and Human Health Benefits—A Comprehensive Review. Front. Nutr..

[99] Flores-López M.L., Guía-García J.L., López-Romero J.C., Torres-Moreno H., Moo-Huchin V.M., García-Munguía A.M., Charles-Rodríguez A.V. (2024). *Rhus Microphylla* Leaves Extracts Obtained by Ohmic Heating: Physicochemical Composition and Bioactive Properties. Ind. Crops Prod..

[100] Mohammed S.J., Mohammed A.S., Ghafoor D.D., Najmuldeen H.H., Tamar A.I., Jalal D.D., Alghofaili F., Kader D.A., Hamarawf R.F., Kayani K.F. (2026). Sustainable Solutions to Antibiotic Resistance: A Comprehensive Review of Plant-Derived Carbon Dots and Phytochemicals as Innovative Antibacterial Agents. Phytochem. Rev..

[101] Suganya T., Packiavathy I.A.S.V., Aseervatham G.S.B., Carmona A., Rashmi V., Mariappan S., Devi N.R., Ananth D.A. (2022). Tackling Multiple-Drug-Resistant Bacteria With Conventional and Complex Phytochemicals. Front. Cell. Infect. Microbiol..

[102] Sarker M.A.R., Ahn Y.-H. (2022). Photodynamic Inactivation of Multidrug-Resistant Bacteria in Wastewater Effluent Using Green Phytochemicals as a Natural Photosensitizer. Environ. Pollut..

[103] Bengag A., Metlef S., Zidane A., Sadoud M. (2026). Phytochemical Screening and Bactericidal Activity of Three Algerian Medicinal Plant Extracts against Nosocomial Multidrug-Resistant *Enterobacteria*. Not. Sci. Biol..

[104] Shanmugam Y., Chokkalingam D., Gopinath G.R., Moses A.C., Thanikachalam P., Ms P., Bharathy P. (2025). Plant-Based Phytochemicals as Antibiotic Alternatives for Gangrene: A Sustainable Approach to Infection Management. Sustain. Chem. Clim. Action.

[105] Das A., Ruhal R. (2025). Potential of Plants-Based Alkaloids, Terpenoids and Flavonoids as Antibacterial Agents: An Update. Process. Biochem..

[106] Fydrych D., Jeziurska J., Wełna J., Kwiecińska-Piróg J. (2025). Potential Use of Selected Natural Compounds with Anti-Biofilm Activity. Int. J. Mol. Sci..

[107] Alum E.U., Nwali B.U., Akwari A.A., Emeruwa A.P., Obasi D.C., Okoroh P.N., Aniokete U.C. (2026). Exploring Novel Phytochemicals as Strategies in the Fight Against Antimicrobial Resistance. Nat. Prod. Commun..

[108] Rasool S.A., Rasool M.S., Ajaz M., Mojgani N., Dadar M. (2021). Encountering the Antibiotic Resistance by Bioactive Components and Therapies: Probiotics, Phytochemicals and Phages. Probiotic Bacteria and Postbiotic Metabolites: Role in Animal and Human Health.

[109] Tan Z., Deng J., Ye Q., Zhang Z. (2022). The Antibacterial Activity of Natural-Derived Flavonoids. Curr. Top. Med. Chem..

[110] Mangal S., Singh V., Chhibber S., Harjai K. (2022). Natural Bioactives Versus Synthetic Antibiotics for the Attenuation of Quorum Sensing-Regulated Virulence Factors of *Pseudomonas aeruginosa*. Future Microbiol..

[111] Ivanov M., Novović K., Malešević M., Dinić M., Stojković D., Jovčić B., Soković M. (2022). Polyphenols as Inhibitors of Antibiotic Resistant Bacteria—Mechanisms Underlying Rutin Interference with Bacterial Virulence. Pharmaceuticals.

[112] Helcman M., Šmejkal K., Čulenová M., Béres T., Treml J. (2025). Natural Phenolics Disrupt Microbial Communication by Inhibiting Quorum Sensing. Microorganisms.

[113] Kitichalermkiat A., Katsuki M., Sato J., Sonoda T., Masuda Y., Honjoh K., Miyamoto T. (2020). Effect of Epigallocatechin Gallate on Gene Expression of *Staphylococcus Aureus*. J. Glob. Antimicrob. Resist..

[114] Feng C., Chen Z., Guan S., Li J., Qu M., Geng H. (2025). Formation Mechanism of Injured Bacteria after Disinfection with Epigallocatechin Gallate (EGCG) as a Disinfectant. J. Water Health.

[115] Moghrovyan A., Sahakyan N. (2024). Antimicrobial Activity and Mechanisms of Action of *Origanum vulgare* L. Essential Oil: Effects on Membrane-Associated Properties. AIMS Biophys..

[116] Wijesundara N.M., Lee S.F., Cheng Z., Davidson R., Rupasinghe H.P.V. (2021). Carvacrol Exhibits Rapid Bactericidal Activity against *Streptococcus Pyogenes* through Cell Membrane Damage. Sci. Rep..

[117] Lahiri D., Nag M., Dey S., Dutta B., Dash S., Ray R.R. (2021). Phytocompounds of *Curcuma Longa* Extract Are More Effective against Bacterial Biofilm than Pure Curcumin Only: An in-Vitro and in-Silico Analysis. Kuwait J. Sci..

[118] Adamczak A., Ożarowski M., Karpiński T.M. (2020). Curcumin, a Natural Antimicrobial Agent with Strain-Specific Activity. Pharmaceuticals.

[119] Nicolosi R.M., Bonincontro G., Imperia E., Badiali C., De Vita D., Sciubba F., Dugo L., Guarino M.P.L., Altomare A., Simonetti G. (2023). Protective Effect of Procyanidin-Rich Grape Seed Extract against Gram-Negative Virulence Factors. Antibiotics.

[120] Han M., Song P., Huang C., Rezaei A., Farrar S., Brown M.A., Ma X. (2016). Dietary Grape Seed Proanthocyanidins (GSPs) Improve Weaned Intestinal Microbiota and Mucosal Barrier Using a Piglet Model. Oncotarget.

[121] Alghamdi M.A., Al-Sarraj F., Alamshani W.H., Alotibi I., Al-Zahrani M., Albiheyri R., Nass N.M., Sajer B.H., Bataweel N.M., Al-Matary M.A. (2024). Antibacterial Power of Pomegranate Extracts against Beta-Lactamase Producing *Escherichia Coli*. Caryologia.

[122] Bagchi S., Tiwari N., Dutta S., Nanda M., Sengupta P. (2025). Study on the Antibacterial and Antioxidant Activities of *Punica Granatum* (Pomegranate) Peel Extracts. J. Integr. Sci. Technol..

[123] Alizadeh Behbahani B., Falah F., Lavi Arab F., Vasiee M., Tabatabaee Yazdi F. (2020). Chemical Composition and Antioxidant, Antimicrobial, and Antiproliferative Activities of *Cinnamomum zeylanicum* Bark Essential Oil. Evid. -Based Complement. Altern. Med..

[124] Wang Q.-Y., Zeng X.-A., Liu Z.-W., Brennan C.S. (2018). Variations in Cellular Membrane Fatty Acid Composition of *Escherichia Coli* in Resistance to Pulsed Electric Fields Induced by Eugenol. J. Food Process. Preserv..

[125] Połeć K., Wyżga B., Olechowska K., Hąc-Wydro K. (2022). On the Synergy/Antagonism of Selected Terpenes in the Effect on Lipid Membranes Studied in Model Systems. J. Mol. Liq..

[126] Bouyahya A., Chamkhi I., Balahbib A., Rebezov M., Shariati M.A., Wilairatana P., Mubarak M.S., Benali T., El Omari N. (2022). Mechanisms, Anti-Quorum-Sensing Actions, and Clinical Trials of Medicinal Plant Bioactive Compounds against Bacteria: A Comprehensive Review. Molecules.

[127] Barbieri R., Coppo E., Marchese A., Daglia M., Sobarzo-Sánchez E., Nabavi S.F., Nabavi S.M. (2017). Phytochemicals for Human Disease: An Update on Plant-Derived Compounds Antibacterial Activity. Microbiol. Res..

[128] Todorov S.D., De Almeida B.M., Lima E.M.F., Fabi J.P., Lajolo F.M., Hassimotto N.M.A. (2025). Phenolic Compounds and Bacteriocins: Mechanisms, Interactions, and Applications in Food Preservation and Safety. Mol. Nutr. Food Res..

[129] Mulat M., Banicod R.J.S., Tabassum N., Javaid A., Karthikeyan A., Jeong G.-J., Kim Y.-M., Jung W.-K., Khan F. (2025). Multiple Strategies for the Application of Medicinal Plant-Derived Bioactive Compounds in Controlling Microbial Biofilm and Virulence Properties. Antibiotics.

[130] Stewart P.S., White B., Boegli L., Hamerly T., Williamson K.S., Franklin M.J., Bothner B., James G.A., Fisher S., Vital-Lopez F.G. (2019). Conceptual Model of Biofilm Antibiotic Tolerance That Integrates Phenomena of Diffusion, Metabolism, Gene Expression, and Physiology. J. Bacteriol..

[131] Morão L.G., Polaquini C.R., Kopacz M., Torrezan G.S., Ayusso G.M., Dilarri G., Cavalca L.B., Zielińska A., Scheffers D., Regasini L.O. (2019). A Simplified Curcumin Targets the Membrane of *Bacillus subtilis*. MicrobiologyOpen.

[132] Fujimori M., Sogawa H., Ota S., Karpov P., Shulga S., Blume Y., Kurita N. (2018). Specific Interactions between Mycobacterial FtsZ Protein and Curcumin Derivatives: Molecular Docking and Ab Initio Molecular Simulations. Chem. Phys. Lett..

[133] Hussain Y., Alam W., Ullah H., Dacrema M., Daglia M., Khan H., Arciola C.R. (2022). Antimicrobial Potential of Curcumin: Therapeutic Potential and Challenges to Clinical Applications. Antibiotics.

[134] Soltani S., Shakeri A., Iranshahi M., Boozari M. (2021). A Review of the Phytochemistry and Antimicrobial Properties of *Origanum Vulgare* L. and Subspecies. Iran. J. Pharm. Res..

[135] Anniballi F., Purgatorio C., Serio A., Scalfaro C., Taglieri S., Paparella A. (2025). Gene Expression Dynamics in *Bacillus Cereus* and *Bacillus Subtilis* Treated with *Thymus Vulgaris* and *Origanum Vulgare* Subsp. *Hirtum* Essential Oils. Front. Microbiol..

[136] Singh H.C., Kom H.W., Wankhar W., Loying S., Sarma M.P., Kumar R., Saikia D. (2025). Probiotics and Postbiotics: Exploring Their Role as Antibiotic Alternatives and Health Enhancers. J. Nat. Rem..

[137] Ahmed S.A.K.S., Rudden M., Smyth T.J., Dooley J.S.G., Marchant R., Banat I.M. (2019). Natural Quorum Sensing Inhibitors Effectively Downregulate Gene Expression of *Pseudomonas Aeruginosa* Virulence Factors. Appl. Microbiol. Biotechnol..

[138] Pun M., Galsurker O., Khazanov N., Charkowski A., Yelin S., Kerem Z., Weitman M., Senderowitz H., Yedidia I. (2025). Multimodal Inhibition of *Pectobacterium brasiliense* Virulence by the *Citrus* Flavanone Naringenin. J. Agric. Food Chem..

[139] Warrier A., Satyamoorthy K., Murali T.S. (2025). Naringenin as a Potent Natural Biofilm Inhibitor of *Pseudomonas Aeruginosa* in Diabetic Foot Ulcers Through lasR Competitive Inhibition. Curr. Microbiol..

[140] Joshi J.R., Khazanov N., Senderowitz H., Burdman S., Lipsky A., Yedidia I. (2016). Plant Phenolic Volatiles Inhibit Quorum Sensing in *Pectobacteria* and Reduce Their Virulence by Potential Binding to ExpI and ExpR Proteins. Sci. Rep..

[141] Pati S., Antara N.S., Gunam I.B.W., Sarkar T., Lahiri D. (2024). Elucidating the Quorum Sensing Inhibitory Mechanism of Flavonoid Quercetin by Molecular Docking, Molecular Dynamics Simulation, and MM-GBSA Study. Lett. Appl. Nanobiosci..

[142] Ashaolu T.J. (2020). Immune Boosting Functional Foods and Their Mechanisms: A Critical Evaluation of Probiotics and Prebiotics. Biomed. Pharmacother..

[143] Savino F., Guandalini S., Dhawan A. (2022). Prebiotics in Pediatrics. Textbook of Pediatric Gastroenterology, Hepatology and Nutrition.

[144] Milutinović M., Dimitrijević-Branković S., Rajilić-Stojanović M. (2021). Plant Extracts Rich in Polyphenols as Potent Modulators in the Growth of Probiotic and Pathogenic Intestinal Microorganisms. Front. Nutr..

[145] Vidal-Casanella O., Núñez O., Granados M., Saurina J., Sentellas S. (2021). Analytical Methods for Exploring Nutraceuticals Based on Phenolic Acids and Polyphenols. Appl. Sci..

[146] Jun B.-G., Kim S.-H., Kim S.-H., Hong S.-M., Lee H., Lim Y., Kim S.-Y., Lee C.-H. (2024). Metabolomic Comparison of Guava (*Psidium guajava* L.) Leaf Extracts Fermented by *Limosilactobacillus Fermentum* and *Lactiplantibacillus Plantarum* and Their Antioxidant and Antiglycation Activities. Nutrients.

[147] Koh Y.-C., Pan M.-H. (2026). Pterostilbene-Mediated Microbiota Shifts: Implications and Opportunities. J. Tradit. Complement. Med..

[148] Huang X., Jiang F., Chen X., Xian Y. (2024). Plant-Derived Polysaccharides Benefit Weaned Piglets by Regulating Intestinal Microbiota: A Review. J. Agric. Food Chem..

[149] Mitra K., Bhattacharya D., Banerjee J., Nag M., Lahiri D. (2026). Phytochemicals and Gut Health: Modulating Microbiota and Promoting Digestive Wellness. Phytoceuticals in Food for Health and Wellness.

[150] Tinrat S., Chomnawang M.T. (2024). Exploring Local Edible Plants as Potential Prebiotic Sources for Their Synbiotic Applications. LWT.

[151] Thirumala Reddy G., Shashikala A.R. (2026). Study of the Prebiotic Potential of *Punica Granatum* (Pomegranate) Flower Extract on the Bioactivity of Probiotic Strains. Res. J. Biotechnol..

[152] Lau L.Y.J., Huang K., Quek S.Y. (2025). Unlocking the Potential of Leaf Extracts in Promoting Probiotic Growth. Food Biosci..

[153] Gkalpinos V.K., Anagnostou V.A., Mitropoulou G., Kompoura V., Karapantzou I., Fasoulis C.K., Vasdekis E.P., Kourkoutas Y., Tzakos A.G. (2023). *Aloysia Citrodora* Extracts Cultivated in Greece as Antioxidants and Potent Regulators of Food Microbiota. Appl. Sci..

[154] Liu X., Su S., Yao J., Zhang X., Wu Z., Jia L., Liu L., Hou R., Farag M.A., Liu L. (2024). Research Advance about Plant Polysaccharide Prebiotics, Benefit for Probiotics on Gut Homeostasis Modulation. Food Biosci..

[155] Tang C., Li D., Xie B., Li Y., Sun Z. (2024). Synergistic Effect of B-Type *Lotus* Seedpod Oligomeric Procyanidin and Probiotics against Adhesion of Enterotoxigenic *Escherichia Coli* In Vitro. J. Food Biochem..

[156] Zhang N., Jin M., Wang K., Zhang Z., Shah N.P., Wei H. (2022). Functional Oligosaccharide Fermentation in the Gut: Improving Intestinal Health and Its Determinant Factors-A Review. Carbohydr. Polym..

[157] Zúñiga M., Yebra M.J., Monedero V. (2021). Complex Oligosaccharide Utilization Pathways in *Lactobacillus*. Curr. Issues Mol. Biol..

[158] Pázmándi K., Szöllősi A.G., Fekete T. (2024). The “Root” Causes behind the Anti-Inflammatory Actions of Ginger Compounds in Immune Cells. Front. Immunol..

[159] Mbogho Abogo J., Sima Obiang C., Begouabe H., Ngoua Meye Misso R.L., Orango Bourdette J.O., Ndong Atome G.R., Obame Engonga L.C., Ondo J.P. (2024). Evaluation of the Efficacy of Medicinal Plants Based on Immunological Biomarkers in the Treatment of Bacterial Infections: Current Status and Future Directions. Gene Rep..

[160] Nittayananta W., Rezaei N., Ziaei H. (2026). Immunomodulatory Effects of Medicinal Plants. Oral Immunology.

[161] Appiah E.K., Fatsi P.S.K., Magna E.K., Saito H., Omura M., Kawai K. (2024). Immunomodulatory Effects of Extract on Innate Immune Responses in Infected With. Microbe.

[162] Mishra S.K., Ishfaq P.M., Tripathi S., Gupta N., Sangwan N.S., Farag M.A., Modolo L.V. (2022). Fruits as Boosters of the Immune System. Plants and Phytomolecules for Immunomodulation.

[163] Milad S.S., Ali S.E., Attia M.Z., Khattab M.S., EL-Ashaal E.S., Elshoky H.A., Azouz A.M. (2023). Enhanced Immune Responses in Dexamethasone Immunosuppressed Male Rats Supplemented with Herbal Extracts, Chitosan Nanoparticles, and Their Conjugates. Int. J. Biol. Macromol..

[164] Na-Phatthalung P., Teles M., Voravuthikunchai S.P., Tort L., Fierro-Castro C. (2018). Immune-Related Gene Expression and Physiological Responses in Rainbow Trout (*Oncorhynchus mykiss*) after Intraperitoneal Administration of *Rhodomyrtus Tomentosa* Leaf Extract: A Potent Phytoimmunostimulant. Fish Shellfish Immunol..

[165] De Santis F., Poerio N., Gismondi A., Nanni V., Di Marco G., Nisini R., Thaller M.C., Canini A., Fraziano M. (2019). Hydroalcoholic Extract from *Origanum Vulgare* Induces a Combined Anti-Mycobacterial and Anti-Inflammatory Response in Innate Immune Cells. PLoS ONE.

[166] Singh N., Yadav S.S., Kumar S., Narasihman B., Ramasamy K., Lim S.M., Shah S.A.A. (2025). Synthesis, Characterization, in-Vitro and in-Silico Therapeutic Studies of Cinnamaldehyde Derivatives. J. Mol. Struct..

[167] Mukherjee B., Al Hoque A., Hota S.H., Gope S., Ray M., Barman M., Bhattacharya S., Chakraborty S., Das L., Mukherjee B. (2025). Antioxidants and Their Physiological Role in Free Radical Scavenging. Dietary Supplements and Nutraceuticals.

[168] Kaur G., Gautam S., Arora P., Dhingra N. (2022). Estimation of Synergistic Antioxidant Effect of Methanolic Extracts of Some Medicinal Herbs. Res. J. Pharm. Technol..

[169] Wu Y.-R., Chen J.-S., Chen L.-C., Chen L., Huang Y.-F., Liao C.-S. (2025). Synergistic Inhibition of *Candida Albicans* by Cranberry Proanthocyanidins and Probiotics: Novel Strategies for Vulvovaginal Candidiasis Treatment. Pathogens.

[170] Yu C., Wang D., Yang Z., Wang T. (2022). Pharmacological Effects of Polyphenol Phytochemicals on the Intestinal Inflammation via Targeting TLR4/NF-κB Signaling Pathway. Int. J. Mol. Sci..

[171] Merenstein D., Pot B., Leyer G., Ouwehand A.C., Preidis G.A., Elkins C.A., Hill C., Lewis Z.T., Shane A.L., Zmora N. (2023). Emerging Issues in Probiotic Safety: 2023 Perspectives. Gut Microbes.

[172] Holkem A.T., Silva M.P.D., Favaro-Trindade C.S. (2023). Probiotics and Plant Extracts: A Promising Synergy and Delivery Systems. Crit. Rev. Food Sci. Nutr..

[173] Ahmad H.A., Qazi N.G., Jalal N., Khan M.I., Mishra N., Ashique S., Gowda B.H.J., Farid A., Garg A. (2024). Role of Flavonoids and Probiotics for Maintaining Healthy Gut Microbiota in Response to Chronic Metabolic Diseases. Role of Flavonoids in Chronic Metabolic Diseases.

[174] Guan C., Mei X., Sun S., Ding Y., Cai J. (2025). Dietary Quercetin Mitigates DON-Induced Intestinal Injury via Inhibiting MAPK/NF-κB-Mediated Pyroptosis and Tight Junction Disruption in Chicken. J. Agric. Food Chem..

[175] Lv Y., Peng J., Ma X., Liang Z., Salekdeh G.H., Ke Q., Shen W., Yan Z., Li H., Wang S. (2024). Network Analysis of Gut Microbial Communities Reveals Key Reason for Quercetin Protects against Colitis. Microorganisms.

[176] Maqoud F., Orlando A., Tricarico D., Antonacci M., Di Turi A., Giannelli G., Russo F. (2024). Anti-Inflammatory Effects of a Novel Acetonitrile–Water Extract of *Lens Culinaris* against LPS-Induced Damage in Caco-2 Cells. Int. J. Mol. Sci..

[177] Che S., Yuan J., Zhang L., Ruan Z., Sun X., Lu H. (2020). Puerarin Prevents Epithelial Tight Junction Dysfunction Induced by Ethanol in Caco-2 Cell Model. J. Funct. Foods.

[178] Xu B., Zhuang Y., Zhang Y., Liu S., Fan R., Jiang W. (2025). Apigenin Alleviates Intestinal Ischemia/Reperfusion Injury via Upregulating Nrf2-Mediated Tight Junction Integrity. Mol. Nutr. Food Res..

[179] Kiss A.K., Piwowarski J.P. (2019). Ellagitannins, Gallotannins and Their Metabolites- The Contribution to the Anti-Inflammatory Effect of Food Products and Medicinal Plants. Curr. Med. Chem..

[180] Oliveira S.D.S., Honório Da Silva J.V., Vieira R.D.S., Moreira L.F.S., Bandeira P.H.A., Ramos B.L., Silva M.A.A., Câmara N.O.S. (2025). SARM1: A Key Multifaceted Component in Immunoregulation, Inflammation and Neurodegeneration. Front. Immunol..

[181] Kim S.Y., Heo S., Kim S.H., Kwon M., Sung N.J., Ryu A.-R., Lee M.-Y., Park S.-A., Youn H.-S. (2020). Suppressive Effects of Dehydrocostus Lactone on the Toll-like Receptor Signaling Pathways. Int. Immunopharmacol..

[182] Rahman M., Rahaman S., Islam R., Rahman F., Mithi F.M., Alqahtani T., Almikhlafi M.A., Alghamdi S.Q., Alruwaili A.S., Hossain S. (2021). Role of Phenolic Compounds in Human Disease: Current Knowledge and Future Prospects. Molecules.

[183] Kataki C., Alexiou A., Jha S.K., Pandey R. (2025). Unveiling the Anti-Inflammatory and Immunomodulatory Effects of Secondary Metabolites: In Secondary Metabolites and Their Applications in Various Diseases.

[184] Ong G.H., Ori D., Kawasaki T., Kawai T. (2022). Inhibition of Lipopolysaccharide-induced Inflammatory Responses by 1′-acetoxychavicol Acetate. Genes Cells.

[185] Favari C., Rinaldi De Alvarenga J.F., Sánchez-Martínez L., Tosi N., Mignogna C., Cremonini E., Manach C., Bresciani L., Del Rio D., Mena P. (2024). Factors Driving the Inter-Individual Variability in the Metabolism and Bioavailability of (Poly)Phenolic Metabolites: A Systematic Review of Human Studies. Redox Biol..

[186] Hu J., Mesnage R., Tuohy K., Heiss C., Rodriguez-Mateos A. (2024). (Poly)Phenol-Related Gut Metabotypes and Human Health: An Update. Food Funct..

[187] Bongiovanni T., Yin M.O.L., Heaney L.M. (2021). The Athlete and Gut Microbiome: Short-Chain Fatty Acids as Potential Ergogenic Aids for Exercise and Training. Int. J. Sports Med..

[188] Tang Y., Fu A., Wang L., Ge Q. (2025). Microbiota-Dependent Metabolites—New Engine for T Cell Warriors. Gut Microbes.

[189] Zeng L., Qian Y., Cui X., Zhao J., Ning Z., Cha J., Wang K., Ge C., Jia J., Dou T. (2025). Immunomodulatory Role of Gut Microbial Metabolites: Mechanistic Insights and Therapeutic Frontiers. Front. Microbiol..

[190] Tomás-Barberán F.A., González-Sarrías A., García-Villalba R., Núñez-Sánchez M.A., Selma M.V., García-Conesa M.T., Espín J.C. (2017). Urolithins, the Rescue of “Old” Metabolites to Understand a “New” Concept: Metabotypes as a Nexus among Phenolic Metabolism, Microbiota Dysbiosis, and Host Health Status. Mol. Nutr. Food Res..

[191] Mandal S., Goswami R.K., Mavi A.K., Kumar S., Khangembam B.K., Rizvi M.A., Fakhri K.U., Borah D. (2026). Anti-Inflammatory Phytochemicals: Alleviating Inflammation and Immune Responses. Phytoceuticals in Food for Health and Wellness.

[192] De Souza E.L., De Albuquerque T.M.R., Dos Santos A.S., Massa N.M.L., De Brito Alves J.L. (2019). Potential Interactions among Phenolic Compounds and Probiotics for Mutual Boosting of Their Health-Promoting Properties and Food Functionalities—A Review. Crit. Rev. Food Sci. Nutr..

[193] Ghosh S., Basu S., Anbarasu A., Ramaiah S. (2025). A Comprehensive Review of Antimicrobial Agents Against Clinically Important Bacterial Pathogens: Prospects for Phytochemicals. Phytother. Res..

[194] Elafify M., Bakry A.M., Tian H., Huang J. (2025). Phytochemicals as Natural Antimicrobials: A Promising Strategy for Food Safety and Foodborne Pathogens Control. J. Food Saf..

[195] Makiej A., Bursztyn O., Nowak A., Guzik U., Smułek W., Kaczorek E. (2025). Plant Surfactants and Acidic pH Increase the Activity of Tobramycin and Kanamycin Against Escherichia Coli. Environ. Process..

[196] Coelho P., Oliveira J., Fernandes I., Araújo P., Pereira A.R., Gameiro P., Bessa L.J. (2021). Pyranoanthocyanins Interfering with the Quorum Sensing of *Pseudomonas Aeruginosa* and *Staphylococcus Aureus*. Int. J. Mol. Sci..

[197] Horn T., Bettray W., Noll U., Krauskopf F., Huang M.-R., Bolm C., Slusarenko A.J., Gruhlke M.C.H. (2020). The Sulfilimine Analogue of Allicin, S-Allyl-S-(S-Allyl)-N-Cyanosulfilimine, Is Antimicrobial and Reacts with Glutathione. Antioxidants.

[198] Gruhlke M.C.H., Antelmann H., Bernhardt J., Kloubert V., Rink L., Slusarenko A.J. (2019). The Human Allicin-Proteome: S-Thioallylation of Proteins by the Garlic Defence Substance Allicin and Its Biological Effects. Free Radic. Biol. Med..

[199] Jamel D.S., Malik S.T.A., Hassan A.K., Ayesh A.A. (2025). Biological Functions of Garlic (*Allium sativum* L.) and Its Active Compounds against Pathogens. Plant Prot..

[200] Leontiev R., Hohaus N., Jacob C., Gruhlke M.C.H., Slusarenko A.J. (2018). A Comparison of the Antibacterial and Antifungal Activities of Thiosulfinate Analogues of Allicin. Sci. Rep..

[201] Magryś A., Olender A., Tchórzewska D. (2021). Antibacterial Properties of *Allium Sativum* L. against the Most Emerging Multidrug-Resistant Bacteria and Its Synergy with Antibiotics. Arch. Microbiol..

[202] Murtiastutik D., Bintanjoyo L., Wibisono Y. (2025). Epigallocatechin Gallate and Its Antifungal Profiles. Tea in Health and Disease Prevention.

[203] Köksoy H., Ragbetli C. (2024). Evaluation of In Vitro Antimicrobial Activity of Epigallocatechin Gallate (EGCG) and Green Tea (*Camellia sinensis*) Oil on Various Pathogens. Genel Tıp Derg..

[204] Liang W., Fernandes A.P., Holmgren A., Li X., Zhong L. (2016). Bacterial Thioredoxin and Thioredoxin Reductase as Mediators for Epigallocatechin 3-gallate-induced Antimicrobial Action. FEBS J..

[205] Sidarningsih S., Yuliati Y., Saputra D., Ramadhani N.F., Rochmat S.A., Fauzia B., Aljunaid M.A., Qaid H.R., Ridwan R.D., Alaghbari S.G. (2025). Antimicrobial Effect of Stem Cells from Human Exfoliated Deciduous Teeth Metabolites Combined with Epigallocatechin-3-Gallate: As In Vitro Study. Res. J. Pharm. Technol..

[206] Wnuk E., Zwolak I. (2025). Preliminary Study on EGCG-Enhanced Vanadium Toxicity in Cells: Impact on Oxidative Stress. Molecules.

[207] Younes M., Aggett P., Aguilar F., Crebelli R., Dusemund B., Filipič M., Frutos M.J., Galtier P., Gott D., EFSA Panel on Food Additives and Nutrient Sources added to Food (ANS) (2018). Scientific Opinion on the Safety of Green Tea Catechins. EFSA J..

[208] Blaskovich M.A.T., Kavanagh A.M., Elliott A.G., Zhang B., Ramu S., Amado M., Lowe G.J., Hinton A.O., Pham D.M.T., Zuegg J. (2021). The Antimicrobial Potential of Cannabidiol. Commun. Biol..

[209] Turck D., Bohn T., Cámara M., Castenmiller J., De Henauw S., Jos Á., Maciuk A., Mangelsdorf I., McNulty B., EFSA Panel on Nutrition, Novel Foods and Food Allergens (NDA) (2026). Update of the Statement on Safety of Cannabidiol as a Novel Food. EFSA J..

[210] Garzón H.S., Loaiza-Oliva M., Martínez-Pabón M.C., Puerta-Suárez J., Téllez Corral M.A., Bueno-Silva B., Suárez D.R., Díaz-Báez D., Suárez L.J. (2024). Antibiofilm and Immune-Modulatory Activity of Cannabidiol and Cannabigerol in Oral Environments—In Vitro Study. Antibiotics.

[211] Aqawi M., Gallily R., Sionov R.V., Zaks B., Friedman M., Steinberg D. (2020). Cannabigerol Prevents Quorum Sensing and Biofilm Formation of *Vibrio Harveyi*. Front. Microbiol..

[212] Kulpa J., Lefever T.W., Trexler K.R., Henderson R.G., MacNair L., Toth M.L., Vanapalli S.A., Rahman M., Gupta S., Bonn-Miller M.O. (2023). Toxicity of Cannabigerol: Examination of Long-Term Toxicity and Lifespan in *Caenorhabditis elegans* and 14-Day Study in Sprague Dawley Rats. Cannabis Cannabinoid Res..

[213] Doyle A.A., Krämer T., Kavanagh K., Stephens J.C. (2019). Cinnamaldehydes: Synthesis, Antibacterial Evaluation, and the Effect of Molecular Structure on Antibacterial Activity. Results Chem..

[214] Ibrahim Al Ahadeb J. (2022). Impact of Cinnamomum Verum against Different *Escherichia Coli* Strains Isolated from Drinking Water Sources of Rural Areas in Riyadh, Saudi Arabia. J. King Saud. Univ.—Sci..

[215] Vijayan V., Mazumder A. (2018). In Vitro Inhibition of Food Borne Mutagens Induced Mutagenicity by Cinnamon (*Cinnamomum cassia*) Bark Extract. Drug Chem. Toxicol..

[216] Gholizadeh P., Rahimzadeh R., Mohammadi-Ghalehbin B., Arzanlou M., Soozangar N., Feizi H. (2026). Enhanced Antibiofilm and Gene-Suppressive Effects of Cinnamon Nanoemulsion against Multidrug-Resistant *Enterococcus Faecalis*. Mol. Biol. Rep..

[217] Wang Y., Zhang Y., Shi Y., Pan X., Lu Y., Cao P. (2018). Antibacterial Effects of Cinnamon (*Cinnamomum zeylanicum*) Bark Essential Oil on *Porphyromonas Gingivalis*. Microb. Pathog..

[218] Vasconcelos N.G., Croda J., Simionatto S. (2018). Antibacterial Mechanisms of Cinnamon and Its Constituents: A Review. Microb. Pathog..

[219] Honma M., Yamada M., Yasui M., Horibata K., Sugiyama K., Masumura K. (2021). In Vivo and in Vitro Mutagenicity of Perillaldehyde and Cinnamaldehyde. Genes Environ..

[220] Budiastuti, Lestari N.D., Effendi M.H., Arimbi, Plumeriastuti H. (2020). Cytotoxic Effect of Essential Oil from Cinnamon (*Cinnamomum Burmannii*) Bark on Rat Bone Marrow Mesenchymal Stem Cells: In Vitro Study. Syst. Rev. Pharm..

[221] Vaughn A.R., Haas K.N., Burney W., Andersen E., Clark A.K., Crawford R., Sivamani R.K. (2017). Potential Role of Curcumin Against Biofilm-Producing Organisms on the Skin: A Review. Phytother. Res..

[222] Shoaib S., Jahan R., Rehman A.S., Shadab M., Chauhan W., Alomary M.N., Ansari M.A., Islam N., Ansari M.A., Shoaib S., Islam N. (2024). Medicinal and Nutritional Importance of *Curcuma Longa* in Human Health. Medicinal Plants and their Bioactive Compounds in Human Health: Volume 1.

[223] Fatima M., Hussain S., Babar M., Shahzad M., Zafar M.S., Radhakrishnan N., Vasantha S., Pandurangan A.K. (2023). Curcumin: A Potential Therapeutic Agent for Human Health and Diseases—Current Status and Future Perspectives. Advances in Medical Diagnosis, Treatment, and Care.

[224] Pujari G., Nagalaxmi, Paripuranam T.D., Yadav B.K., Deeba F., Bhore P.G., Pooja, Upadhyay P.D. (2025). In Vitro Assessment of Antimicrobial and Antioxidant Properties of *Glycyrrhiza Glabra* Juice Powder. J. Exp. Zool. India.

[225] Talib AlSaady A., Al Mousawi H., Saleh R., Omran A., Ghasemian A. (2022). Chemical Analysis and Antibacterial Activity of *Glycyrrhiza Glabra* Roots. Egypt. J. Chem..

[226] Chen R.-Y., Shi J.-J., Liu Y.-J., Yu J., Li C.-Y., Tao F., Cao J.-F., Yang G.-J., Chen J. (2024). The State-of-the-Art Antibacterial Activities of Glycyrrhizin: A Comprehensive Review. Microorganisms.

[227] Kwon Y.-J., Son D.-H., Chung T.-H., Lee Y.-J. (2020). A Review of the Pharmacological Efficacy and Safety of Licorice Root from Corroborative Clinical Trial Findings. J. Med. Food.

[228] Nazari S., Rameshrad M., Hosseinzadeh H. (2017). Toxicological Effects of *Glycyrrhiza glabra* (Licorice): A Review. Phytother. Res..

[229] Semenescu I., Avram S., Similie D., Minda D., Diaconeasa Z., Muntean D., Lazar A.E., Gurgus D., Danciu C. (2024). Phytochemical, Antioxidant, Antimicrobial and Safety Profile of *Glycyrrhiza glabra* L. Extract Obtained from Romania. Plants.

[230] Khan U., Karmakar B.C., Basak P., Paul S., Gope A., Sarkar D., Mukhopadhyay A.K., Dutta S., Bhattacharya S. (2023). Glycyrrhizin, an Inhibitor of HMGB1 Induces Autolysosomal Degradation Function and Inhibits *Helicobacter Pylori* Infection. Mol. Med..

[231] Jawad M., Ijam A., Mohammed J., Mohammed F., Awad S., El-Rashedy A. (2025). Synergistic Anti-*Helicobacter Pylori* Effect of Licorice Extract and Metronidazole-2-Thiouracil Derivatives: In Vitro and in Silico Considerations. Trop. J. Pharm. Res..

[232] Lee H.-A., Kim J.-Y., Kim J., Nam B., Kim O. (2020). Anti-Helicobacter Pylori Activity of Acomplex Mixture of *Lactobacillus Paracasei* HP7 Including the Extract of Perilla Frutescens Var. Acuta and Glycyrrhiza Glabra. Lab. Anim. Res..

[233] Donkor M.N., Donkor A.-M., Mosobil R. (2023). Combination Therapy: Synergism among Three Plant Extracts against Selected Pathogens. BMC Res. Notes.

[234] Mensah Donkor A. (2016). In Vitro Bacteriostatic and Bactericidal Activities of *Senna Alata, Ricinus Communis* and *Lannea Barteri* Extracts Against Wound and Skin Disease Causing Bacteria. J. Anal. Pharm. Res..

[235] Nda-Umar U.I., Gbate M., Nda Umar A., Alfa Y.M., Mann A. (2017). Phytochemical and Acute Toxicity Studies of Methanolic Extracts of Selected Antimalarial Plants of Nupeland, North Central Nigeria. J. Med. Plants Res..

[236] Maggini V., Pesavento G., Maida I., Nostro A.L., Calonico C., Sassoli C., Perrin E., Fondi M., Mengoni A., Chiellini C. (2017). Exploring the Effect of the Composition of Three Different Oregano Essential Oils on the Growth of Multidrug-Resistant Cystic Fibrosis *Pseudomonas Aeruginosa* Strains. Nat. Prod. Commun..

[237] Falleh H. (2025). Demystifying the Power of Essential Oils: A Review of Their Antibacterial Properties and Potential as Natural Food Preservatives. EXCLI J..

[238] Qi Y., Zhao W., Wang T., Pei F., Yue M., Li F., Liu X., Wang X., Li H. (2020). Proteomic Analysis of the Antimicrobial Effects of Sublethal Concentrations of Thymol on *Salmonella Enterica* Serovar Typhimurium. Appl. Microbiol. Biotechnol..

[239] Ermenlieva N. (2025). New Perspectives In The Antimicrobial Potential Of Thyme And Oregano Essential Oils. Farmacia.

[240] Marchese A., Orhan I.E., Daglia M., Barbieri R., Di Lorenzo A., Nabavi S.F., Gortzi O., Izadi M., Nabavi S.M. (2016). Antibacterial and Antifungal Activities of Thymol: A Brief Review of the Literature. Food Chem..

[241] Bampidis V., Azimonti G., Bastos M.L., Christensen H., Durjava M., Dusemund B., Kouba M., López-Alonso M., López Puente S., EFSA Panel on Additives and Products or Substances used in Animal Feed (FEEDAP) (2024). Efficacy of a Feed Additive Consisting of Carvacrol (Nimicoat^®^) for Weaned Piglets (Techna France Nutrition). EFSA J..

[242] Sampaio L.A., Pina L.T.S., Serafini M.R., Tavares D.D.S., Guimarães A.G. (2021). Antitumor Effects of Carvacrol and Thymol: A Systematic Review. Front. Pharmacol..

[243] Bampidis V., Azimonti G., Bastos M.L., Christensen H., Kouba M., Kos Durjava M., López-Alonso M., López Puente S., Marcon F., EFSA Panel on Additives and Products or Substances used in Animal Feed (FEEDAP) (2019). Safety and Efficacy of an Essential Oil from *Origanum Vulgare* ssp. *Hirtum* (Link) Ietsw. for All Animal Species. EFSA J..

[244] Banc R., Rusu M.E., Filip L., Popa D.-S. (2023). The Impact of Ellagitannins and Their Metabolites through Gut Microbiome on the Gut Health and Brain Wellness within the Gut–Brain Axis. Foods.

[245] Yin Y., Martínez R., Zhang W., Estévez M. (2024). Crosstalk between Dietary Pomegranate and Gut Microbiota: Evidence of Health Benefits. Crit. Rev. Food Sci. Nutr..

[246] Elnawasany S., Soneji J.R., Nageswara-Rao M. (2018). Clinical Applications of Pomegranate. Breeding and Health Benefits of Fruit and Nut Crops.

[247] Vlachojannis C., Zimmermann B.F., Chrubasik-Hausmann S. (2015). Efficacy and Safety of Pomegranate Medicinal Products for Cancer. *Evid.-Based Complement*. Altern. Med..

[248] Zare H., Amiri Ardekani E., Tavakoli A., Bradley R., Tavakoli F., Pasalar M. (2024). Reporting of Adverse Effects of Pomegranate in Clinical Studies: A Systematic Review. J. Complement. Integr. Med..

[249] Banu T.N. (2019). Antibacterial Activity of Pomegranate (*Punica Granatum*) Fruit Peel Extracts Against Antibiotic Resistant Gram- Negative Pathogenic Bacteria. Biosci. Biotechnol. Res. Commun..

[250] Acharya D., Parida P., Mohapatra H.S., Mallik S., Mohanty J.N., Sahoo S.L. (2025). Phytochemical Analysis and Evaluation of Inhibitory Potential of *Ricinus Communis* Leaf Extract against Novel *Bacillus* Pathogens. Beni-Suef Univ. J. Basic Appl. Sci..

[251] Furquim Dos Santos Cardoso V., Amaral Roppa R.H., Antunes C., Silva Moraes A.N., Santi L., Konrath E.L. (2021). Efficacy of Medicinal Plant Extracts as Dental and Periodontal Antibiofilm Agents: A Systematic Review of Randomized Clinical Trials. J. Ethnopharmacol..

[252] Suurbaar J., Mosobil R., Donkor A.-M. (2017). Antibacterial and Antifungal Activities and Phytochemical Profile of Leaf Extract from Different Extractants of *Ricinus Communis* against Selected Pathogens. BMC Res. Notes.

[253] Franke H., Scholl R., Aigner A. (2019). Ricin and Ricinus Communis in Pharmacology and Toxicology-from Ancient Use and “Papyrus Ebers” to Modern Perspectives and “Poisonous Plant of the Year 2018”. Naunyn-Schmiedeberg’s Arch. Pharmacol..

[254] AL-Jborrey M.H., Altaie M.A.K., Al-Shahwany A.W. (2018). Study the Acute & Sub Acute Toxicity of *Ricinus Cummunis* Lnn. Ethanol Extract of Seed in Albino Mice. Int. J. Sci. Res. Manag..

[255] Roger K.K., Joachim A.E., Guy-Jocelin A.M., Sindou K., Yamousso T.A. (2026). Phytochemical Screening and Acute Toxicity of the Aqueous Extract of *Ricinus Communis* (*Euphorbiaceae*) Seeds in Male *Rattus Novrvegicus* (*Muridae*) Wistar Strain Rats. J. Pharmacogn. Phytochem..

[256] Biscotti F., Bortolotti M., Falà F., Di Maro A., Bolognesi A., Polito L. (2025). Ricin Toxicity to Intestinal Cells Leads to Multiple Cell Death Pathways Mediated by Oxidative Stress. Toxins.

[257] Makhammra J.M., Basheer-Salimia R., Hejaz H.A. (2024). Exploring the Potent Antioxidant and Antibacterial Properties of *Rosmarinus Officinalis* L. Leaf Extract: Health-Promoting Benefits of Rosemary Leaf Extract. Palest. Med. Pharm. J..

[258] Mahboub N., Cherfi I., Laouini S.E., Bouafia A., Benaissa A., Alia K., Alharthi F., Al-Essa K., Menaa F. (2025). GC/MS and LC Composition Analysis of Essential Oil and Extracts From Wild Rosemary: Evaluation of Their Antioxidant, Antimicrobial, and Anti-Inflammatory Activities. Biomed. Chromatogr..

[259] Myali A.A.H.A., Hassoon A.S., Hussain M.H., Rashed E.M. (2020). Reversed Phase Liquid Chromatographic-Ultra Violet Detection and Evaluation of Phenolic Antioxidants in Fresh Rosemary Leaves and Determination of Antibacterial Activity of Extract. AIP Conf. Proc..

[260] Milyuhina A.K., Zabodalova L.A., Kyzdarbek U., Romazyaeva I.R., Klyuchko N.Y. (2021). In Vitro Antibacterial and Antioxidant Activity of *Rosmarinus officinalis*. E3S Web Conf..

[261] Husein N., Laban N.A., Owais D.T. (2025). Exploring the Antimicrobial Potential of Rosmarinus Officinalis against Urinary Tract Infection Isolates in Amman, Jordan. Iran. J. Microbiol..

[262] Sahlabgi A., Lupuliasa D., Stanciu G., Lupșor S., Vlaia L.L., Rotariu R., Predescu N.C., Rădulescu C., Olteanu R.-L., Stănescu S.-G. (2025). The Development and Comparative Evaluation of Rosemary Hydroalcoholic Macerate-Based Dermatocosmetic Preparations: A Study on Antioxidant, Antimicrobial, and Anti-Inflammatory Properties. Gels.

[263] Bayram O.Y. (2025). Integration of Rosemary Oil (*Rosmarinus officinalis* L.) into Kefir: Phytochemical Profiling and Functional Impacts. Biocatal. Agric. Biotechnol..

[264] Villa R.E., Azimonti G., Bonos E., Christensen H., Durjava M., Dusemund B., Gehring R., Glandorf B., Kouba M., EFSA Panel on Additives and Products or Substances used in Animal Feed (FEEDAP) (2025). Safety and Efficacy of Feed Additives Consisting of Rosemary Tinctures Obtained from the Leaves of *Salvia Rosmarinus* Spenn. for Use in All Animal Species (FEFANA Asbl). EFSA J..

[265] Younes M., Aggett P., Aguilar F., Crebelli R., Dusemund B., Filipič M., Frutos M.J., Galtier P., Gott D., EFSA Panel on Food Additives and Nutrient Sources added to Food (EFSA ANS Panel) (2018). Refined Exposure Assessment of Extracts of Rosemary (E 392) from Its Use as Food Additive. EFSA J..

[266] Bampidis V., Azimonti G., Bastos M.L., Christensen H., Fašmon Durjava M., Kouba M., López-Alonso M., López Puente S., Marcon F., EFSA Panel on Additives, Products or Substances used in Animal Feed (FEEDAP) (2022). Safety and Efficacy of Two Solvent Extracts of Rosemary (*Rosmarinus officinalis* L.) When Used as Feed Additive for Cats and Dogs (Kemin Nutrisurance Europe SRL). EFSA J..

[267] Chillal N., Meti V.K., Swamy A.V., Kanakal M.M. (2025). Polyherbal Formulation and Evaluation of Topical Liquid Body Wash Containing Ashwagandha and *Senna Alata* Herbal Extracts for Anti-Acne and Anti -Microbial Activity. Res. J. Pharm. Technol..

[268] Rahim N.A., Ferdosh S., Zainuddin N.A.N.B., Sarker Z.I. (2023). Extraction Methodologies, Phytochemical Constituents, and Biological Activities of *Senna Alata* Linn: A Review. Nat. Prod. J..

[269] Yagi S., Cetiz M.V., Zengin G., Bakar K., Himidi A.A., Mohamed A., Skorić M., Glamočlija J., Gašić U. (2025). Novel Natural Candidates for Replacing Synthetic Additives in Nutraceutical and Pharmaceutical Areas: Two *Senna* Species (*S. alata* (L.) Roxb. and *S. occidentalis* (L.) Link). Food Sci. Nutr..

[270] Adesanya T., Salami W., Ajoseh S., Lawal-Sanni A., Akinyemi K. (2026). Activities of Two Medicinal Plant Extracts on Multi-Drug-Resistant (MDR) ESKAPE Pathogens from Different Environments in Lagos, Nigeria. Trop. J. Nat. Prod. Res..

[271] Roy S., Lyndem L.M. (2025). Exploring Metabolic Disruption and Redox Modulation by *Senna* Leaf Extracts Induces Mortality in the Zoonotic Parasite *Hymenolepis diminuta*. J. Parasitol. Res..

[272] Karki D., Pandey B., Jha P., Acharya A., Khanal D.P., Raut B., Panthi S. (2024). Senna Alata: Phytochemistry, Antioxidant, Thrombolytic, Anti-Inflammatory, Cytotoxicity, Antibacterial Activity, and GC-MS Analysis. Jordan J. Pharm. Sci..

[273] Roy S., Ukil B., Lyndem L.M. (2016). Acute and Sub-Acute Toxicity Studies on the Effect of *Senna alata* in Swiss Albino Mice. Cogent Biol..

[274] Uduma A.E., Abdulmajeed O., Chimezie A., Omomoni A.M., Doutimi O., Sylvester E.E. (2025). Development and Evaluation of a *Cassia Alata* L. Ethanolic Leaf Extract-Based Liquid Disinfectant with Broad-Spectrum Antimicrobial Activity. SSR J. Multidiscip..

[275] Ivanov M., Kostić M., Stojković D., Soković M. (2022). Rosmarinic Acid–Modes of Antimicrobial and Antibiofilm Activities of a Common Plant Polyphenol. S. Afr. J. Bot..

[276] Nallathambi R., Poulev A., Zuk J.B., Raskin I. (2020). Proanthocyanidin-Rich Grape Seed Extract Reduces Inflammation and Oxidative Stress and Restores Tight Junction Barrier Function in Caco-2 Colon Cells. Nutrients.

[277] Song Y., Sun M., Feng L., Liang X., Song X., Mu G., Tuo Y., Jiang S., Qian F. (2020). Antibiofilm Activity of *Lactobacillus Plantarum* 12 Exopolysaccharides against *Shigella Flexneri*. Appl. Environ. Microbiol..

[278] Sharif H.B., Shitu S., Shehu A., Abdurrauf I.R. (2022). Toxicity Profile of Aqueous Leaves Extract of *Vitis Vinifera* (Purple Grapes) on Wistar Albino Rats. Bayero J. Pure Appl. Sci..

[279] Juariah S., Abu Bakar F.I., Abu Bakar M.F., Kartini S., Dewi A.P., Surya A., Endrini S. (2024). Effectiveness and Mechanism of *Zingiber Officinale* Var. *Rubrum* (Red Ginger) Ethanol Extract as an Inhibitor of *Escherichia Coli* and *Staphylococcus Aureus*. Food Res..

[280] Kim J., Ha J., Kim S., Kim G., Shin H. (2025). Impact of Ginger on Gut Microbiota Composition and Function in a *Bacteroides* -Dominant Enterotype. J. Microbiol. Biotechnol..

[281] Lara A., Santos I.C.D., Soares A.A., Otutumi L.K., Jacomassi E., Lovato E.C.W., Gazim Z.C., Rahal I.L., Oliva L.R., Gonçalves J.E. (2021). Composition and Antimicrobial Activity of Ginger (*Zingiber Officinale* Roscoe). Aust. J. Crop Sci..

[282] Moulick S., Bera R., Roy D.N. (2025). Bactericidal Action of Ginger (*Zingiber Officinale* Roscoe) Extract against *Escherichia Coli* through Synergistic Modulation of the AcrAB-TolC Efflux Pump and Inhibition of Peptidoglycan Synthesis: In Vitro and in Silico Approaches. Microb. Pathog..

[283] Prakasita V.C., Asmara W., Widyarini S., Wahyuni A.E.T.H. (2019). Combinations of Herbs and Probiotics as an Alternative Growth Promoter: An in Vitro Study. Veter World.

[284] Shen L., Ji H.-F. (2025). Regulation of Gut Microbiota by Ginger, Its Derived Polysaccharides, Essential Oils, Gingerols, and Shogaols and Related Health Outcomes. Food Chem..

[285] Bampidis V., Azimonti G., Bastos M.L., Christensen H., Kos Durjava M., Kouba M., López-Alonso M., López Puente S., Marcon F., EFSA Panel on Additives and Products or Substances used in Animal Feed (FEEDAP) (2020). Safety and Efficacy of Essential Oil, Oleoresin and Tincture from *Zingiber Officinale* Roscoe When Used as Sensory Additives in Feed for All Animal Species. EFSA J..

[286] Olajuyigbe A.A., Olajuyigbe O.O., Coopoosamy R.M. (2020). Interaction of *Ziziphus Mucronata Subsp. Mucronata* Methanol Extract and First-Line Antibiotics Is Synergistic In Vitro through Production of Reactive Oxygen Species. J. Trop. Med..

[287] Silva S., Costa E.M., Machado M., Morais R.M., Calhau C., Pintado M. (2023). Selective Activity of an Anthocyanin-Rich, Purified Blueberry Extract upon Pathogenic and Probiotic Bacteria. Foods.

[288] Enany M.E., Hamouda A.M., Khashaba R.M. (2024). Synergistic Effect of Some Plant Extracts with Selected Antibiotics Against Enteric Pathogens of Turkey Poults. Adv. Anim. Veter-Sci..

[289] Nechchadi H., Nadir Y., Benhssaine K., Alem C., Sellam K., Boulbaroud S., Berrougui H., Ramchoun M. (2024). Hypolipidemic Activity of Phytochemical Combinations: A Mechanistic Review of Preclinical and Clinical Studies. Food Chem..

[290] Yanadaiah P., Babu M.R., Dureja H., Kumbhar P., Disouza J., Gupta G., Dua K., Singh S.K., Dua K. (2024). An Update on Clinical Trials on Synbiotics. Synbiotics in Human Health: Biology to Drug Delivery.

[291] Lavermicocca P., Dekker M., Russo F., Valerio F., Di Venere D., Sisto A. (2016). *Lactobacillus Paracasei*-Enriched Vegetables Containing Health Promoting Molecules. Probiotics, Prebiotics, and Synbiotics.

[292] Singh A., Kaur P., Kumar M., Shafi S., Upadhyay P.K., Tiwari A., Tiwari V., Rangra N.K., Thirunavukkarasu V., Kumari S. (2025). The Role of Phytochemicals in Modulating the Gut Microbiota: Implications for Health and Disease. Med. Microecol..

[293] Nikolic I., Aleksic Sabo V., Gavric D., Knezevic P. (2024). Anti-*Staphylococcus Aureus* Activity of Volatile Phytochemicals and Their Combinations with Conventional Antibiotics Against Methicillin-Susceptible *S. Aureus* (MSSA) and Methicillin-Resistant *S. Aureus* (MRSA) Strains. Antibiotics.

[294] Itrat N., Israr B., Shabbir J., Ameen F., Ali A. (2026). Phytochemicals in Fermented Foods: Health-Promoting Transformations and Probiotic Potential. Phytoceuticals in Food for Health and Wellness.

[295] Mehrotra N., Sharma S., Tripathi P. (2026). Alteration of Gut Microbiota and Their Metabolites by Dietary Phytochemicals. Phytochemicals and Gut Health.

[296] Singh V., Muthuramalingam K., Kim Y.M., Park S., Kim S.H., Lee J., Hyun C., Unno T., Cho M. (2021). Synbiotic Supplementation with Prebiotic Schizophyllum Commune Derived β-(1,3/1,6)-Glucan and Probiotic Concoction Benefits Gut Microbiota and Its Associated Metabolic Activities. Appl. Biol. Chem..

[297] Alsufyani M., Asiri A., Asiri Y., Alsughayyir I., Alzahrani R., Al Essa T., Lajhar A., Alshhrani M., Rabeh M. (2025). Correlation between Probiotic and Prebiotic: A Systematic Review. Egypt. J. Chem..

[298] Kaur K. (2025). Application and Challenges of Using Probiotic *Lactobacillus* and *Bifidobacterium* to Enhance Overall Health and Manage Diseases. Diseases.

[299] Zhang M., Meng G., Li H., Wu Z., Chen B., Zeng X., Pan D., Du Q. (2026). Encapsulation of Probiotics and Co-Encapsulation of Probiotic-Hydrophobic Bioactives Based on Emulsion Systems. Food Chem..

[300] Oliveira T.S.D.C., Gusmão J.V.F., Rigolon T.C.B., Wischral D., Campelo P.H., Martins E., Stringheta P.C. (2025). Bioactive Compounds and the Performance of Proteins as Wall Materials for Their Encapsulation. Micro.

[301] Nikitina E., Khrundin D. (2026). The Probiotic Bacteria and Their Encapsulated Forms as Food Components: Survival, Effects and Quality. AIMS Microbiol..

[302] Ahamed M.J.N., Ibrahim F.B., Srinivasan H. (2021). Synergistic Interactions of Antimicrobials to Counteract the Drug-Resistant Microorganisms. Biointerface Res. Appl. Chem..

[303] Gupta C., Kitdamrongtham W., Bakar M.F.A. (2023). Phyto-Nanoconjugates in Combating Multidrug Resistance in Medical Research. Antimicrobials in Pharmaceutical and Medicinal Research.

[304] Fan K., Hua X., Wang S., Efferth T., Tan S., Wang Z. (2025). A Promising Fusion: Traditional Chinese Medicine and Probiotics in the Quest to Overcome Osteoporosis. FASEB J..

[305] Liu Y., Liu C., Kou X., Wang Y., Yu Y., Zhen N., Jiang J., Zhaxi P., Xue Z. (2022). Synergistic Hypolipidemic Effects and Mechanisms of Phytochemicals: A Review. Foods.

[306] Singh B., Mal G., Sharma D., Sharma R., Antony C.P., Kalra R.S. (2020). Gastrointestinal Biotransformation of Phytochemicals: Towards Futuristic Dietary Therapeutics and Functional Foods. Trends Food Sci. Technol..

[307] Ren H., Vahjen W., Dadi T., Saliu E.-M., Boroojeni F.G., Zentek J. (2019). Synergistic Effects of Probiotics and Phytobiotics on the Intestinal Microbiota in Young Broiler Chicken. Microorganisms.

[308] Shabbir J., Israr B., Itrat N., Elfalleh W., Majeed M.R., Sarkar T. (2025). Neuroprotective and Cognitive Benefits of Mushrooms: A Biochemical Perspective. Mushroom Bioactives: Bridging Food, Biotechnology, and Nanotechnology for Health and Innovation.

[309] De Silva H.B., Dai Y., Homer-Vanniasinkam S., Edirisinghe M. (2026). Antimicrobial Effect of Spices and Their Phytochemicals: A Novel Approach to Overcoming Antibiotic Resistance. MedComm.

[310] Parihar H., Singh U., Pathak R., Chaturvedi P., Dayal R., Tirumalai P.S. (2025). Investigating the Individual and Combined Effect of Essential Oils and Probiotics against Staphylococcus Aureus. J. Food Qual. Hazards Control.

[311] Evdokimova S.A., Nokhaeva V.S., Karetkin B.A., Guseva E.V., Khabibulina N.V., Kornienko M.A., Grosheva V.D., Menshutina N.V., Shakir I.V., Panfilov V.I. (2021). A Study on the Synbiotic Composition of *Bifidobacterium Bifidum* and Fructans from *Arctium Lappa* Roots and *Helianthus Tuberosus* Tubers against *Staphylococcus Aureus*. Microorganisms.

[312] Farrag H.A., Abdallah N., Shehata M.M.K., Awad E.M. (2019). Natural Outer Membrane Permeabilizers Boost Antibiotic Action against Irradiated Resistant Bacteria. J. Biomed. Sci..

[313] El Atki Y., Aouam I., Taroq A., El Kamari F., Timinouni M., Lyoussi B., Abdellaoui A. (2020). Antibacterial Effect of Combination of Cinnamon Essential Oil and Thymol, Carvacrol, Eugenol, or Geraniol. J. Rep. Pharma Sci..

[314] Whitmore M., Tobin I., Burkardt A., Zhang G. (2024). Nutritional Modulation of Host Defense Peptide Synthesis: A Novel Host-Directed Antimicrobial Therapeutic Strategy?. Adv. Nutr..

[315] Wang J., Ma X., Li J., Shi L., Liu L., Hou X., Jiang S., Li P., Lv J., Han L. (2023). The Synergistic Antimicrobial Effect and Mechanism of Nisin and Oxacillin against Methicillin-Resistant *Staphylococcus Aureus*. Int. J. Mol. Sci..

[316] Sharma G., Dang S., K A., Kalia M., Gabrani R. (2020). Synergistic Antibacterial and Anti-Biofilm Activity of Nisin like Bacteriocin with Curcumin and Cinnamaldehyde against ESBL and MBL Producing Clinical Strains. Biofouling.

[317] Zhao S., Han J., Bie X., Lu Z., Zhang C., Lv F. (2016). Purification and Characterization of Plantaricin JLA-9: A Novel Bacteriocin against *Bacillus* Spp. Produced by *Lactobacillus Plantarum* JLA-9 from Suan-Tsai, a Traditional Chinese Fermented Cabbage. J. Agric. Food Chem..

[318] Bernal-Castro C., Espinosa-Poveda E., Gutiérrez-Cortés C., Díaz-Moreno C. (2024). Vegetable Substrates as an Alternative for the Inclusion of Lactic Acid Bacteria with Probiotic Potential in Food Matrices. J. Food Sci. Technol..

[319] Kurian S.J., Baral T., Sekhar M.S., Rao M. (2022). Role of Probiotics and Prebiotics in Digestion, Metabolism, and Immunity. Nutrition and Functional Foods in Boosting Digestion, Metabolism and Immune Health.

[320] Jiang F., Lu Y., Dong Y., Chi Y., Lv Y., He Q. (2025). Dual-Directional Regulation of Tea Polyphenols on *Probiotic Lactobacillus Plantarum* and Pathogenic *Staphylococcus Aureus*, and Its Effect on Quality of Dry-Fermented Sausage. Int. Food Res. J..

[321] Peng M., Patel P., Nagarajan V., Bernhardt C., Carrion M., Biswas D. (2019). Feasible Options to Control Colonization of Enteric Pathogens With Designed Synbiotics. Dietary Interventions in Gastrointestinal Diseases.

[322] Anand S., Mandal S., Singh K.S., Patil P., Tomar S.K. (2018). Synbiotic Combination of *Lactobacillus Rhamnosus* NCDC 298 and Short Chain Fructooligosaccharides Prevents Enterotoxigenic *Escherichia Coli* Infection. LWT.

[323] Khursheed H., Qasim R. (2024). Synergistic Antibioiflm Activity Of Probiotic *Lactobacillus Acidophilus* And *Punica Granatum* L., Against *Pseudomonas Aeruginosa* Biofilm. J. Ayub Med. Coll. Abbottabad.

[324] Jia M., Luo J., Bao Y., Zhang X., Chai Y., Li F., Jiang S., Xie Q. (2025). Synergistic Effects of Compound Plant Extracts and *Lactobacillus Plantarum* on Osteogenesis: Prebiotic Potential and Mechanistic Insights from Metabolomics and Molecular Docking. J. Sci. Food Agric..

[325] Ma M., Liu Y., Chen Y., Zhang S., Yuan Y. (2025). Co-Encapsulation: An Effective Strategy to Enhance the Synergistic Effects of Probiotics and Polyphenols. Trends Food Sci. Technol..

[326] Barzegari A., Kheyrolahzadeh K., Hosseiniyan Khatibi S.M., Sharifi S., Memar M.Y., Zununi Vahed S. (2020). The Battle of Probiotics and Their Derivatives Against Biofilms. Infect. Drug Resist..

[327] Abu El-Wafa W.M., Ahmed R.H., Ramadan M.A.-H. (2020). Synergistic Effects of Pomegranate and Rosemary Extracts in Combination with Antibiotics against Antibiotic Resistance and Biofilm Formation of *Pseudomonas Aeruginosa*. Braz. J. Microbiol..

[328] Song F., Liu J., Zhao W., Huang H., Hu D., Chen H., Zhang H., Chen W., Gu Z. (2020). Synergistic Effect of Eugenol and Probiotic *Lactobacillus Plantarum* Zs2058 against *Salmonella* Infection in C57bl/6 Mice. Nutrients.

[329] Zapletal K., Machnik G., Okopień B. (2022). Polyphenols of Antibacterial Potential—May They Help in Resolving Some Present Hurdles in Medicine?. Folia Biol..

[330] Liu J., Li W., Zhu X., Zhao H., Lu Y., Zhang C., Lu Z. (2019). Surfactin Effectively Inhibits *Staphylococcus Aureus* Adhesion and Biofilm Formation on Surfaces. Appl. Microbiol. Biotechnol..

[331] Englerová K., Bedlovičová Z., Nemcová R., Király J., Maďar M., Hajdučková V., Styková E., Mucha R., Reiffová K. (2021). *Bacillus Amyloliquefaciens*—Derived Lipopeptide Biosurfactants Inhibit Biofilm Formation and Expression of Biofilm-Related Genes of *Staphylococcus Aureus*. Antibiotics.

[332] Ren Y., Pei F., Cao X., Zhang W., Du R., Ge J., Ping W. (2023). Purification of Exopolysaccharides from *Lactobacillus Rhamnosus* and Changes in Their Characteristics by Regulating Quorum Sensing Genes via Polyphenols. Int. J. Biol. Macromol..

[333] Ye Y., Ze L., Duan M., Tan Z., Wang Y., Zhang H., Shang P. (2025). Protective Effects of Plant Polysaccharides on Intestinal Health via Targeted Regulation of Gut Microbiota. J. Sci. Food Agric..

[334] D’Arrigo M., Muscarà C., Molonia M.S., Cimino F., Gervasi T. (2024). Synbiotic Effect of Quercetin and Probiotic *Lactobacillus* SP. Protects Intestinal Barrier from *E. Coli*-Induced Challenge in Caco-2 Cells. J. Funct. Foods.

[335] Derebasi B.N., Davran Bulut S., Aksoy Erden B., Sadeghian N., Taslimi P., Celebioglu H.U. (2024). Effects of P-Coumaric Acid on Probiotic Properties of *Lactobacillus Acidophilus* LA-5 and *Lacticaseibacillus Rhamnosus* GG. Arch. Microbiol..

[336] Abreu A.C., Saavedra M.J., Simões L.C., Simões M. (2016). Combinatorial Approaches with Selected Phytochemicals to Increase Antibiotic Efficacy against *Staphylococcus Aureus* Biofilms. Biofouling.

[337] Krūmiņa A., Zeltiņa I., Simsone P., Eulitz E., Reinis A., Vīksna L. (2026). Mechanisms of *Pseudomonas Aeruginosa* Resilience Against Antibiotic Treatment and Outlooks of Emerging Treatment Strategies. Medicina.

[338] Prakash V., Krishnan A.S., Ramesh R., Bose C., Pillai G.G., Nair B.G., Pal S. (2021). Synergistic Effects of *Limosilactobacillus Fermentum* ASBT-2 with Oxyresveratrol Isolated from Coconut Shell Waste. Foods.

[339] Lal A.F., Singh S., Franco F.C., Bhatia S. (2021). Potential of Polyphenols in Curbing Quorum Sensing and Biofilm Formation in Gram-Negative Pathogens. Asian Pac. J. Trop. Biomed..

[340] John N., Ramesh S. (2020). Anti-Quorum Sensing Properties of Medicinal Plants—A Review.

[341] Elkhalifa M.E., Ashraf M., Ahmed A., Usman A., Hamdoon A.A., Elawad M.A., Almalki M.G., Mosa O.F., Niyazov L.N., Ayaz M. (2024). Polyphenols and Their Nanoformulations as Potential Antibiofilm Agents Against Multidrug-Resistant Pathogens. Future Microbiol..

[342] Dai J., Zhang C., Bai M., Aziz T., Alharbi N.K., Alshehri F., Shami A., Alhodieb F.S., Alsanie S.A., Alblaji M. (2026). Molecular Insights into Gallic Acid as a Quorum Sensing Inhibitor Targeting the LuxS/AI-2 System in Escherichia Coli O157: H7 and Its Antibiofilm Applications. Int. J. Food Microbiol..

[343] Nazareth M.S., Shreelakshmi S.V., Shetty N.P. (2021). Identification and Characterization of Polyphenols from *Carissa Spinarum* Fruit and Evaluation of Their Antioxidant and Anti-Quorum Sensing Activity. Curr. Microbiol..

[344] Pathak S., Banerjee A., Duttaroy A.K. (2024). Microbiota and Dietary Mediators in Colon Cancer Prevention and Treatment.

[345] Wan M.L.Y., Ling K.H., El-Nezami H., Wang M.F. (2019). Influence of Functional Food Components on Gut Health. Crit. Rev. Food Sci. Nutr..

[346] Zang J., Xiao L., Shi Y., Kou Y., Ma K., Zhang C., Rui X., Lin T., Li W. (2026). Advances in Dietary Modulation of the Intestinal Barrier: Mechanistic, Structural, and Functional Insights. Compr. Rev. Food Sci. Food Safe.

[347] König A., Sadova N., Dornmayr M., Schwarzinger B., Neuhauser C., Stadlbauer V., Wallner M., Woischitzschläger J., Müller A., Tona R. (2023). Combined Acid Hydrolysis and Fermentation Improves Bioactivity of Citrus Flavonoids in Vitro and in Vivo. Commun. Biol..

[348] Neginah V., Athiappan M. (2023). Bio-Transforming *Syzygium Cumini* Kernel into Microbial Metabolites: Towards Enhancing Pharmacological Characterization. Res. J. Biotechnol..

[349] Alves-Santos A.M., Sugizaki C.S.A., Lima G.C., Naves M.M.V. (2020). Prebiotic Effect of Dietary Polyphenols: A Systematic Review. J. Funct. Foods.

[350] Zou Y., Shi Y., Liao S., Li E., Yang Q., Chen R., Li Q. (2024). The Interaction between Mulberry Leaf Polyphenols and Polysaccharides during Digestion and Colon Fermentation. LWT.

[351] Zhou X., Qin Z., Chen Y., Wang Y., Du B., Lin D., Meng L. (2026). Effect of Bound Polyphenol Removal on in Vitro Digestion and Fermentation of *Rosa Roxburghii* Tratt. Dietary Fiber. J. Agric. Food Res..

[352] Rakhra G., Malhotra R., Prasad P., Sahu J.K., Rakhra G., Khan T.U., Khan M.U., Rastogi S. (2026). Synergistic Effects of Polyphenols and Gut Microbiota–Derived Metabolites on Inflammation and Metabolic Syndrome: A Review. Mol. Nutr. Food Res..

[353] Umu Ö.C.O., Bäuerl C., Oostindjer M., Pope P.B., Hernández P.E., Pérez-Martínez G., Diep D.B. (2016). The Potential of Class II Bacteriocins to Modify Gut Microbiota to Improve Host Health. PLoS ONE.

[354] Wang Y., Wang C., Shi J., Zhang Y. (2024). Effects of Derivatization and Probiotic Transformation on the Antioxidative Activity of Fruit Polyphenols. Food Chem. X.

[355] Balasubramaniam V.G., Jaiswal S., Roy L.M., Dhassiah M.P., Antony U. (2025). In Vitro Bi-Directional Metabolism of Lactic Acid Bacteria and Finger Millet Polyphenols. Food Biotechnol..

[356] Liu Y., Hui X., Ibrahim S.A., Huang W. (2018). Increasing Antiradical Activity of Polyphenols from Lotus Seed Epicarp by Probiotic Bacteria Bioconversion. Molecules.

[357] Xu R., Lee J., Zhang S., Chen L., Zhu J. (2022). Structure Similarity and Molecular Networking Analysis for the Discovery of Polyphenol Biotransformation Products of Gut Microbes. Anal. Chim. Acta.

[358] Yu X., Liu Z., Liu Y., Li X., Wang B., Yin J. (2025). Antimicrobial Roles of Probiotics: Molecular Mechanisms and Application Prospects. Trends Food Sci. Technol..

[359] Fadare O.S., Singh V., Enabulele O.I., Shittu O.H., Pradhan D. (2022). In Vitro Evaluation of the Synbiotic Effect of Probiotic *Lactobacillus* Strains and Garlic Extract against *Salmonella* Species. LWT.

[360] Fadare O.S., Enabulele O.I., Singh V., Pradhan D. (2025). GC-MS Based Metabolomic Profiling of Synbiotic Action of *Probiotic Lactobacilli* with Aqueous Garlic Extract against *Salmonella* spp. Microbe.

[361] Bauza-Kaszewska J., Żary-Sikorska E., Gugolek A., Ligocka A., Kosmala M., Karlińska E., Fotschki B., Juśkiewicz J. (2021). Synergistic Antimicrobial Effect of Raspberry (*Rubus idaeus* L., Rosaceae) Preparations and Probiotic Bacteria on Enteric Pathogens. Pol. J. Food Nutr. Sci..

[362] Elebeedy D., Ghanem A., El-Sayed M., Fayad E., Abu Ali O.A., Alyamani A., Sayed Abdelgeliel A. (2022). Synergistic Antimicrobial Effect of *Lactiplantibacillus Plantarum and Lawsonia Inermis* Against *Staphylococcus Aureus*. Infect. Drug Resist..

[363] Brennan-Krohn T., Kirby J.E. (2019). Antimicrobial Synergy Testing by the Inkjet Printer-Assisted Automated Checkerboard Array and the Manual Time-Kill Method. J. Vis. Exp..

[364] Kouhounde S., Adéoti K., Mounir M., Giusti A., Refinetti P., Otu A., Effa E., Ebenso B., Adetimirin V.O., Barceló J.M. (2022). Applications of Probiotic-Based Multi-Components to Human, Animal and Ecosystem Health: Concepts, Methodologies, and Action Mechanisms. Microorganisms.

[365] Rodriguero G.S., De Souza Aquino J., Gomes De Oliveira L.I., Silva F.D., Santos L.C., Céspedes-Acuña C.L., Magnani M., Pimentel T.C. (2026). Conceptual and Methodological Approaches Applied to Assessing Plant Food Byproducts as Prebiotics: A Critical Review of Evidence and Gaps. Crit. Rev. Food Sci. Nutr..

[366] Séguéla A., Della-Negra O., Gautier R., Hamelin J., Milferstedt K., Servien R., Teste M.-A., Canlet C. (2025). Integrated Co-Extraction Protocol for Transcriptomic and ^1^H NMR Metabolomic Analysis of Multi-Species Biofilms. Bio-Protocol.

[367] Aubry R., Buyck J., Prouvensier L., Decousser J.-W., Nordmann P., Wicha S.G., Marchand S., Grégoire N. (2023). An Improved PKPD Modeling Approach to Characterize the Pharmacodynamic Interaction over Time between Ceftazidime/Avibactam and Colistin from in Vitro Time-Kill Experiments against Multidrug-Resistant *Klebsiella pneumoniae* Isolates. Antimicrob. Agents Chemother..

[368] Tängdén T., Karvanen M., Friberg L.E., Odenholt I., Cars O. (2017). Assessment of Early Combination Effects of Colistin and Meropenem against *Pseudomonas aeruginosa* and *Acinetobacter baumannii* in Dynamic Time-Kill Experiments. Infect. Dis..

[369] Bishop J., McCue P.M., Dascanio J., McCue P. (2021). Antimicrobiotic Sensitivity Testing. Equine Reproductive Procedures.

[370] Allameh A., Ani F., Shams-Ghahfarokhi M., Razzaghi-Abyaneh M. (2019). In Vitro Anti-Mycotoxigenic and Anti-Aflatoxigenic Properties of Probiotic Bacteria; *Lactobacillus Plantarum* and *L. paracasei*. J. Sci..

[371] Kragh K.N., Alhede M., Kvich L., Bjarnsholt T. (2019). Into the Well—A Close Look at the Complex Structures of a Microtiter Biofilm and the Crystal Violet Assay. Biofilm.

[372] Fonseca S., Robidoux J., Cayer M.-P., Matte J., Charette S.J., Brouard D. (2025). Dynamic Monitoring of *Staphylococcus Epidermidis* Biofilm Formation Using a Microfluidic Approach. Microbe.

[373] Monteiro D.R., Roseno A.C.B., Ribeiro N.P., Hosida T.Y., Pessan J.P. (2025). Quantification of Microbial Biofilm Biomass. Microbial Biofilm Dynamics.

[374] Sampaio C., Morais L.A.D., Guisso L.P., Ferraresso L.F.D.O.T., Monteiro D.R., Pessan J.P., Delbem A.C.B., Hosida T.Y. (2025). Analytical Tools for Assessing Metabolic Activity in Biofilms. Microbial Biofilm Dynamics.

[375] Solokhina A., Bonkat G., Braissant O., Ennifar E. (2019). Measuring the Metabolic Activity of Mature Mycobacterial Biofilms Using Isothermal Microcalorimetry. Microcalorimetry of Biological Molecules.

[376] Li W., Wang J.J., Qian H., Tan L., Zhang Z., Liu H., Pan Y., Zhao Y. (2020). Insights Into the Role of Extracellular DNA and Extracellular Proteins in Biofilm Formation of Vibrio Parahaemolyticus. Front. Microbiol..

[377] Waheed H., Mehmood C.T., Yang Y., Tan W., Fu S., Xiao Y. (2022). Dynamics of Biofilms on Different Polymeric Membranes—A Comparative Study Using Five Physiologically and Genetically Distinct Bacteria. J. Membr. Sci..

[378] Bravo E., Arce M., Ribeiro-Vidal H., Herrera D., Sanz M. (2024). The Impact of Candida Albicans in the Development, Kinetics, Structure, and Cell Viability of Biofilms on Implant Surfaces—An In Vitro Study with a Validated Multispecies Biofilm Model. Int. J. Mol. Sci..

[379] Wełna J., Napiórkowska-Mastalerz M., Cyrankiewicz M., Bogiel T., Kwiecińska-Piróg J. (2025). Characteristic of Virulence and Parameters of Mixed Biofilm Formed by Carbapenem-Resistant *Pseudomonas Aeruginosa* and *Proteus Mirabilis* Strains Isolated from Infected Chronic Wounds. Pathogens.

[380] Sathiah S., Pan I. (2024). Growth Analysis of Lactococcus When Subjected to Low (25 °C) and High (45 °C) Temperature by Measuring the Optical Density for 2 Days to Study Antimicrobial Activity.

[381] McBirney S.E., Trinh K., Wong-Beringer A., Armani A.M. (2016). Wavelength-Normalized Spectroscopic Analysis of Staphylococcus Aureus and Pseudomonas Aeruginosa Growth Rates. Biomed. Opt. Express.

[382] Arroyo-Moreno S., Saiz-Gonzalo G., McSweeney S., Bleiel S.B. (2025). Probiotic Viability Reconsidered: Integrating VBNC Resuscitation and Culture-Independent Methods for Accurate Probiotic Enumeration. Microorganisms.

[383] Rizzi F., Juan B., Espadaler-Mazo J., Capellas M., Huedo P. (2024). *Lactiplantibacillus Plantarum* KABP051: Stability in Fruit Juices and Production of Bioactive Compounds During Their Fermentation. Foods.

[384] Farczadi L., Barcutean L., Maier S., Balasa R., Imre S. (2025). Development and Validation of a New LC-MS/MS Method for the Assay of Plasmatic Peripheral Short- and Medium-Chain Fatty Acids for Metabolomics Applications. Metabolites.

[385] Lotti C., Rubert J., Fava F., Tuohy K., Mattivi F., Vrhovsek U. (2017). Development of a Fast and Cost-Effective Gas Chromatography–Mass Spectrometry Method for the Quantification of Short-Chain and Medium-Chain Fatty Acids in Human Biofluids. Anal. Bioanal. Chem..

[386] Frolova N., Orlova A., Popova V., Bilova T., Frolov A. (2025). Gas Chromatography–Mass Spectrometry (GC-MS) in the Plant Metabolomics Toolbox: Sample Preparation and Instrumental Analysis. Biomolecules.

[387] Sengupta A., Weljie A.M. (2019). NMR Spectroscopy–Based Metabolic Profiling of Biospecimens. Curr. Protoc. Protein Sci..

[388] Shagun S., Lingwan M., Masakapalli S.K. (2024). Mass Spectrometry and Nuclear Magnetic Resonance Spectroscopy Profiles of Red and Pink Rhododendron Flower Petals Establish Them as Rich Sources of Bioactive Secondary Metabolites. Sep. Sci. Plus.

[389] Barros Ferreira L., Ashander L.M., Ma Y., Appukuttan B., Williams K.A., Best G., Smith J.R. (2024). Effects of Tumor Necrosis Factor-α and Interleukin-1β on Human Retinal Endothelial Cells. Cytokine.

[390] Demaurex N., Nunes P. (2016). The Role of STIM and ORAI Proteins in Phagocytic Immune Cells. Am. J. Physiol.-Cell Physiol..

[391] Karrasch T., Höpfinger A., Schäffler A., Schmid A. (2021). The Adipokine C1q/TNF-Related Protein-3 (CTRP-3) Inhibits Toll-like Receptor (TLR)-Induced Expression of Cathelicidin Antimicrobial Peptide (CAMP) in Adipocytes. Cytokine.

[392] Lee E.Y., Lee M.W., Wong G.C.L. (2019). Modulation of Toll-like Receptor Signaling by Antimicrobial Peptides. Semin. Cell Dev. Biol..

[393] Nerezenko A.M., Virolainen P.A., Tupitsyna S.A., Chekunova E.M. (2026). Development and Validation of the PipeSeq Program for RNA-Seq Data Analysis in the *Chlamydomonas Reinhardtii* as a Model. Vestn. VOGiS.

[394] Sampathkumar N.K., Sundaram V.K., Danthi P.S., Barakat R., Solomon S., Mondal M., Carre I., El Jalkh T., Padilla-Ferrer A., Grenier J. (2022). RNA-Seq Is Not Required to Determine Stable Reference Genes for qPCR Normalization. PLoS Comput. Biol..

[395] Brown A.C., Parish T., Kumar A. (2021). Whole-Genome Sequencing of *Mycobacterium Tuberculosis* Directly from Sputum Samples. Mycobacteria Protocols.

[396] Allard M.W., Bell R., Ferreira C.M., Gonzalez-Escalona N., Hoffmann M., Muruvanda T., Ottesen A., Ramachandran P., Reed E., Sharma S. (2018). Genomics of Foodborne Pathogens for Microbial Food Safety. Curr. Opin. Biotechnol..

[397] Herrera-Rocha K.M., Manjarrez-Juanes M.M., Larrosa M., Barrios-Payán J.A., Rocha-Guzmán N.E., Macías-Salas A., Gallegos-Infante J.A., Álvarez S.A., González-Laredo R.F., Moreno-Jiménez M.R. (2023). The Synergistic Effect of Quince Fruit and Probiotics (*Lactobacillus* and *Bifidobacterium*) on Reducing Oxidative Stress and Inflammation at the Intestinal Level and Improving Athletic Performance during Endurance Exercise. Nutrients.

[398] Singh V., Son H., Lee G., Lee S., Unno T., Shin J. (2022). Role, Relevance, and Possibilities of in Vitro Fermentation Models in Human Dietary, and Gut-microbial Studies. Biotechnol. Bioeng..

[399] Tiwari D.P., Shah P., Van Den Abbeele P., Marzorati M., Calatayud M., Ghyselinck J., Dubey A.K., Narayanan S., Jain M. (2021). Microbial Fermentation of Fossence^TM^, a Short-Chain Fructo-Oligosaccharide, under Simulated Human Proximal Colonic Condition and Assessment of Its Prebiotic Effects- A Pilot Study. FEMS Microbiol. Lett..

[400] Banerjee P., Senapati S. (2024). Translational Utility of Organoid Models for Biomedical Research on Gastrointestinal Diseases. Stem Cell Rev. Rep..

[401] Chen Y., Xing Z., Sheng J., Liu X., Ding W., Chen X. (2026). Trends in the Application of Multiomics Based on Machine Learning in the Development of Probiotics. J. Agric. Food Chem..

[402] Blasco T., Balzerani F., Valcárcel L.V., Larrañaga P., Bielza C., Francino M.P., Rufián-Henares J.Á., Planes F.J., Pérez-Burillo S. (2024). BN-BacArena: Bayesian Network Extension of BacArena for the Dynamic Simulation of Microbial Communities. Bioinformatics.

[403] Baimakhanova B.B., Sadanov A.K., Ratnikova I.A., Baimakhanova G.B., Orasymbet S.E., Amitova A.A., Aitkaliyeva G.S., Kakimova A.B. (2025). In Silico Modeling of Metabolic Pathways in Probiotic Microorganisms for Functional Food Biotechnology. Fermentation.

[404] Evdokimova S.A., Karetkin B.A., Guseva E.V., Gordienko M.G., Khabibulina N.V., Panfilov V.I., Menshutina N.V., Gradova N.B. (2022). A Study and Modeling of *Bifidobacterium* and *Bacillus* Coculture Continuous Fermentation under Distal Intestine Simulated Conditions. Microorganisms.

[405] Gonçalves B., Vilela A., Aires A., Oliveira I., Gonçalves C., Pinto T., Cosme F. (2026). Probiotic and Bioactive Compounds in Foods: From Antioxidant Properties to Gut Microbiota Modulation. Molecules.

[406] Luo L. (2025). Promoting Cognitive Health through the Nexus of Gut Microbiota and Dietary Phytochemicals. Front. Nutr..

[407] Fei Y., Zhang S., Han S., Qiu B., Lu Y., Huang W., Li F., Chen D., Berglund B., Xiao H. (2022). The Role of Dihydroresveratrol in Enhancing the Synergistic Effect of *Ligilactobacillus salivarius* Li01 and Resveratrol in Ameliorating Colitis in Mice. Research.

[408] Baştürk A., Artan R., Yılmaz A. (2016). Efficacy of Synbiotic, Probiotic, and Prebiotic Treatments for Irritable Bowel Syndrome in Children: A Randomized Controlled Trial. Turk. J. Gastroenterol..

[409] Thomas R., Williams M., Aldous J., Yanagisawa Y., Kumar R., Forsyth R., Chater A. (2022). A Randomised, Double-Blind, Placebo-Controlled Trial Evaluating Concentrated Phytochemical-Rich Nutritional Capsule in Addition to a Probiotic Capsule on Clinical Outcomes among Individuals with COVID-19—The UK Phyto-V Study. COVID.

[410] Rinaldi F., Marotta L., Mascolo A., Amoruso A., Pane M., Giuliani G., Pinto D. (2022). Facial Acne: A Randomized, Double-Blind, Placebo-Controlled Study on the Clinical Efficacy of a Symbiotic Dietary Supplement. Dermatol. Ther..

[411] Thomas R.J., Kenfield S.A., Williams M., Newton R.U., Aldous J., Mitra A., Fazili Z. (2026). Increasing Phytochemical-Rich Foods and *Lactobacillus* Probiotics in Men with Low-Risk Prostate Cancer—A Randomised, Double-Blind, Placebo-Controlled Trial. Eur. Urol. Oncol..

[412] Thomas R.J., Williams M., Aldous J.W.F., Kenfield S.A., Newton R.U. (2026). The Effect of Boosting Dietary *Lactobacillus* and Phytochemical Rich Foods on Biomarkers of Longevity—A Phase II Randomised Placebo Controlled Trial. J. Ageing Longev..

[413] Thomas R.J., Kenfield S.A., Newton P.U., Russell S., Aldous J., Williams M., Mitra A., Fazili Z. (2025). Gut Health and Prostate Cancer: The Influence of a Specific Phytochemical-Rich Food Capsule plus or Minus a Probiotic/Prebiotic Blend on Symptoms and Progression—A Randomised, Double-Blind Placebo-Controlled Trial. J. Clin. Oncol..

[414] Oliveira M., Madureira-Carvalho Á., Dinis-Oliveira R.J., Dias Da Silva D. (2025). Non-Antibiotic Therapies for Multidrug-Resistant Gastrointestinal Infections: An Overview of the Use of Probiotics, Natural Compounds, and Bacteriophages. Front. Antibiot..

[415] Hong L., Lee S.-M., Kim W.-S., Choi Y.-J., Oh S.-H., Li Y.-L., Choi S.-H., Chung D.H., Jung E., Kang S.-K. (2021). Synbiotics Containing Nanoprebiotics: A Novel Therapeutic Strategy to Restore Gut Dysbiosis. Front. Microbiol..

[416] Estevinho M.M., Yuan Y., Rodríguez-Lago I., Sousa-Pimenta M., Dias C.C., Barreiro-de Acosta M., Jairath V., Magro F. (2024). Efficacy and Safety of Probiotics in IBD: An Overview of Systematic Reviews and Updated Meta-analysis of Randomized Controlled Trials. United Eur. Gastroenterol. J..

[417] Simon E., Călinoiu L.F., Mitrea L., Vodnar D.C. (2021). Probiotics, Prebiotics, and Synbiotics: Implications and Beneficial Effects against Irritable Bowel Syndrome. Nutrients.

[418] Mofid V., Izadi A., Mojtahedi S.Y., Khedmat L. (2020). Therapeutic and Nutritional Effects of Synbiotic Yogurts in Children and Adults: A Clinical Review. Probiotics Antimicrob. Proteins.

[419] Bousdouni P., Kandyliari A., Koutelidakis A.E. (2022). Probiotics and Phytochemicals: Role on Gut Microbiota and Efficacy on Irritable Bowel Syndrome, Functional Dyspepsia, and Functional Constipation. Gastrointest. Disord..

[420] Vashisth P., Rathi N., N S.N., Mittal M. (2026). Natural Adjuncts for Prevention of Oral Diseases—A Narrative Review of Green Tea, Lemon Grass and Probiotics in Oral Health Promotion. Int. J. Drug Deliv. Technol..

[421] Yadav M., Sapra B., Dua K. (2024). Applications of Synbiotics as Cosmeceuticals. Synbiotics in Human Health: Biology to Drug Delivery.

[422] Tamer F., Kekilli M. (2024). Exploring the therapeutic potential of topical probiotics in dermatological diseases: A comprehensive review of clinical studies. J. Dtsch. Dermatol. Ges..

[423] Kumar A., Verma A. (2026). Phytoextracted Formulations for Periodontitis: Formulation Strategies, Therapeutic Mechanisms, and Clinical Applications. Macromol. Symp..

[424] Silva D.M., Costa P.A.D., Ribon A.O.B., Purgato G.A., Gaspar D.-M., Diaz M.A.N. (2019). Plant Extracts Display Synergism with Different Classes of Antibiotics. An. Acad. Bras. Ciênc..

[425] AL-siraj S.S., Badr J.M., Abd El-Tawab A.A. (2024). Antibacterial Potentials of Some Plants Extracts and Their Combinations and Their Synergistic Activities against Multidrug Resistant Bacteria. Bull. Pharm. Sci. Assiut Univ..

[426] Lee S., Choi S.-P., Choi H.-J., Jeong H., Park Y.-S. (2024). A Comprehensive Review of Synbiotics: An Emerging Paradigm in Health Promotion and Disease Management. World J. Microbiol. Biotechnol..

[427] Baheti R., Deshkar S., Jadhav S., Mule K., Jha A., Giram P., Mahore J. (2026). Interplay of Probiotics, Prebiotics, Synbiotics and Postbiotics: A Review of Their Therapeutic Potential for Gastrointestinal Inflammation. Food Res. Int..

[428] Śliżewska K., Chlebicz-Wójcik A. (2020). The In Vitro Analysis of Prebiotics to Be Used as a Component of a Synbiotic Preparation. Nutrients.

[429] Salem H.A., Rizk N.I., AbdelSalam M.H., Ahmed R., Atteia H.H., Hamdan A.M.E., Alghamdi A.A., Alghusn M.A., Alatawi R.A., Atallah R.A. (2026). Therapeutic Effects of *Lactobacillus Rhamnosus*, Thymol and Their Combination against Neurotoxicity in Propionic Acid (PA)-Induced Autistic Rats: Insights into the Role of the Nrf2/HO-1, Wnt3/β-Catenin/GSK3β BDNF/p-TrkB/CREB, pI3K/Akt/mTOR, AMPK/SIRT-1, and PERK/CHOP/Bcl-2 Pathways. Front. Pharmacol..

[430] Wang Z., Jin X., Zhang X., Xie X., Tu Z., He X. (2024). Corrigendum: From Function to Metabolome: Metabolomic Analysis Reveals the Effect of Probiotic Fermentation on the Chemical Compositions and Biological Activities of *Perilla Frutescens* Leaves. Front. Nutr..

[431] Rupasinghe H.P.V., Parmar I., Neir S.V. (2019). Biotransformation of Cranberry Proanthocyanidins to Probiotic Metabolites by *Lactobacillus Rhamnosus* Enhances Their Anticancer Activity in HepG2 Cells In Vitro. Oxidative Med. Cell. Longev..

[432] Zhang J., Huang X., Cheng J., Wang C. (2023). Effect of *Lactobacillus* (*L. Acidophilus* NCIB1899, *L. Casei* CRL 431, *L. Paracasei* LP33) Fermentation on Free and Bound Polyphenolic, Antioxidant Activities in Three *Chenopodium Quinoa* Cultivars. J. Food Sci..

[433] Loo J.S., Oslan S.N.H., Mokshin N.A.S., Othman R., Amin Z., Dejtisakdi W., Prihanto A.A., Tan J.S. (2025). Comprehensive Review of Strategies for Lactic Acid Bacteria Production and Metabolite Enhancement in Probiotic Cultures: Multifunctional Applications in Functional Foods. Fermentation.

[434] Kitir Sen N., Kocaman A., Aydemir Ö.E. (2025). The Role of Chemical Fertilizer Reduction and Different Microbial Inoculants on Yield Increase in Lettuce Cultivation. BMC Plant Biol..

[435] Rezaee Danesh Y., Mulet J.M., Porcel R. (2025). Bridging Microbial Biocontrol and Phytochemical Biopesticides: Synergistic Approaches for Sustainable Crop Protection. Plants.

[436] Jouga F., Mourabiti F., Soukri A., De Miguel T., El Khalfi B. (2026). Natural Biotics as Biocontrol Agents for Sustainable Aquaculture. Appl. Sci..

[437] Khanjani M.H., Sharifinia M., Akhavan-Bahabadi M., Emerenciano M.G.C. (2024). Probiotics and Phytobiotics as Dietary and Water Supplements in Biofloc Aquaculture Systems. Aquac. Nutr..

[438] Romero J., Albertos I., Díez-Méndez A., Poveda J. (2022). Control of Postharvest Diseases in Berries through Edible Coatings and Bacterial Probiotics. Sci. Hortic..

[439] Sharma V., Salwan R., Sharma P.N. (2017). The Comparative Mechanistic Aspects of Trichoderma and Probiotics: Scope for Future Research. Physiol. Mol. Plant Pathol..

[440] D’Accolti M., Soffritti I., Bini F., Mazziga E., Mazzacane S., Caselli E. (2022). Pathogen Control in the Built Environment: A Probiotic-Based System as a Remedy for the Spread of Antibiotic Resistance. Microorganisms.

[441] Peruzzolo M., Ceni G.C., Junges A., Zeni J., Cansian R.L., Backes G.T. (2025). Probiotics: Health Benefits, Microencapsulation, and Viability, Combination with Natural Compounds, and Applications in Foods. Food Biosci..

[442] Bhutto R.A., Bhutto N.U.A.H., Mahar H., Khanal S., Wang M., Iqbal S., Fan Y., Yi J. (2025). Recent Trends in Co-Encapsulation of Probiotics with Prebiotics and Their Applications in the Food Industry. Trends Food Sci. Technol..

[443] Misra S., Pandey P., Mishra H.N. (2021). Novel Approaches for Co-Encapsulation of Probiotic Bacteria with Bioactive Compounds, Their Health Benefits and Functional Food Product Development: A Review. Trends Food Sci. Technol..

[444] Jin Z., Wang Y. (2026). Recent Progress in Probiotic Encapsulation: Techniques, Characterization and Food Industry Prospects. Foods.

[445] Jacobsen C., García-Moreno P.J., Mendes A.C., Mateiu R.V., Chronakis I.S. (2018). Use of Electrohydrodynamic Processing for Encapsulation of Sensitive Bioactive Compounds and Applications in Food. Annu. Rev. Food Sci. Technol..

[446] Silva M.P., Martelli-Tosi M., Massarioli A.P., Melo P.S., Alencar S.M., Favaro-Trindade C.S. (2022). Co-Encapsulation of Guaraná Extracts and Probiotics Increases Probiotic Survivability and Simultaneously Delivers Bioactive Compounds in Simulated Gastrointestinal Fluids. LWT.

[447] Osojnik Črnivec I.G., Poklar Ulrih N. (2019). Nano-Hydrogels of Alginate for Encapsulation of Food Ingredients. Biopolymer Nanostructures for Food Encapsulation Purposes.

[448] Espín J.C., Jarrín-Orozco M.P., Osuna-Galisteo L., Ávila-Gálvez M.Á., Romo-Vaquero M., Selma M.V. (2024). Perspective on the Coevolutionary Role of Host and Gut Microbiota in Polyphenol Health Effects: Metabotypes and Precision Health. Mol. Nutr. Food Res..

[449] Jiménez-González C., Alonso-Peña M., Argos Vélez P., Crespo J., Iruzubieta P. (2025). Unraveling MASLD: The Role of Gut Microbiota, Dietary Modulation, and AI-Driven Lifestyle Interventions. Nutrients.

[450] Catinean A., Neag M.A., Muntean D.M., Bocsan I.C., Buzoianu A.D. (2018). An Overview on the Interplay between Nutraceuticals and Gut Microbiota. PeerJ.

[451] Kallapura G., Prakash A.S., Sankaran K., Manjappa P., Chaudhary P., Ambhore S., Dhar D. (2024). Microbiota Based Personalized Nutrition Improves Hyperglycaemia and Hypertension Parameters and Reduces Inflammation: A Prospective, Open Label, Controlled, Randomized, Comparative, Proof of Concept Study. PeerJ.

[452] Fatima G., Khan S., Shukla V., Awaida W., Li D., Gushchina Y.S. (2025). Nutraceutical Formulations and Natural Compounds for the Management of Chronic Diseases. Front. Nutr..

[453] Schmelz R.M., Dayan N. (2020). Regulation of Probiotic and Other Live Biologic Products: The United States Approach. Skin Microbiome Handbook.

[454] Usmani A., Almoselhy R.I.M., Mishra A., Rajendran R., Thomas S. (2026). Role of Biodegradable Synthetic and Food-Derived Polymers for Effective Delivery of Nutraceuticals. Handbook of Nutraceuticals.

[455] Dronkers T.M.G., Krist L., Van Overveld F.J., Rijkers G.T. (2018). The Ascent of the Blessed: Regulatory Issues on Health Effects and Health Claims for Probiotics in Europe and the Rest of the World. Benef. Microbes.

[456] Shishir M.R.I., Khan S., Karim N., Bilal M., Hussain S., Saifullah M., Rashwan A.K., Tahir H.E., Mohamed Ahmed I.A., Zhang X. (2026). New Frontiers in Beverage Fortification: Delivery of Functional Ingredients via Micro- and Nanoencapsulation in Dairy and Non-Dairy Beverages. Food Res. Int..

[457] Santhiravel S., Bekhit A.E.-D.A., Mendis E., Jacobs J.L., Dunshea F.R., Rajapakse N., Ponnampalam E.N. (2022). The Impact of Plant Phytochemicals on the Gut Microbiota of Humans for a Balanced Life. Int. J. Mol. Sci..

[458] Kothari D., Patel S., Kim S.-K. (2019). Probiotic Supplements Might Not Be Universally-Effective and Safe: A Review. Biomed. Pharmacother..

[459] Vernocchi P., Del Chierico F., Putignani L. (2016). Gut Microbiota Profiling: Metabolomics Based Approach to Unravel Compounds Affecting Human Health. Front. Microbiol..

[460] De Deus C., Duque-Soto C., Rueda-Robles A., Martínez-Baena D., Borrás-Linares I., Quirantes-Piné R., Ragagnin De Menezes C., Lozano-Sánchez J. (2024). Stability of Probiotics through Encapsulation: Comparative Analysis of Current Methods and Solutions. Food Res. Int..

[461] Ji C., Li D., Liang Y., Luo Y. (2025). Co-Encapsulation of Probiotics with Functional Components: Design Strategies, Synergistic Mechanisms, Biomedical Applications, and Challenges for Industrialization. J. Mater. Chem. B.

[462] D’Amico V., Cavaliere M., Ivone M., Lacassia C., Celano G., Vacca M., La Forgia F.M., Fontana S., De Angelis M., Denora N. (2025). Microencapsulation of Probiotics for Enhanced Stability and Health Benefits in Dairy Functional Foods: A Focus on Pasta Filata Cheese. Pharmaceutics.

[463] Spacova I., Binda S., Ter Haar J.A., Henoud S., Legrain-Raspaud S., Dekker J., Espadaler-Mazo J., Langella P., Martín R., Pane M. (2023). Comparing Technology and Regulatory Landscape of Probiotics as Food, Dietary Supplements and Live Biotherapeutics. Front. Microbiol..

[464] Colombo F., Restani P., Biella S., Di Lorenzo C. (2020). Botanicals in Functional Foods and Food Supplements: Tradition, Efficacy and Regulatory Aspects. Appl. Sci..

[465] Santini A., Cammarata S.M., Capone G., Ianaro A., Tenore G.C., Pani L., Novellino E. (2018). Nutraceuticals: Opening the Debate for a Regulatory Framework. Br. J. Clin. Pharmacol..

[466] Binda S., Hill C., Johansen E., Obis D., Pot B., Sanders M.E., Tremblay A., Ouwehand A.C. (2020). Criteria to Qualify Microorganisms as “Probiotic” in Foods and Dietary Supplements. Front. Microbiol..

[467] Wu Q., Kan J., Cui Z., Ma Y., Liu X., Dong R., Huang D., Chen L., Du J., Fu C. (2025). Understanding the Nutritional Benefits through Plant Proteins-Probiotics Interactions: Mechanisms, Challenges, and Perspectives. Crit. Rev. Food Sci. Nutr..

[468] Kim H.K., Kim S.J., Gil W.J., Yang C.-S. (2025). Exploring the Therapeutic Potential of Phytochemicals: Challenges and Strategies for Clinical Translation. Phytomedicine.

[469] Fiebig M.S., Prestes A.A., Carvalho A.C., De Souza C.K., Prudencio E.S. (2026). Functional Cheeses Enhanced with Native Plants: Relevance and Future Perspectives. Curr. Opin. Food Sci..

[470] Bolzon V., Pesando M., Bulfoni M., Nencioni A., Nencioni E. (2022). An Integrated Analytical Approach for the Characterization of Probiotic Strains in Food Supplements. Nutrients.

[471] Boronat A., Rodriguez-Morató J., Serreli G., Fitó M., Tyndale R.F., Deiana M., de la Torre R. (2021). Contribution of Biotransformations Carried Out by the Microbiota, Drug-Metabolizing Enzymes, and Transport Proteins to the Biological Activities of Phytochemicals Found in the Diet. Adv. Nutr..

[472] De Bellis P., Sisto A., Lavermicocca P. (2021). Probiotic Bacteria and Plant-Based Matrices: An Association with Improved Health-Promoting Features. J. Funct. Foods.

[473] Idakwoji P.A., Tukur U., Onwukwe B.O., Agboola J.B., Onugwu E.O., Iyiola A.T., Amadi B.E., Onoja A.O., Ali A.S., Ojo S.S. (2026). Phytochemicals as Promising Lead Compounds for Drug Discovery: A Comprehensive Review of Mechanisms and Pharmacological Potentials. Lett. Drug Des. Discov..

[474] Akhila S., Remya K. (2025). Phytochemicals in Liver Cancer-Translational Limitations and Strategies. Liver Cancer and Phytomedicine.

[475] Vijayaganapathi A., Mohanasrinivasan V. (2025). A Review of Next-Generation Probiotics—As a Gateway to Biotherapeutics. Probiotics Antimicrob. Proteins.

[476] Parvin N., Aslam M., Joo S.W., Mandal T.K. (2025). Nano-Phytomedicine: Harnessing Plant-Derived Phytochemicals in Nanocarriers for Targeted Human Health Applications. Molecules.

[477] Martelli A., Mohamed Y.O.A., Gallego-Ferrer G., Gentile P., Girón-Hernández J. (2025). Revolutionizing Gut Health: Advances in Encapsulation Strategies for Probiotics and Bioactive Molecules. Biotechnol. Adv..

[478] Varsha K.K., Maheshwari A.P., Nampoothiri K.M. (2021). Accomplishment of Probiotics in Human Health Pertaining to Immunoregulation and Disease Control. Clin. Nutr. ESPEN.

[479] Husain A., Khanam A., Khan H., Rafi Z., Shahab U., Rab S.O., Puri P., Ahmad S. (2026). Probiotics as a Strategy to Mitigate Glycation: Mechanisms and Strain-Specific Effects on Advanced Glycation End-Products-Related Diseases. J. Food Sci..

[480] Zhang L., Virgous C., Si H. (2019). Synergistic Anti-Inflammatory Effects and Mechanisms of Combined Phytochemicals. J. Nutr. Biochem..

[481] De Almeida Veeck I.C., De Menezes C.R., Jacob-Lopes E., Queiroz M.I., Zepka L.Q., Deprá M.C. (2026). Co-Encapsulation of Probiotics and Microalgae Pigments. Pigments from Microalgae Handbook—Volume II.

[482] Prakash G., Chaudhary A.A., Tanu R., Ali M.A.M., Boufahja F., Sharma P.K., Lakhawat S.S., Yadav T., Upadhyay N.K., Kumar V. (2026). Harnessing Phytochemicals and Nanotechnology Synergy for Molecular, Epigenetic, and Microbiota-Driven Regulation in Type 2 Diabetes Mellitus. Pharmaceutics.

[483] Abbas M.S., Saeed F., Afzaal M., Jianfeng L., Hussain M., Ikram A., Jabeen A. (2022). Recent Trends in Encapsulation of Probiotics in Dairy and Beverage: A Review. Food Process. Preserv..

[484] Liang Z., Liang Z., Hu H., Howell K., Fang Z., Zhang P. (2025). Food Substances Alter Gut Resistome: Mechanisms, Health Impacts, and Food Components. Compr. Rev. Food Sci. Food Safe.

[485] Abavisani M., Khoshroo N., Tafti P., Karbas Foroushan S., Ebadpour N., Karav S., Kesharwani P., Sahebkar A. (2025). Mechanisms, Modulation, and Mitigation: Dietary-Gut Microbiome Strategies Against Antibiotic Resistance. Probiotics Antimicrob. Proteins.

[486] Imchen M., Moopantakath J., Kumavath R., Barh D., Tiwari S., Ghosh P., Azevedo V. (2020). Current Trends in Experimental and Computational Approaches to Combat Antimicrobial Resistance. Front. Genet..

[487] Azeredo J., Azevedo N.F., Briandet R., Cerca N., Coenye T., Costa A.R., Desvaux M., Di Bonaventura G., Hébraud M., Jaglic Z. (2017). Critical Review on Biofilm Methods. Crit. Rev. Microbiol..

[488] Vyas H.K.N., Xia B., Mai-Prochnow A. (2022). Clinically Relevant in Vitro Biofilm Models: A Need to Mimic and Recapitulate the Host Environment. Biofilm.

[489] Nayan S., Baghel Chauhan S., Singh I., Jain C. (2026). Unravelling the Regulatory Paradox of Probiotics: Challenges in Standardization, Clinical Validation, and Global Acceptance. Recent Pat. Food Nutr. Agric..

[490] Churin A.A., Sokolyanskaya L.O., Lukina A.P., Karnachuk O.V. (2026). Current Concepts in Probiotic Safety and Efficacy. Nutrients.

[491] Westfall S., Carracci F., Estill M., Zhao D., Wu Q., Shen L., Simon J., Pasinetti G.M. (2021). Optimization of Probiotic Therapeutics Using Machine Learning in an Artificial Human Gastrointestinal Tract. Sci. Rep..

[492] Kwoji I.D., Aiyegoro O.A., Okpeku M., Adeleke M.A. (2023). ‘Multi-Omics’ Data Integration: Applications in Probiotics Studies. npj Sci. Food.

[493] Rebollar E.A., Antwis R.E., Becker M.H., Belden L.K., Bletz M.C., Brucker R.M., Harrison X.A., Hughey M.C., Kueneman J.G., Loudon A.H. (2016). Using “Omics” and Integrated Multi-Omics Approaches to Guide Probiotic Selection to Mitigate Chytridiomycosis and Other Emerging Infectious Diseases. Front. Microbiol..

[494] Phan J., Calvo D.C., Nair D., Jain S., Montagne T., Dietsche S., Blanchard K., Treadwell S., Adams J., Krajmalnik-Brown R. (2024). Precision Synbiotics Increase Gut Microbiome Diversity and Improve Gastrointestinal Symptoms in a Pilot Open-Label Study for Autism Spectrum Disorder. mSystems.

[495] Vandeputte D. (2020). Personalized Nutrition Through The Gut Microbiota: Current Insights And Future Perspectives. Nutr. Rev..

[496] Li P., Luo H., Ji B., Nielsen J. (2022). Machine Learning for Data Integration in Human Gut Microbiome. Microb. Cell Fact..

[497] Manach C., Milenkovic D., Van de Wiele T., Rodriguez-Mateos A., de Roos B., Garcia-Conesa M.T., Landberg R., Gibney E.R., Heinonen M., Tomás-Barberán F. (2017). Addressing the Inter-Individual Variation in Response to Consumption of Plant Food Bioactives: Towards a Better Understanding of Their Role in Healthy Aging and Cardiometabolic Risk Reduction. Mol. Nutr. Food Res..

[498] Sanders M.E., Merenstein D.J., Reid G., Gibson G.R., Rastall R.A. (2019). Probiotics and Prebiotics in Intestinal Health and Disease: From Biology to the Clinic. Nat. Rev. Gastroenterol. Hepatol..

[499] Marco M.L., Cunningham M., Bischoff S.C., Clarke G., Delzenne N., Lewis J.D., Meisel M., Merenstein D., O’Toole P.W., Staudacher H.M. (2026). The International Scientific Association for Probiotics and Prebiotics (ISAPP) Consensus Statement on the Definition and Scope of Gut Health. Nat. Rev. Gastroenterol. Hepatol..

[500] Salminen S., Collado M.C., Endo A., Hill C., Lebeer S., Quigley E.M.M., Sanders M.E., Shamir R., Swann J.R., Szajewska H. (2021). The International Scientific Association of Probiotics and Prebiotics (ISAPP) Consensus Statement on the Definition and Scope of Postbiotics. Nat. Rev. Gastroenterol. Hepatol..

[501] Das J., Bhui U., Shil S., Mondal M., Taghizadeh-Hesary F., Ahuja V., Tiwari K., Abdi G. (2026). Automated Real-Time Screening of Phytochemicals and Cancer Therapeutics. Sustainable Healthcare.

[502] Cacace E., Kim V., Varik V., Knopp M., Tietgen M., Brauer-Nikonow A., Inecik K., Mateus A., Milanese A., Mårli M.T. (2023). Systematic Analysis of Drug Combinations against Gram-Positive Bacteria. Nat. Microbiol..

[503] Jusková P., Kling A., Schmitt S., Dittrich P.S., Taly V., Descroix S., Perez-Toralla K. (2024). A Thermoplastic Microsystem to Perform Antibiotic Susceptibility Testing by Monitoring Oxygen Consumption. Microfluidics Diagnostics.

[504] Kapoor A., Chaudhari P., Awasthi A., Nayak J., Kannan D. (2025). Advances in Microfluidics for Detection of Infectious Diseases. Advances in Separation Sciences.

[505] Liu J., Du H., Huang L., Xie W., Liu K., Zhang X., Chen S., Zhang Y., Li D., Pan H. (2024). AI-Powered Microfluidics: Shaping the Future of Phenotypic Drug Discovery. ACS Appl. Mater. Interfaces.

[506] Diao Z., Peng Q., Luo S., Kan L., Ge A., Gao W., Li R., Bao W., Wang X., Ji Y. (2025). AI-Powered High-Throughput Digital Colony Picker Platform for Sorting Microbial Strains by Multi-Modal Phenotypes. Nat. Commun..

[507] Olcay B., Ozdemir G.D., Ozdemir M.A., Ercan U.K., Guren O., Karaman O. (2024). Prediction of the Synergistic Effect of Antimicrobial Peptides and Antimicrobial Agents via Supervised Machine Learning. BMC Biomed. Eng..

[508] Besharatifard M., Vafaee F. (2024). A Review on Graph Neural Networks for Predicting Synergistic Drug Combinations. Artif. Intell. Rev..

[509] Mostafa M.A.H., Khojah H.M.J. (2025). Nanoparticle-Based Delivery Systems for Phytochemicals in Cancer Therapy: Molecular Mechanisms, Clinical Evidence, and Emerging Trends. Drug Dev. Ind. Pharm..

[510] Nopparatmaitree M., Hwanhlem N., Mitsuwan W., Thongnum A., Intawicha P., Loor J.J., Incharoen T. (2026). Novel Double-Layer Microencapsulated Phytosynbiotic Derived from Probiotics and Tiliacora Triandra Extract for Application in Broiler Production. Fermentation.

[511] Bohórquez León D.L., Gómez Castaño J.A., Otálora M.C., Martinez-Galan J.P. (2025). Microencapsulation of *Saccharomyces Boulardii* CNCM I-745 in Coffee Mucilage and/or Inulin as Wall Material by Spray-Drying. Food Biosci..

[512] Bakr R., Abdelmoteleb A., Mendez-Trujillo V., Gonzalez-Mendoza D., Hewedy O. (2025). The Potential of Beneficial Microbes for Sustainable Alternative Approaches to Control Phytopathogenic Diseases. Microbiol. Res..

[513] Sharma S., Sanasam J., Thingujam U., Yendrembam K., Maisnam G., Tomar A., Kumar A., González Estrada R.R., Parmar K. (2025). Commercialization Aspects of Microbial Biocontrol Agents. Microbial Consortia in Plant Science and Sustainable Agriculture.

[514] Valenzuela Ruiz V., Cervantes Enriquez E.P., Vázquez Ramírez M.F., Bivian Hernández M.D.L.Á., Cárdenas-Manríquez M., Parra Cota F.I., De Los Santos Villalobos S. (2025). A New Era in the Discovery of Biological Control Bacteria: Omics-Driven Bioprospecting. Soil Syst..

[515] Guangxin G., Li K., Zhu Q., Zhao C., Li C., He Z., Hu S., Ren Y. (2022). Improvements of Immune Genes and Intestinal Microbiota Composition of Turbot (*Scophthalmus maximus*) with Dietary Oregano Oil and Probiotics. Aquaculture.

[516] Chielle E.O., Vecchia D.D., Rossi E.M., Chielle A.P.O., Bonadiman B.D.S.R., Marafon F., Bagatini M.D. (2022). Supplementation with Detox Juice Added with Probiotic Improves Atherogenic Parameters in Healthy Individuals. Braz. J. Pharm. Sci..

[517] Napier B.A., Allegretti J.R., Feuerstadt P., Kelly C.R., Van Hise N.W., Jäger R., Kassam Z., Reid G. (2025). Multi-Species Synbiotic Supplementation Enhances Gut Microbial Diversity, Increases Urolithin A and Butyrate Production, and Reduces Inflammation in Healthy Adults: A Randomized, Placebo-Controlled Trial. Nutrients.

[518] Hatfull G.F., Dedrick R.M., Schooley R.T. (2022). Phage Therapy for Antibiotic-Resistant Bacterial Infections. Annu. Rev. Med..

[519] Kortright K.E., Chan B.K., Koff J.L., Turner P.E. (2019). Phage Therapy: A Renewed Approach to Combat Antibiotic-Resistant Bacteria. Cell Host Microbe.

[520] Subramanian A. (2024). Emerging Roles of Bacteriophage-Based Therapeutics in Combating Antibiotic Resistance. Front. Microbiol..

[521] Bumunang E.W., Ateba C.N., Stanford K., Niu Y.D., Wang Y., McAllister T.A. (2020). Activity of Bacteriophage and Complex Tannins against Biofilm-Forming Shiga Toxin-Producing *Escherichia Coli* from Canada and South Africa. Antibiotics.

[522] Maan S.A., Faiesal A.A., Gamar G.M., El Dougdoug N.K. (2025). Efficacy of Bacteriophages with Aloe Vera Extract in Formulated Cosmetics to Combat Multidrug-Resistant Bacteria in Skin Diseases. Sci. Rep..

[523] Stachurska X., Mizielińska M., Ordon M., Nawrotek P. (2023). Combinations of Echinacea (*Echinacea purpurea*) and Rue (*Ruta gravolens*) Plant Extracts with Lytic Phages: A Study on Interactions. Appl. Sci..

[524] Yousuf M., Farooq H., Shah Q. (2024). Combined Antibacterial Activity of Bacteriophages and Extract of Caesalpinia Decapetala and Parrotiopsis Jacquemontiana Against Pseudomonas Aeruginosa and Staphylococcus Aureus. Microbiol. Immunol. Commun..

